# Soy-based purified ingredient diet affects mouse gut permeability and the microbiome in fragile X mice

**DOI:** 10.3389/fnmol.2025.1520211

**Published:** 2025-03-21

**Authors:** Cara J. Westmark

**Affiliations:** ^1^Department of Neurology, University of Wisconsin, Madison, WI, United States; ^2^Molecular Environmental Toxicology Center, University of Wisconsin, Madison, WI, United States

**Keywords:** fragile X syndrome, FITC-dextran, gut permeability, microbiome, mouse, soy

## Abstract

**Introduction:**

Gastrointestinal problems including vomiting, reflux, flatulence, diarrhea, constipation and colic are common comorbidities in fragile X syndrome. There is accumulating evidence suggesting that leaky gut syndrome causes neurological phenotypes. Although fragile X messenger ribonucleoprotein is ubiquitously expressed, there is a dearth of knowledge regarding its role outside of the brain including effects on gut dysfunction in fragile X. The aim of this study was to generate novel data on gastrointestinal barrier function and the gut microbiome in response to *Fmr1* genotype, sex and diet in mice.

**Methods:**

*Fmr1^KO^* male mice and littermate controls in an FVB background were maintained on two purified ingredient diets (AIN-93G with casein protein versus soy protein isolate) versus two standard chows (Teklad 2019 with wheat, corn and yeast protein versus Purina 5015 with wheat, soy, corn, yeast and whey protein sources). Gut permeability was quantified by FITC-dextran levels in blood plasma. The cecal microbiome was identified by 16S rRNA sequencing. In addition, gut permeability was tested in *Fmr1^KO^* mice in the C57BL/6 J background maintained on casein- and soy protein isolate-based AIN-93G versus Teklad 2019.

**Results:**

Knockout of the *Fmr1* gene in FVB mice did not affect gut permeability. Soy protein isolate-based AIN-93G increased gut permeability. Beta-diversity of the cecal microbiome was significantly altered as a function of the four test diets. *Akkermansia_muciniphila* was increased in *Fmr1^KO^* mice fed AIN-93G while unnamed species within the genus *Anaerovorax* and family Ruminococcaceae were increased and the order Clostridales decreased in *Fmr1^KO^* mice fed AIN-93G/soy. *Fmr1^KO^* mice in the C57BL/6 J background exhibited increased gut permeability in response to soy protein.

**Discussion:**

These findings regarding the effects of diet on gut permeability and the microbiome have important implications for experimental design. Single-source diets are ubiquitously used to maintain laboratory animals for medical research and feed details are frequently not reported in publications. Diet/phenotype interactions could have a large impact on inter-laboratory replicability in premedical research. For infants with fragile X, early-life diet could impact the severity of disease outcomes.

## 1 Introduction

Gastrointestinal problems and an altered gut microbiota are prevalent phenotypes in individuals with autism spectrum disorder (Li et al., 2017). Targeting gut permeability and microbiota are receiving increasing attention as a personalized medicine approach for autism. Fragile X syndrome (FXS) is the leading known genetic cause of autism (Reddy, 2005; Hagerman et al., 2010), caused by a trinucleotide repeat expansion mutation in the 5′-untranslated region of the fragile X messenger ribonucleoprotein 1 (*FMR1*) gene located on the X-chromosome (Verkerk et al., 1991). The mutation silences transcription of the promoter resulting in loss of expression of fragile X messenger ribonucleoprotein (FMRP) (Oberlé et al., 1991; Verkerk et al., 1991). The *FMR1* gene was discovered in 1991 and medical research has primarily focused on the function of FMRP in the brain in relation to translational regulation and synapse development (Davis and Broadie, 2017). The role of FMRP in the periphery, including the gut, the gut-brain axis, and the microbiome remains largely unexplored.

This study tests the hypothesis that gut permeability and the microbiome differ as a function of *Fmr1 g*enotype and soy protein in mice. To our knowledge, we are the only laboratory who has studied the effect of dietary protein on *Fmr1^KO^* phenotypes. Our prior research indicates that single-source soy-based diets increase seizure susceptibility and body weight in mice with more pronounced effects in males than females (Westmark et al., 2013, 2022a,b, 2024). Increased body mass is due to increased fat mass and total body area in *Fmr1^KO^* females and increased lean mass and bone mineral density in *Fmr1^KO^* males. Soy protein induces sex-specific differences in activity levels. Specifically, females exhibit hyperactivity with the transition to lights on whereas males show elevated plasma phytoestrogen levels. Two phenotypes, activity levels at the beginning of the light cycle and testes weight, are exacerbated in *Fmr1^KO^* versus WT males irrespective of diet (Westmark et al., 2024). Medical record and survey analyses demonstrate associations between soy-based infant formula and increased incidence of seizures, autistic behaviors, allergies and gastrointestinal problems in autism and/or FXS (Westmark, 2013, 2014a,b, 2016, 2021, 2022; Westmark et al., 2020). Overall, there is accumulating evidence suggesting differential developmental outcomes in mice and humans in response to single-source, soy-based diets. The mechanism remains unknown but soy phytoestrogens are likely involved (Westmark, 2014a,b, 2016, 2023; Ariyani and Koibuchi, 2024).

Diet is an important environmental variable that is frequently not considered in experimental design nor reported in publications. Research mice are normally fed low cost, single-source diets that contain bioactive ingredients that may affect study variables and contribute to the current crisis in non-reproducible research. The aim of this study was to generate novel data on gastrointestinal barrier function and the gut microbiome in *Fmr1^KO^* and littermate mice as a function of diet. Two chows (Teklad 2019 and Purina 5015) and two purified ingredient diets (casein versus soy protein-based AIN-93G) were compared. These diets were chosen because Teklad 2019 and Purina 5015 are frequently used for routine maintenance of mice and AIN-93G for testing drug supplementation. The Purina 5015 and AIN-93G/soy contain soy protein, but Teklad 2019 and AIN-93G do not.

## 2 Methods

### 2.1 Mouse husbandry

*Fmr1^KO^* breeding pair were purchased from Jackson Laboratories, FVB.129P2-Pde6b < +> Tyr < c-ch > Fmr1 < tm1Cgr>/J (stock number 004624), hereafter referred to as FVB *Fmr1^KO^*. FVB *Fmr1^KO^* males were mated with WT females (FVB.129P2-Pde6b < +> Tyr < c-ch>/AntJ, Jackson Laboratories stock number 004828) to generate FVB *Fmr1^HET^* females and FVB WT males, which were bred to generate and maintain a colony of *Fmr1* and littermate mice in the FVB background. FVB *Fmr1^HET^* females and FVB *Fmr1^KO^* or WT males were transferred to test diets at least 2 weeks prior to breeding. Breeding pairs were housed in microisolator cages on 12 h (0600–1800) light cycle with *ad libitum* access to food, water and Shepherd’s® Cob Plus™ bedding. Specific diets are described below. Offspring were weaned onto the same diets. *Fmr1^KO^* mice in the C57BL/6 J background have been maintained at the University of Wisconsin-Madison for over 20 years (Westmark et al., 2024). The C57BL/6 J *Fmr1^KO^* used in this study were maintained on their respective diets for over ten generations. Mouse genotypes were determined by automated DNA extraction and real-time PCR analysis by Transnetyx (Memphis, TN) of tail biopsies taken at weaning and euthanasia. The animal study protocol was approved by the Institutional Animal Care and Use Committee (IACUC) at the University of Wisconsin, Madison (protocol code M005224).

### 2.2 Diets

Test diets included two purified ingredient diets formulated by Envigo and two standard chows (Supplementary File S1). The purified ingredient diets included casein-based TD.180374, which is a modification of AIN-93G (Envigo TD.94045) with increased sodium at 2 g/kg diet (0.2%) to match the sodium content of soy protein isolate (SPI)-based AIN-93G, TD.180375. TD.180374 contains 17.7% protein by percent weight, 59.8% carbohydrate and 7.2% fat corresponding to 18.9, 63.8, and 17.3%, respectively, % kcal with a total energy density of 3.7 kcal/g. The major ingredients are corn starch, casein, maltodextrin and sucrose. Red food dye was added for visual differentiation. TD.180375 is modified from AIN-93G to replace casein with SPI and to match micronutrients to the control diet TD.180374 including 0.5% calcium, 0.3% available phosphorus, 0.2% sodium, 0.36% potassium, 0.3% chloride and 0.05% magnesium. TD.180375 contains 17.8% protein by % weight, 60.7% carbohydrate and 7.2% fat corresponding to 18.8, 64.1 and 17.1%, respectively, % kcal with a total energy density of 3.8 kcal/g. Green food dye was added for visual differentiation. TD.180374 and TD.180375 were portioned into sealed Tupperware containers and stored at 4°C prior to use. Standard chows included Teklad 2019 (Envigo, Fitchburg, WI, United States), which is a fixed formula diet with a nutritional profile of 19.0% protein, 9.0% fat, 2.6% fiber, 12.1% neutral detergent fiber, 5.0% ash, and 44.9% carbohydrate. The main ingredients are ground wheat, ground corn, corn gluten meal and wheat middlings. The energy density is 3.3 kcal/kg. The other standard chow was Purina 5015 (LabDiet, St. Louis, MO, United States), which is a complete life cycle diet with a nutritional profile of 19% protein, 11% fat, 2.4% fiber, and 52% carbohydrate. The main ingredients are whole wheat, dehulled soybean mean and ground corn. The energy density is 3.60 kcal/g. These diets were chosen to test the effects of soy protein in the context of a matched purified ingredient diet and a chow. Protein source is the major the difference between the four diets. AIN-93G and AIN-93G/soy are matched except for protein source, casein versus soy. Purina 5015 contains soy protein but Teklad 2019 does not.

### 2.3 Gut permeability test

Gut permeability was assessed by FITC-dextran transit from the intestines to the blood. Briefly, mice postnatal day 70 (P70) were transferred to a clean cage with a water bottle but no bedding or food and fasted at least 4 h prior to oral gavage with FITC-dextran. FITC-D4000 (Sigma Aldrich, Co., St. Louis, MO, catalog #46944-500MG-F) was dissolved in Dulbecco’s phosphate buffered saline without calcium and magnesium (DPBS, Mediatech Inc., Manassas, VA) at 25 mg/mL, stored at 4°C, and dosed by oral gavage at 500 mg/kg based on mouse body weight. Mice were anesthetized with isoflurane (~2% inhalation) 1 h after oral gavage. Blood was collected from the inferior vena cava into Eppendorf tubes containing 20 μL 10 mg/mL sodium heparin and mixed to prevent coagulation. After blood was collected from all animals for the day, samples were spun at 5,000 rpm for 10 min at room temperature and the upper plasma layer was transferred to an Eppendorf tube. Plasma (50 μL) was diluted with 100 μL DPBS. A standard curve was prepared by serial dilution of FITC-D4000 in the range of 0.1–12.5 μg/mL with 33% mouse plasma (500 μL diluted with 1 mL DPBS). Control mouse plasma was obtained on the same day from fasted littermate mice not treated with FITC-D4000. Standards and samples (100 μL each) were transferred to wells of a 96-well clear plate and fluorescence was measured on a Biotek Synergy/neo2 plate reader at an excitation of 485 nm and emission 528 nm with both AUTO sensitivity and a gain of 60. To calculate FITC-dextran permeability, the reading for the diluent blank was subtracted from the standard and sample readings and a standard curve was generated by plotting concentration (x-axis) versus fluorescence (y-axis) of the FITC-D4000 standards followed by linear regression analysis of the samples. Analysis of Variance (ANOVA) was conducted with GraphPad Prism 10 for macOS, version 10.3.1 software for two-way ANOVA analysis of genotype (male WT and *Fmr1^KO^* and female WT, *Fmr1^HET^* and *Fmr1^KO^*) and diet (AIN-93G, AIN-93G/soy, Teklad 2019, Purina 5015) for FVB mice and for two-way ANOVA analysis of genotype (male and female *Fmr1^KO^*) and diet (AIN-93G, AIN-93G/soy, Teklad 2019) for C57BL/6 J mice. Outliers were identified by the ROUT method in GraphPad Prism (*Q* = 1%) and excluded from the analysis.

### 2.4 Cecum sample collection

The cecum was dissected from the abdomen and sliced open. The contents were scraped out with a sterile cell scraper or P1000 pipette tip and transferred to an Eppendorf tube containing 1 mL DNA/RNA Shield Solution (Zymo Research, Irvine, CA) not to exceed 10% maximum weight cecal contents to solution volume. Samples were vortexed, quick frozen, and stored at −80°C.

### 2.5 Microbiome sequencing

Frozen cecum samples were shipped on dry ice to Zymo Research in Irvine, CA. The samples were processed and analyzed with the Zymo Research Microbiome 16S/ITS Amplicon Sequencing Service. DNA was extracted with ZymoBIOMICS® DNA Miniprep Kit and eluted in a 50 μL volume. The sequencing library was prepared with the Quick-16S™ Plus NGS Library Prep Kit utilizing Quick-16S™ Primer Set V3-V4 custom designed primers for coverage of the 16S gene while maintaining high sensitivity (Zymo Research, Irvine, CA). PCR was performed in a real-time PCR machine to control cycles and to limit PCR chimera formation. PCR products were quantified by qPCR fluorescence readings and pooled based on equal molarity. The pooled library was cleaned up with the Select-a-Size DNA Clean & Concentrator™ (Zymo Research, Irvine, CA), and quantified with TapeStation® (Agilent Technologies, Santa Clara, CA) and Invitrogen Qubit 1X DSDNA High Sensitivity Assay Kits® (Thermo Fisher Scientific, Waltham, WA). The positive control for each DNA extraction and each targeted library preparation was the ZymoBIOMICS® Microbial Community Standard (Zymo Research, Irvine, CA). Negative controls included a blank extraction control and a blank library preparation control that assessed the bioburden level in the wet-laboratory process. The library was sequenced with 600 cycles on an Illumina® NextSeq 2000™ with a p1 reagent kit (cat 20,075,294, Illumina, San Diego, CA) and 30% PhiX spike-in.

### 2.6 Microbiome data analysis

Unique amplicon sequences were inferred from raw reads using the Dada2 pipeline (Callahan et al., 2016), with chimeric sequences removed. Taxonomy assignment was made with Uclust from Qiime v.1.9.1 software in conjunction with the reference Zymo Research Database, an internally designed and curated 16S rRNA database. The complete Zymo Research Microbiome Sequencing Service Report containing composition visualization, alpha-diversity, and beta-diversity analyses performed with Qiime v.1.9.1 software (Caporaso et al., 2010) is attached.^1^ Taxonomies with statistically significant differences in abundance between a maximum of four groups were identified by LEfSe using default settings (Segata et al., 2011). Heatmaps, Taxa2SV_deomposer, and PCoA plots were generated with internal scripts (Zymo Research, Irvine, CA).

Absolute Abundance Quantification was determined by quantitative real-time PCR set up with a standard curve made with plasmid DNA containing one copy of the 16S gene and one copy of the fungal ITS2 region prepared in 10-fold serial dilutions. The same primers were used as for the targeted library preparation. A PCR input volume of 2 μL was used to calculate the number of gene copies per microliter in each DNA sample. The number of genome copies per microliter of DNA sample was calculated by dividing the gene copy number by an assumed number of gene copies per genome where the value for 16S copies per genome is 4. The quantity of DNA per microliter of DNA sample was calculated using an assumed genome size of 4.64 × 10^6^ bp, which is the genome size of *Escherichia coli*, for 16S samples. All raw data files are included with the Zymo Research Microbiome Sequencing Service Report (see footnote 1).

Two-way ANOVA analyses of the 16 test cohorts (four genotypes: male and female *Fmr1^KO^* and littermate controls, and four diets: AIN-93G, AIN-93G/soy, Teklad 2019, Purina 5015) at the phylum (p), class (c), order (o), family (f) and genus (g) levels were conducted with GraphPad Prism 10 for macOS, version 10.3.1 software. Where noted in the figure legends, error bars on graphs are shown for diet- and not genotype-specific differences to highlight the most significant findings. Species (s) within kingdom (k)__Bacteria;p__Firmicutes;c__Clostridia;o__Clostridiales;f__Family XIII;*g__Anaerovorax* and f__Ruminococcaceae;*g__NA* were also analyzed as these genera showed increased bacterial expression as a function of AIN-93G/soy compared to the other three diets, which correlates with the FITC-dextran gut permeability data.

## 3 Results

### 3.1 Gut permeability

**Figure 1:**
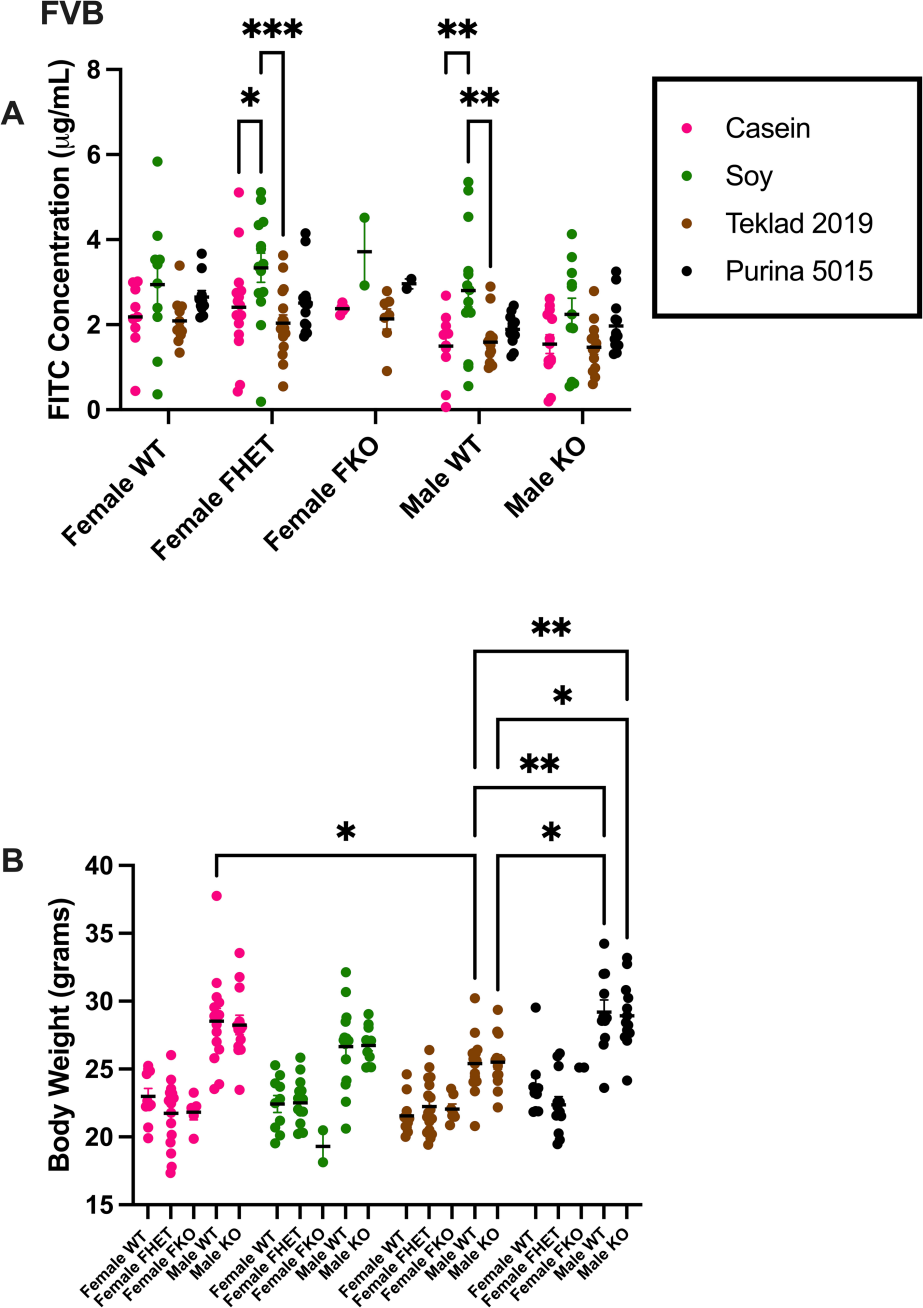
FITC dextran gut permeability and body weight in FVB mice. **(A)** Female WT, *Fmr1^HET^*, *Fmr1^KO^* and male WT and *Fmr1^KO^* mice in a modified FVB background were maintained on AIN-93G, AIN-93G/soy, Teklad 2019 or Purina 5015 diets and at age P70 fasted and oral gavaged with FITC-D4000. Blood plasma levels of FITC (x-axis) are plotted against genotype/diet (y-axis). The AIN-93G cohort (pink) contained WT female (*n* = 9), *Fmr1^HET^* female (*n* = 16), *Fmr1^KO^* female (*n* = 5), WT male (*n* = 10) and *Fmr1^KO^* male (*n* = 13). The AIN-93G/soy cohort (green) contained WT female (*n* = 10), *Fmr1^HET^* (*n* = 14), *Fmr1^KO^* female (*n* = 2), WT male (*n* = 14) and *Fmr1^KO^* male (*n* = 11). The Teklad 2019 cohort (brown) contained WT female (*n* = 11), *Fmr1^HET^* female (*n* = 17), *Fmr1^KO^* female (*n* = 7), WT male (*n* = 14) and *Fmr1^KO^* male (*n* = 13). The Purina 5015 cohort (black) contained WT female (*n* = 10), *Fmr1^HET^* female (*n* = 13), *Fmr1^KO^* female (*n* = 2), WT male (*n* = 11) and *Fmr1^KO^* male (*n* = 13). Littermate mice were tested; only one mouse per genotype was tested per litter. Mice (*n* = 1 WT female on AIN-93G, *n* = 4 WT male on AIN-93G, n = 1 *Fmr1^HET^* female on AIN-93G/soy, *n* = 1 WT male on AIN-93G/soy, and *n* = 1 WT male on Teklad 2019) were excluded as outliers. **(B)** Mice were weighed prior to oral gavage. Body weight in grams (x-axis) is plotted against genotype/diet (y-axis). The AIN-93G cohort (pink) contained WT female (*n* = 10), *Fmr1^HET^* female (n = 16), *Fmr1^KO^* female (*n* = 5), WT male (*n* = 14) and *Fmr1^KO^* male (*n* = 13). The AIN-93G/soy cohort (green) contained WT female (*n* = 10), *Fmr1^HET^* female (*n* = 15), *Fmr1^KO^* female (*n* = 2), WT male (*n* = 15) and *Fmr1^KO^* male (*n* = 11). The Teklad 2019 cohort (brown) contained WT female (*n* = 11), *Fmr1^HET^* female (*n* = 17), *Fmr1^KO^* female (*n* = 7), WT male (*n* = 15) and *Fmr1^KO^* male (*n* = 13). The Purina 5015 cohort (black) contained WT female (*n* = 10), *Fmr1^HET^* female (*n* = 13), *Fmr1^KO^* female (*n* = 2), WT male (*n* = 11) and *Fmr1^KO^* male (*n* = 13). Statistical significance was determined by two-way ANOVA with GraphPad Prism 10, ^*^p < 0.05, ^**^p < 0.01.

Gut permeability and the cecal microbiome were tested in FVB *Fmr1^KO^* mice in response to four mouse diets. The diets included two chows (Purina 5015 and Teklad 2019) and two purified ingredient diets (AIN-93G and AIN-93G/soy). Gut permeability was assessed by FITC-dextran accumulation in blood plasma. There were statistically significant increases in FITC-dextran in *Fmr1^HET^* female and WT male mice fed AIN-93G/soy compared to AIN-93G and Teklad 2019 (Figure 1A). Although not statistically significant, trends for females regardless of genotype included increased gut permeability with AIN-93G/soy > Purina 5015 > AIN-93G > Teklad 2019, and for males AIN-93G/soy > Purina 5015 > AIN-93G and Teklad 2019 (Supplementary Table S1). Trends based on sex show increased gut permeability in females versus males with AIN-93G, Teklad 2019 and Purina 5015. Combining genotypes to increase power showed statistically elevated intestinal permeability with AIN-93G/soy compared to AIN-93G and Teklad 2019 in males and females as well as elevated permeability in females versus males fed AIN-93G, AIN-93G/soy or Teklad 2019 (Supplementary Figure S1). Increased gut permeability with the AIN-93G/soy cohorts was not due to increased FITC-dextran dosage because mice were dosed based on body weight, which was not significantly different with AIN-93G/soy (Figure 1B, Supplementary Figure S2A, and Supplementary Tables S2, S3).

**Figure 2:**
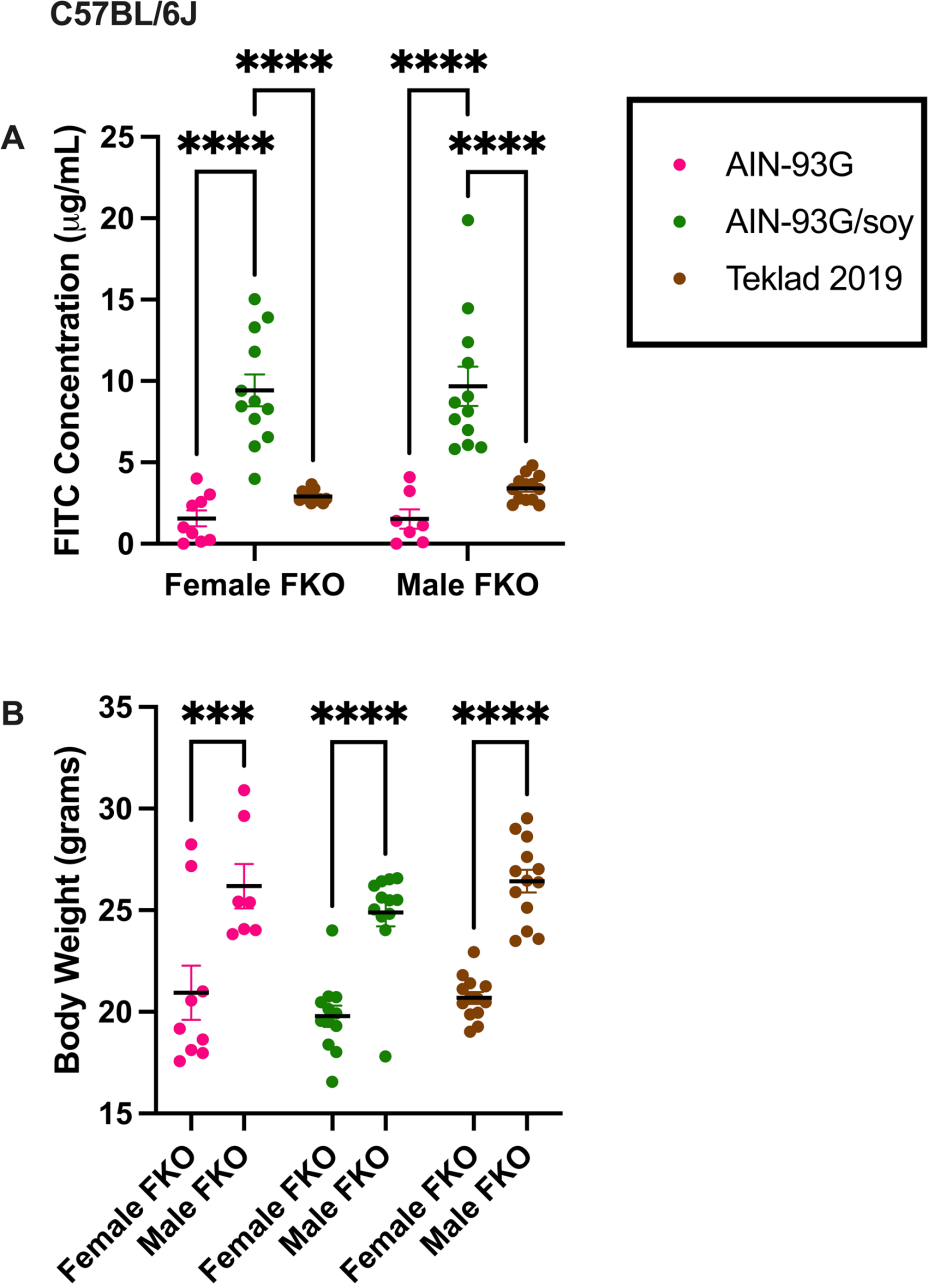
FITC dextran gut permeability and body weight in C57BL/6 J *Fmr1^KO^* mice. **(A)** Female and male *Fmr1^KO^* mice in the C57BL/6 J background were maintained on AIN-93G, AIN-93G/soy or Teklad 2019 for over 10 generations. Mice age P70 were fasted and oral gavaged with FITC-D4000. Blood plasma levels of FITC (x-axis) are plotted against sex/diet (y-axis). The AIN-93G cohort (pink) contained *Fmr1^KO^* female (*n* = 9) and *Fmr1^KO^* male (*n* = 7). The AIN-93G/soy cohort (green) contained *Fmr1^KO^* female (*n* = 12) and *Fmr1^KO^* male (*n* = 12). The Teklad 2019 cohort (brown) contained *Fmr1^KO^* female (*n* = 12) and *Fmr1^KO^* male (*n* = 13). Littermate mice were tested from a minimum of 3 litters. One *Fmr1^KO^* female on Teklad 2019 was excluded as an outlier. **(B)** Mice were weighed prior to oral gavage. Body weight in grams (x-axis) is plotted against sex/diet (y-axis). Statistical significance was determined by two-way ANOVA with GraphPad Prism 10, ^***^p < 0.001, ^****^p < 0.0001.

Gut permeability was also assessed in C57BL/6 J *Fmr1^KO^* mice maintained on AIN-93G, AIN-93G/soy or Teklad 2019 for multiple generations and was elevated at least 2.8-fold with the soy diet in both females and males (Figure 2, Supplementary Table S4). In contrast to our prior study where dams were transferred from Teklad 2019 to AIN-93G or AIN-93G/soy prior to breeding and there was a significant increase in body weight, particularly in *Fmr1^KO^* male offspring, in response to soy (Westmark et al., 2024), here mice were maintained on their respective diets for over 10 generations and there was no significant differences in offspring body weight based on diet (Figure 2, Supplementary Figure S2B, and Supplementary Tables S5, S6). Comparison of average body weight of male *Fmr1*^*K*O^ mice at age 10 weeks of age between this study and the Westmark et al. (2024) study indicated that the multigenerational diet mice weighed less; the 6.1% decrease in body weight did not reach statistical significance with AIN-93G but the 12.5% decrease in body weight with AIN-93G/soy was statistically different between studies (Supplementary Figure S3).

### 3.2 Cecal microbiome

**Figure 3:**
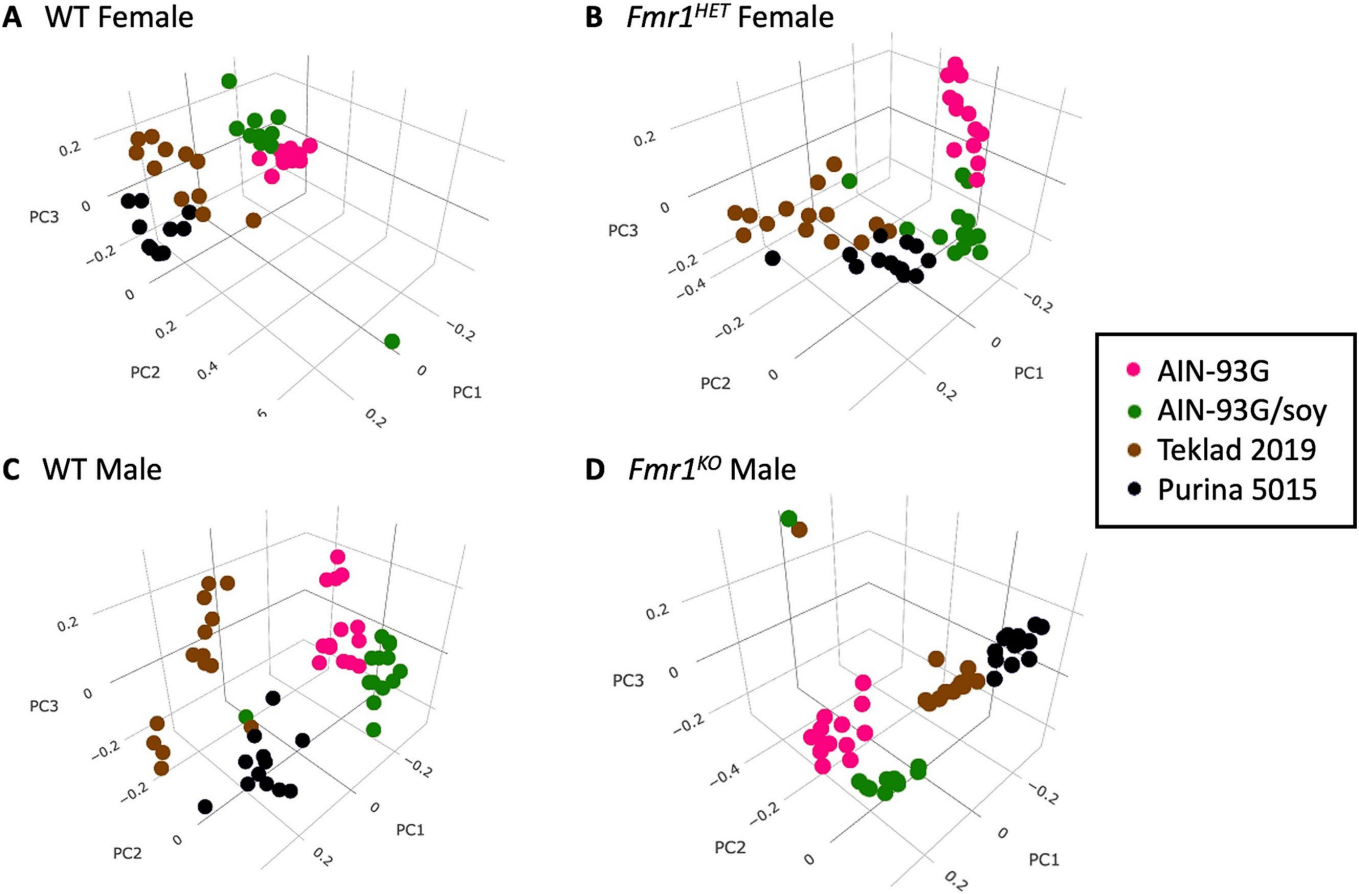
Bray–Curtis dissimilarity visualization of microbial diversity as a function of diet. PCoA plots based on the matrices of paired-wise distances between samples for: **(A)** WT female, **(B)** *Fmr1^HET^* female, **(C)** WT male, and **(D)** *Fmr1^KO^* male mice. Diets are color coded: AIN-93G (pink), AIN-93G/soy (green), Teklad 2019 (brown) and Purina 5015 (black).

Bacterial relative abundance in the cecum in FVB *Fmr1^KO^* and littermate mice was determined by 16S rRNA sequencing followed by alpha diversity, beta diversity, and LEfSe analyses by Zymo Research. Alpha and beta diversity are measures of microbial diversity within and between samples, respectively. The Shannon Index is a measure of alpha diversity. Beta-diversity can be visualized by 3-dimensional principal coordinate analysis (PCoA) plots based on the matrices of paired-wise distances between samples calculated by Bray–Curtis dissimilarity using unique amplicon sequence variants (ASV). LEfSe analysis identifies taxa with statistical (*p* < 0.05) and significant (effect size >2) differences between predefined groups. These analyses were limited to comparison of the four diet groups. There were no significant differences in Shannon Indices (see footnote 1). Beta diversity clustering by PCoA plots differed as a function of diet for each genotype but did not differ as a function of genotype when assessing individual diets (Figure 3, see footnote 1). LEfSe analyses indicated significant differences in numerous taxa as a function of diet (Supplementary Figures S4–S7), but minimal differences as a function of genotype (Supplementary Table S7).

### 3.3 Microbiome abundance as a function of genotype

Two-way ANOVA analyses as a function of diet and genotype were conducted to compare microbiome abundance of the 16 cohorts (four genotypes and four diets). At the phylum level, 6 phyla were identified with the most prevalent being Firmicutes (73%) and Bacteriodetes (23%) (Table 1). Other phyla present were Actinobacteria (2.6%), Verrucomicrobia (0.8%), Proteobacteria (0.7%), and Tenericutes (0.2%). Genotype-specific differences were observed in Verrucomicrobia with increased levels found in *Fmr1^KO^* male mice maintained on AIN-93G compared to WT and *Fmr1^HET^* females. At the class level, eight classes were identified with genotype-specific differences observed in Coriobacteriia, Bacilli, and Verrucomicrobiae, but the only individual comparisons that were significantly different were increased Verrucomicrobiae comparing *Fmr1^KO^* males with WT and *Fmr1^HET^* females fed AIN-93G (Table 2). At the order level, 10 orders were identified with genotype-specific differences observed in Bifidobacteriales, Coriobacteriales, Lactobacillales, and Verrucomicrobiales with individual comparisons indicating statistical differences with increased Coriobacteriales in WT females versus *Fmr1^KO^* males fed Purina 5015 and in *Fmr1^HET^* females versus *Fmr1^KO^* males fed Teklad 2019 as well as increased Verrucomicrobiales in *Fmr1^KO^* males compared to WT and *Fmr1^HET^* females fed AIN-93G (Table 3).

**Table 1:** Statistically significant differences in phyla abundance as a function of genotype and diet.

Phyla	Genotype		Diet		Interaction	
Actinobacteria	*F* (3, 182) = 0.2834	*p* = 0.8373	*F* (3, 182) = 1.954	*p* = 0.1226	*F* (9, 182) = 0.1222	*p* = 0.9991
Bacteroidetes	*F* (3, 184) = 1.525	*p* = 0.2095	*F* (3, 184) = 37.04	*p* < 0.0001	*F* (9, 184) = 1.160	*p* = 0.3231
Firmicutes	*F* (3, 183) = 2.418	*p* = 0.0677	*F* (3, 183) = 5.081	*p* = 0.0021	*F* (9, 183) = 0.3820	*p* = 0.9427
Proeobacteria	*F* (3, 184) = 0.6761	*p* = 0.5677	*F* (3, 184) = 2.389	*p* = 0.0703	*F* (9, 184) = 0.2636	*p* = 0.9834
Tenericutes	*F* (3, 184) = 0.06727	*p* = 0.9772	*F* (3, 184) = 0.4972	*p* = 0.6847	*F* (9, 184) = 0.2441	*p* = 0.9874
Verrucomicrobia	*F* (3, 184) = 3.435	*p* = 0.0181	*F* (3, 184) = 9.115	*p* < 0.0001	*F* (9, 184) = 1.595	*p* = 0.1193

The color highlights statistically significant results.

**Table 2:** Statistically significant differences in class abundance as a function of genotype and diet.

Class	Genotype		Diet		Interaction	
Actinobacteria	*F* (3, 184) = 0.3114	*p* = 0.8172	*F* (3, 184) = 2.067	*p* = 0.1061	*F* (9, 184) = 0.2413	*p* = 0.9879
Coriobacteriia	*F* (3, 184) = 5.739	*p* = 0.0009	*F* (3, 184) = 100.4	*p* < 0.0001	*F* (9, 184) = 2.009	*p* = 0.0404
Bacteroidia	*F* (3, 184) = 1.525	*p* = 0.2096	*F* (3, 184) = 37.04	*p* < 0.0001	*F* (9, 184) = 1.160	P = 0.3231
Bacilli	*F* (3, 184) = 3.365	*p* = 0.0199	*F* (3, 184) = 15.69	*p* < 0.0001	*F* (9, 184) = 0.7796	*p* = 0.6354
Clostridia	*F* (3, 184) = 1.249	*p* = 0.2934	*F* (3, 184) = 6.848	*p* = 0.0002	*F* (9, 184) = 0.2890	*p* = 0.9771
Erysipelotrichia	*F* (3, 184) = 1.441	*p* = 0.2324	*F* (3, 184) = 19.49	*p* < 0.0001	*F* (9, 184) = 0.8467	*p* = 0.5741
Mollicutes	*F* (3, 184) = 0.06727	*p* = 0.9772	F (3, 184) = 0.4972	*p* = 0.6847	*F* (9, 184) = 0.2441	*p* = 0.9874
Verrucomicrobiae	*F* (3, 184) = 3.435	*p* = 0.0181	*F* (3, 184) = 9.115	*p* < 0.0001	*F* (9, 184) = 1.595	*p* = 0.1193

The color highlights statistically significant results.

**Table 3:** Statistically significant differences in order abundance as a function of genotype and diet.

Order	Genotype		Diet	Interaction		
Bifidobacteriales	*F* (3, 184) = 3.436	*p* = 0.0181	*F* (3, 184) = 3.317	*p* = 0.0211	*F* (9, 184) = 0.8035	*p* = 0.6135
Coriobacteriales	*F* (3, 184) = 5.739	*p* = 0.0009	*F* (3, 184) = 100.4	*p* < 0.0001	*F* (9, 184) = 2.009	*p* = 0.0404
Bacteroidales	*F* (3, 184) = 1.525	*p* = 0.2096	*F* (3, 184) = 37.04	*p* < 0.0001	*F* (9, 184) = 1.160	*p* = 0.3231
Bacillales	*F* (3, 184) = 0.08648	*p* = 0.9674	*F* (3, 184) = 0.5990	*p* = 0.6165	*F* (9, 184) = 1.319	*p* = 0.2294
Lactobacillales	*F* (3, 184) = 3.250	*p* = 0.0231	*F* (3, 184) = 15.50	*p* < 0.0001	*F* (9, 184) = 0.9222	*p* = 0.5071
Clostridiales	*F* (3, 184) = 1.249	*p* = 0.2934	*F* (3, 184) = 6.848	*p* = 0.0002	*F* (9, 184) = 0.2890	*p* = 0.9771
Erysipelotrichales	*F* (3, 184) = 1.441	*p* = 0.2324	*F* (3, 184) = 19.49	*p* < 0.0001	*F* (9, 184) = 0.8467	*p* = 0.5741
Anaeroplasmatales	*F* (3, 184) = 0.1602	*p* = 0.9230	*F* (3, 184) = 1.022	*p* = 0.3841	*F* (9, 184) = 0.2717	*p* = 0.9815
NA	*F* (3, 184) = 1.904	*p* = 0.1305	*F* (3, 184) = 12.87	*p* < 0.0001	*F* (9, 184) = 0.5842	*p* = 0.8091
Verrucomicrobiales	*F* (3, 184) = 3.435	*p* = 0.0181	*F* (3, 184) = 9.115	*p* < 0.0001	*F* (9, 184) = 1.595	*p* = 0.1193

The color highlights statistically significant results.

At the family level, 19 families were identified with genotype-specific differences observed in *Bifidobacteriaceae* (no significant individual comparisons), *Coriobacteriaceae* (increased in WT females versus *Fmr1^KO^* males fed Purina 5015 and in *Fmr1^HET^* females versus *Fmr1^KO^* males fed Teklad 2019), *o__Bacteroidales;f__NA* (decreased in WT females versus WT males fed Teklad 2019), *Clostridiaceae* (increased in *Fmr1^KO^* males versus WT and *Fmr1^HET^* females fed Purina 5015), *Lachnospiraceae* (no significant individual comparisons), and *Verrucomicrobiaceae* (increased in *Fmr1^KO^* males compared to WT and *Fmr1^HET^* females fed AIN-93G) (Table 4). At the genus level, 31 genera were identified with genotype-specific differences observed in Bifidobacterium (no individual differences), *Enterorhabdus* (increased in WT females versus *Fmr1^KO^* males fed Purina 5015 and in *Fmr1^HET^* females versus *Fmr1^KO^* males fed Teklad 2019), *o__Bacteroidales;f__NA;g__NA* (decreased in WT females versus WT males fed Teklad 2019), *Clostridium* (increased in *Fmr1^KO^* males versus WT and *Fmr1^HET^* females fed Purina 5015), *Blautia* (no significant individual comparisons) and *Akkermansia* (increased in *Fmr1^KO^* males compared to WT and *Fmr1^HET^* females fed AIN-93G) (Table 5). Overall, within sexes on the same diet, there were no statistically significant WT versus *Fmr1* genotype-specific differences down to the genus level.

**Table 4:** Statistically significant differences in family abundance as a function of genotype and diet.

Family	Genotype		Diet		Interaction	
Bifidobacteriaceae	*F* (3, 184) = 3.436	*p* = 0.0181	*F* (3, 184) = 3.317	*p* = 0.0211	*F* (9, 184) = 0.8035	*p* = 0.6135
Coriobacteriaceae	*F* (3, 184) = 5.739	*p* = 0.0009	*F* (3, 184) = 100.4	*p* < 0.0001	*F* (9, 184) = 2.009	*p* = 0.0404
NA	*F* (3, 184) = 2.898	*p* = 0.0364	*F* (3, 184) = 106.5	*p* < 0.0001	*F* (9, 184) = 1.314	*p* = 0.2320
Porphyromonadaceae	*F* (3, 184) = 0.7234	*p* = 0.5392	*F* (3, 184) = 88.17	*p* < 0.0001	*F* (9, 184) = 1.198	*p* = 0.2988
Rikenellaceae	*F* (3, 184) = 0.6682	*p* = 0.5726	*F* (3, 184) = 38.45	*p* < 0.0001	*F* (9, 184) = 0.9022	*p* = 0.5246
Staphylococcaceae	*F* (3, 184) = 0.4082	*p* = 0.7473	*F* (3, 184) = 1.169	*p* = 0.3231	*F* (9, 184) = 1.206	*p* = 0.2935
Lactobacillaceae	*F* (3, 184) = 1.983	*p* = 0.1180	*F* (3, 184) = 19.03	*p* < 0.0001	*F* (9, 184) = 0.8102	*p* = 0.6074
Streptococcaceae	*F* (3, 184) = 1.182	*p* = 0.3178	*F* (3, 184) = 1.614	*p* = 0.1876	*F* (9, 184) = 1.407	*p* = 0.1879
Christensenellaceae	*F* (3, 184) = 1.469	*p* = 0.2244	*F* (3, 184) = 103.0	*p* < 0.0001	*F* (9, 184) = 0.8599	*p* = 0.5622
Clostridiaceae	*F* (3, 184) = 5.712	*p* = 0.0009	*F* (3, 184) = 9.987	*p* < 0.0001	*F* (9, 184) = 1.676	*p* = 0.0976
Family XIII	*F* (3, 184) = 0.3471	*p* = 0.7913	*F* (3, 184) = 8.023	*p* < 0.0001	*F* (9, 184) = 0.6182	*p* = 0.7806
Lachnospiraceae	*F* (3, 184) = 4.296	*p* = 0.0059	*F* (3, 184) = 3.693	*p* = 0.0129	*F* (9, 184) = 0.3742	*p* = 0.9463
NA	*F* (3, 184) = 1.082	*p* = 0.3580	*F* (3, 184) = 30.83	*p* < 0.0001	*F* (9, 184) = 0.3080	*p* = 0.9716
Peptococcaceae	*F* (3, 184) = 0.1979	*p* = 0.8977	*F* (3, 184) = 1.677	*p* = 0.1735	*F* (9, 184) = 0.4366	*p* = 0.9140
Ruminococcaceae	*F* (3, 184) = 0.6949	*p* = 0.5562	*F* (3, 184) = 47.83	*p* < 0.0001	*F* (9, 184) = 0.4361	*p* = 0.9142
Erysipelotrichaceae	*F* (3, 184) = 1.441	*p* = 0.2324	*F* (3, 184) = 19.49	*p* < 0.0001	*F* (9, 184) = 0.8467	*p* = 0.5741
Anaeroplasmataceae	*F* (3, 184) = 0.1602	*p* = 0.9230	*F* (3, 184) = 1.022	*p* = 0.3841	*F* (9, 184) = 0.2717	p = 0.9815
NA	*F* (3, 184) = 1.904	*p* = 0.1305	*F* (3, 184) = 12.87	*p* < 0.0001	*F* (9, 184) = 0.5842	*p* = 0.8091
Verrucomicrobiaceae	*F* (3, 184) = 3.435	*p* = 0.0181	*F* (3, 184) = 9.115	*p* < 0.0001	*F* (9, 184) = 1.595	*p* = 0.1193

The color highlights statistically significant results.

**Table 5:** Statistically significant differences in genus abundance as a function of genotype and diet.

Genus	Genotype		Diet		Interaction	
*Bifidobacterium*	*F* (3, 184) = 3.436	*p* = 0.0181	*F* (3, 184) = 3.317	*p* = 0.0211	*F* (9, 184) = 0.8035	*p* = 0.6135
*Enterorhabdus*	*F* (3, 184) = 5.871	*p* = 0.0008	*F* (3, 184) = 91.51	*p* < 0.0001	*F* (9, 184) = 2.131	*p* = 0.0290
*NA*	*F* (3, 184) = 2.898	*p* = 0.0364	*F* (3, 184) = 106.5	*p* < 0.0001	*F* (9, 184) = 1.314	*p* = 0.2320
*Parabacteroides*	*F* (3, 184) = 0.7234	*p* = 0.5392	*F* (3, 184) = 88.17	*p* < 0.0001	*F* (9, 184) = 1.198	*p* = 0.2988
*Alistipes*	*F* (3, 184) = 0.6682	*p* = 0.5726	*F* (3, 184) = 38.45	*p* < 0.0001	*F* (9, 184) = 0.9022	*p* = 0.5246
*Staphylococcus*	*F* (3, 184) = 0.4082	*p* = 0.7473	*F* (3, 184) = 1.169	*p* = 0.3231	*F* (9, 184) = 1.206	*p* = 0.2935
*Lactobacillus*	*F* (3, 184) = 1.983	*p* = 0.1180	*F* (3, 184) = 19.03	*p* < 0.0001	*F* (9, 184) = 0.8102	*p* = 0.6074
*Lactococcus*	*F* (3, 184) = 1.188	*p* = 0.3157	*F* (3, 184) = 1.742	*p* = 0.1599	*F* (9, 184) = 1.411	*p* = 0.1862
*NA*	*F* (3, 184) = 1.469	*p* = 0.2244	*F* (3, 184) = 103.0	*p* < 0.0001	*F* (9, 184) = 0.8599	*p* = 0.5622
*Clostridium*	*F* (3, 184) = 5.712	*p* = 0.0009	*F* (3, 184) = 9.987	*p* < 0.0001	*F* (9, 184) = 1.676	*p* = 0.0976
*Anaerovorax*	*F* (3, 184) = 0.3345	*p* = 0.8004	*F* (3, 184) = 57.67	*p* < 0.0001	*F* (9, 184) = 0.6644	*p* = 0.7404
*NA*	*F* (3, 184) = 0.3627	*p* = 0.7800	*F* (3, 184) = 10.65	*p* < 0.0001	*F* (9, 184) = 0.7622	*p* = 0.6515
*Acetatifactor*	*F* (3, 184) = 0.3387	*p* = 0.7974	*F* (3, 184) = 20.99	*p* < 0.0001	*F* (9, 184) = 0.1443	*p* = 0.9983
*Blautia*	*F* (3, 184) = 3.269	*p* = 0.0225	*F* (3, 184) = 134.1	*p* < 0.0001	*F* (9, 184) = 0.8466	*p* = 0.5742
*Lachnoclostridium*	*F* (3, 184) = 0.5544	*p* = 0.6458	*F* (3, 184) = 21.84	*p* < 0.0001	*F* (9, 184) = 1.211	*p* = 0.2904
*NA*	*F* (3, 184) = 2.581	*p* = 0.0549	*F* (3, 184) = 35.35	*p* < 0.0001	*F* (9, 184) = 0.6117	*p* = 0.7862
*Robinsoniella*	*F* (3, 184) = 0.5960	*p* = 0.6184	*F* (3, 184) = 19.01	*p* < 0.0001	*F* (9, 184) = 1.352	*p* = 0.2130
*Roseburia*	*F* (3, 184) = 0.7191	*p* = 0.5417	*F* (3, 184) = 12.56	*p* < 0.0001	*F* (9, 184) = 0.8921	*p* = 0.5335
*Tyzzerella*	*F* (3, 184) = 1.752	*p* = 0.1580	*F* (3, 184) = 126.7	*p* < 0.0001	*F* (9, 184) = 0.7223	*p* = 0.6881
*NA*	*F* (3, 184) = 1.082	p = 0.3580	*F* (3, 184) = 30.83	*p* < 0.0001	*F* (9, 184) = 0.3080	*p* = 0.9716
*NA*	*F* (3, 184) = 0.1626	*p* = 0.9214	*F* (3, 184) = 4.443	*p* = 0.0048	*F* (9, 184) = 0.2402	*p* = 0.9881
*Anaerotruncus*	*F* (3, 184) = 0.7873	*p* = 0.5024	*F* (3, 184) = 21.33	*p* < 0.0001	*F* (9, 184) = 0.3809	*p* = 0.9432
*NA*	*F* (3, 184) = 0.8730	*p* = 0.4561	*F* (3, 184) = 49.22	*p* < 0.0001	*F* (9, 184) = 0.8765	*p* = 0.5473
*Oscillibacter*	*F* (3, 184) = 1.040	*p* = 0.3762	*F* (3, 184) = 11.36	*p* < 0.0001	*F* (9, 184) = 0.3834	*p* = 0.9420
*Ruminiclostridium*	*F* (3, 184) = 0.01908	*p* = 0.9964	*F* (3, 184) = 6.613	*p* = 0.0003	*F* (9, 184) = 0.6725	*p* = 0.7332
*Ruminococcus*	*F* (3, 184) = 1.013	*p* = 0.3883	*F* (3, 184) = 32.03	*p* < 0.0001	*F* (9, 184) = 0.2032	*p* = 0.9936
*NA*	*F* (3, 184) = 0.4985	*p* = 0.6838	*F* (3, 184) = 17.44	*p* < 0.0001	*F* (9, 184) = 0.7315	*p* = 0.6796
*Turicibacter*	*F* (3, 184) = 1.689	*p* = 0.1710	*F* (3, 184) = 39.62	*p* < 0.0001	*F* (9, 184) = 0.6622	*p* = 0.7423
*Anaeroplasma*	*F* (3, 184) = 0.1602	*p* = 0.9230	*F* (3, 184) = 1.022	*p* = 0.3841	*F* (9, 184) = 0.2717	*p* = 0.9815
*NA*	*F* (3, 184) = 1.904	*p* = 0.1305	*F* (3, 184) = 12.87	*p* < 0.0001	*F* (9, 184) = 0.5842	*p* = 0.8091
*Akkermansia*	*F* (3, 184) = 3.435	*p* = 0.0181	*F* (3, 184) = 9.115	*p* < 0.0001	*F* (9, 184) = 1.595	*p* = 0.1193

The color highlights statistically significant results.

### 3.4 Microbiome abundance as a function of diet

**Figure 4:**
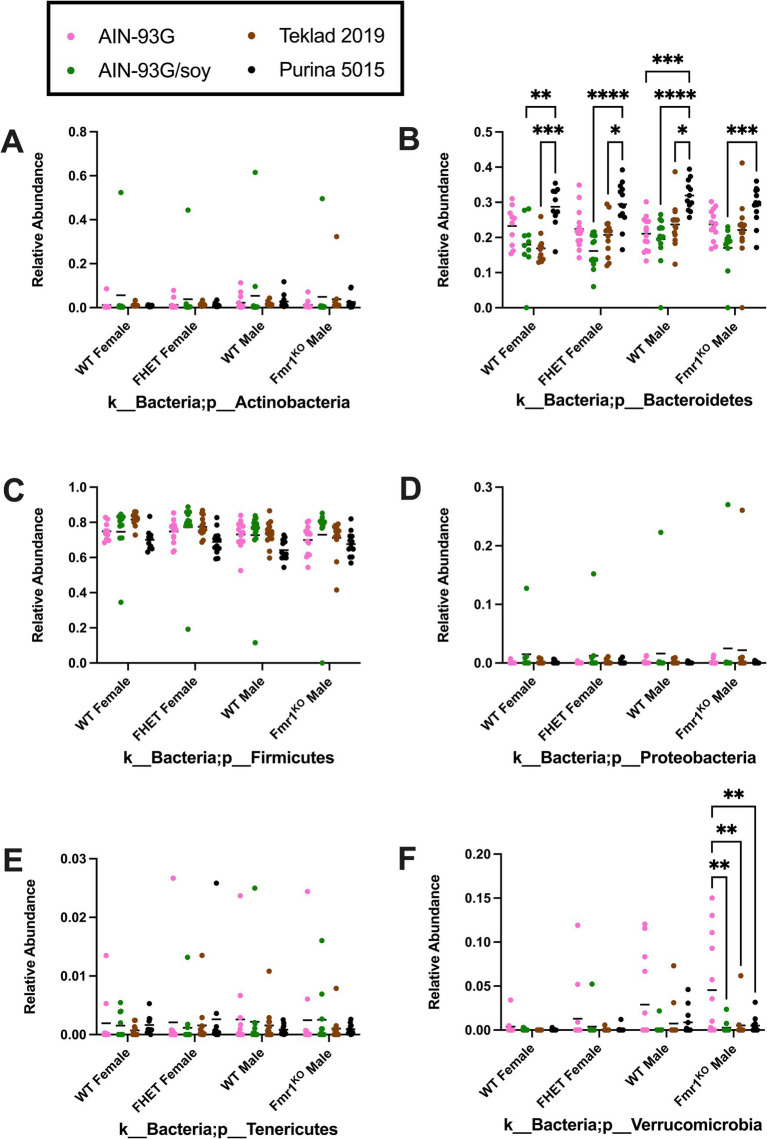
Microbiome relative abundance at the phylum level as a function of genotype and diet. Relative abundance of positive reads out of the total number of reads after filtering (x-axis) is plotted versus genotype/diet for: **(A)** Actinobacteria, **(B)** Bacteriodetes, **(C)** Firmicutes, **(D)** Proteobacteria, **(E)** Tenericutes, and **(F)** Verrucomicrobia. Diets are color coded AIN-93G (pink), AIN-93G/soy (green), Teklad 2–19 Teklad 2019 (brown), and Purina 5015 (black). Statistical significance was determined by two-way ANOVA with GraphPad Prism 10, ^*^p < 0.05, ^**^p < 0.01, ^***^p < 0.001, ^****^p < 0.0001. Error bars comparing diets for each genotype are shown on the graphs. Abbreviations for titles on the x-axis: k, kingdom; p, phylum.

**Figure 5:**
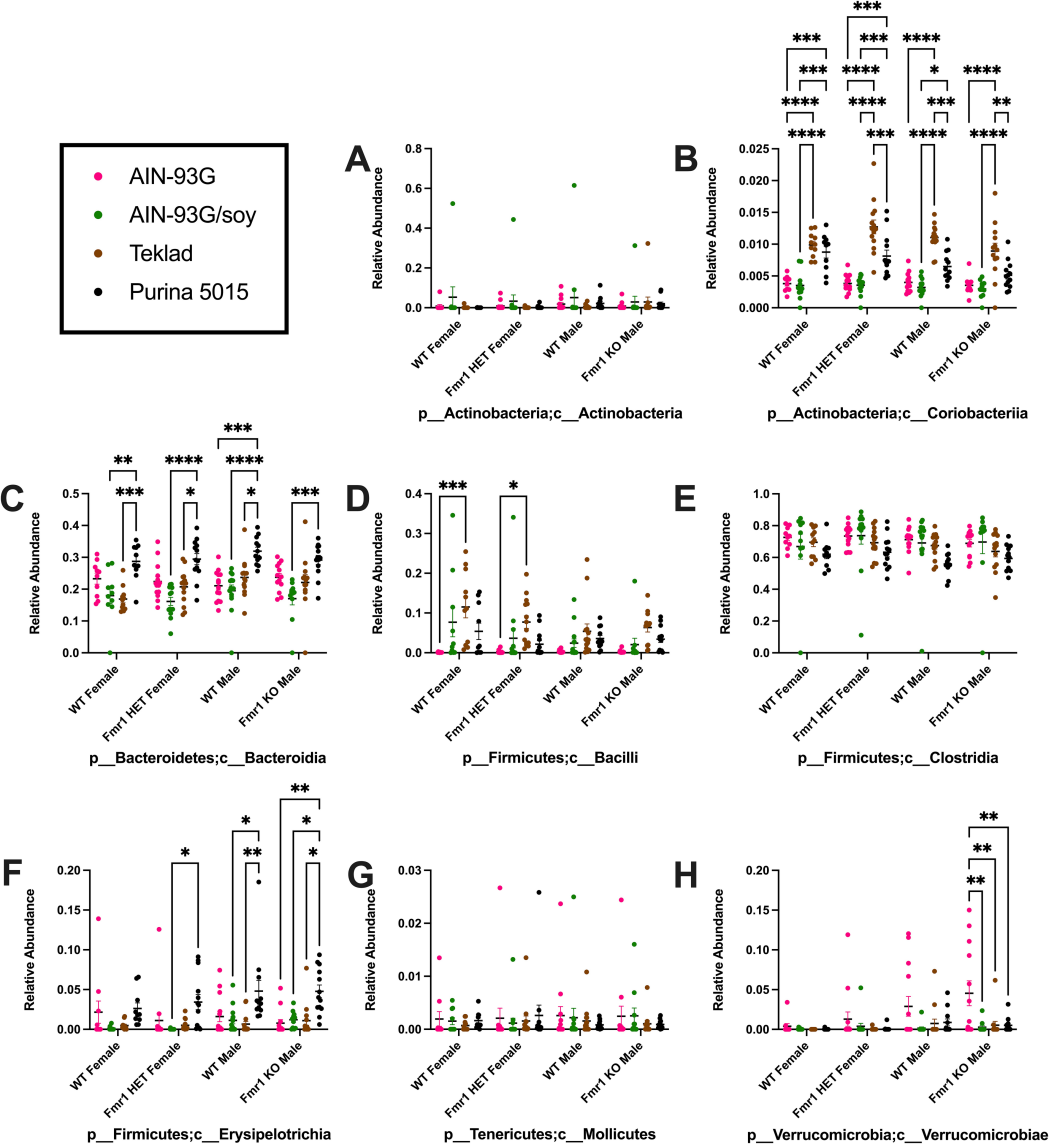
Microbiome relative abundance at the class level as a function of genotype and diet. Relative abundance of positive reads out of the total number of reads after filtering (x-axis) is plotted versus genotype/diet for: **(A)** Actinobacteria, **(B)** Coriobacteriia, **(C)** Bacteroidia, **(D)** Bacilli, **(E)** Clostridia, **(F)** Erysipelotrichia, **(G)** Mollicutes, and **(H)** Verrucomicrobiae. Diets are color coded AIN-93G (pink), AIN-93G/soy (green), Teklad 2019 (brown), and Purina 5015 (black). Statistical significance was determined by two-way ANOVA with GraphPad Prism 10, ^*^p < 0.05, ^**^p < 0.01, ^***^p < 0.001, ^****^p < 0.0001. Error bars comparing diets for each genotype are shown on the graphs. Abbreviations for titles on the x-axis: p, phylum; c, class.

Diet had a larger effect on bacterial abundance than *Fmr1* genotype. At the phylum level, diet-specific differences by two-way ANOVA were observed in Bacteriodetes (increased with Purina 5015 compared to one or more other diets for all genotypes), Firmicutes (no significant individual differences) and Verrucomicrobia (increased with AIN-93G compared to the other three diets in *Fmr1^KO^* males) (Figure 4, Table 1). At the class level, diet-specific differences were observed in Coriobacteriia (increased with Teklad 2019 compared to the other three diets in WT and *Fmr1^KO^* males and in *Fmr1^HET^* females, increased with Teklad 2019 compared to both purified ingredient dies in WT females, increased with Purina 5015 versus both purified ingredient diets in WT and *Fmr1^HET^* females, and increased with Purina 5015 versus AIN-93G/soy in WT males), Bacteroidia (increased with Purina 5015 and one or more of the other diets dependent on genotype), Bacilli (increased with Teklad 2019 compared to AIN-93G in WT and *Fmr1^HET^* females), Clostridia (no individual differences), Erysipelotrichia (increased with Purina 5015 versus one or more of the other diets in *Fmr1^HET^* females and WT and *Fmr1^KO^* males) and Verrucomicrobiae (increased with AIN-93G compared to the other three diets in *Fmr1^KO^* males) (Figure 5, Table 2).

**Figure 6:**
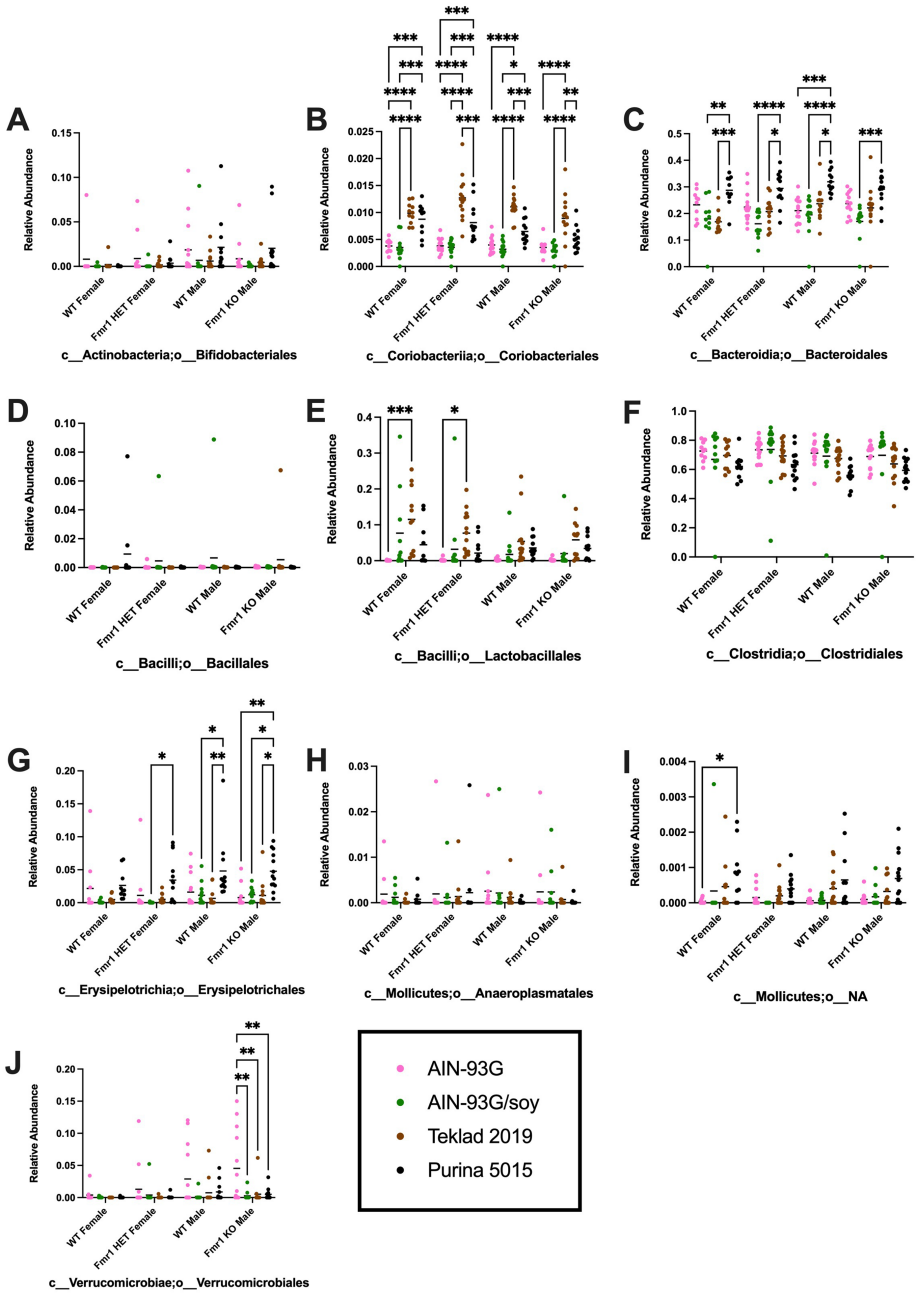
Microbiome relative abundance at the order level as a function of genotype and diet. Relative abundance of positive reads out of the total number of reads after filtering (x-axis) is plotted versus genotype/diet for: **(A)** Bifidobacteriales, **(B)** Coriobacteriales, **(C)** Bacteroidales, **(D)** Bacillales, **(E)** Lactobacillales, **(F)** Clostridiales, **(G)** Erysipelotrichales, **(H)** Anaeroplasmatales, **(I)** Mollicutes;o*__*NA, and **(J)** Verrucomicrobiales. Diets are color coded AIN-93G (pink), AIN-93G/soy (green), Teklad 2019 (brown), and Purina 5015 (black). Statistical significance was determined by two-way ANOVA with GraphPad Prism 10, ^*^p < 0.05, ^**^p < 0.01, ^***^p < 0.001, ^****^p < 0.0001. Error bars comparing diets for each genotype are shown on the graphs. Abbreviations for titles on the x-axis: c, class; o, order.

At the order level, diet-specific differences were observed in Bifidobacteriales (no significant individual differences), Coriobacteriales (increased with Teklad 2019 compared to the other three diets in WT and *Fmr1^KO^* males and in *Fmr1^HET^* females, increased with Teklad 2019 compared to both purified ingredient dies in WT females, increased with Purina 5015 versus both purified ingredient diets in WT and *Fmr1^HET^* females, and increased with Purina 5015 versus AIN-93G/soy in WT males), Bacteroidales (increased with Purina 5015 versus all other diets in WT males, versus AIN-93G/soy and Teklad 2019 in WT and *Fmr1^HET^* females, and versus AIN-93G/soy in *Fmr1^KO^* males), Lactobacillales (increased with Teklad 2019 versus AIN-93G in WT and *Fmr1^HET^* females), Clostridiales (no significant individual differences), Erysipelotrichales (increased with Purina 5015 versus one or more of the other diets in *Fmr1^HET^* females and WT and *Fmr1^KO^* males), Mollicutes;o__*NA* (increased with Purina 5015 versus AIN-93G in WT females) and Verrucomicrobiales (between AIN-93G and the other three diets in *Fmr1^KO^* males) (Figure 6, Table 3).

**Figure 7:**
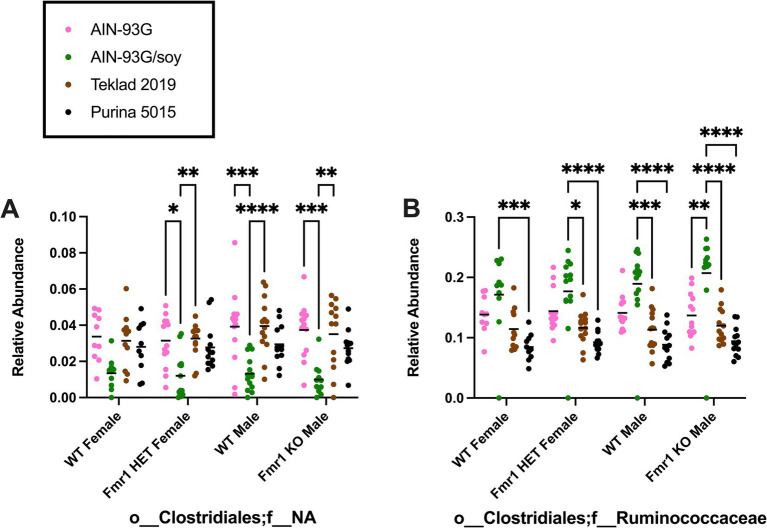
Microbiome relative abundance at the family level as a function of genotype and diet. Relative abundance of positive reads out of the total number of reads after filtering (x-axis) is plotted versus genotype/diet for: **(A)** Clostridiales;f*__*NA, and **(B)** Clostridiales;f*__*Ruminococcaceae. Diets are color coded AIN-93G (pink), AIN-93G/soy (green), Teklad 2019 (brown), and Purina 5015 (black). Statistical significance was determined by two-way ANOVA with GraphPad Prism 10, ^*^p < 0.05, ^**^p < 0.01, ^***^p < 0.001, ^****^p < 0.0001. Error bars comparing diets for each genotype are shown on the graphs. Abbreviations for titles on the x-axis: o, order; f, family.

At the family level, diet-specific differences were observed in Bifidobacteriaceae (no significant individual comparisons), Coriobacteriaceae (increased with Teklad 2019 compared to the other three diets in WT and *Fmr1^KO^* males and in *Fmr1^HET^* females, increased with Teklad 2019 compared to both purified ingredient dies in WT females, increased with Purina 5015 versus both purified ingredient diets in WT and *Fmr1^HET^* females, and increased with Purina 5015 versus AIN-93G/soy in WT males), o__Bacteroidales;f__NA (increased with Purina 5015 versus all other diets in all genotypes and increased with Teklad 2019 versus both purified ingredient diets in WT males), Porphyromonadaceae (increased with AIN-93G versus the other three diets in all genotypes), Rikenellaceae (increased with AIN-93G versus both chows in *Fmr1^HET^* females and WT and *Fmr1^KO^* males and versus Purina 5015 in WT females, and increased with AIN-93G/soy versus both chows in WT males), Lactobacillaceae (increased with Teklad 2019 versus AIN-93G in WT and *Fmr1^HET^* females), Christensenellaceae (increased with AIN-93G versus both chows in all genotypes, increased with AIN-93G/soy versus both chows in WT and *Fmr1*^*HE*T^ females, and increased with AIN-93G/soy versus Purina 5015 in WT and *Fmr1^KO^* males), Clostridiaceae (increased with Purina 5015 versus AIN-93G/soy and Teklad 2019 in *Fmr1^KO^* male), Family XIII (no significant individual comparisons), Lachnospiraceae (no significant individual comparisons), o__Clostridiales;f__NA (decreased with AIN-93G/soy versus AIN-93G and Teklad 2019 in *Fmr1^HET^* females and WT and *Fmr1^KO^* males), Ruminococcacea (increased with AIN-93G/soy versus one or more of the other diets), Erysipelotrichaceae (increased with Purina 5015 versus one or more of the other diets in *Fmr1^HET^* female and WT and *Fmr1^KO^* male mice), Mollicutes;o__NA;f__NA (increased with Purina 5015 versus AIN-93G in WT females) and Verrucomicrobiaceae (increased with AIN-93G versus the other three diets in *Fmr1^KO^* males) (Table 4, Supplementary Figure S8). Of interest, altered levels of o__Clostridiales;f__NA and Ruminococcacea negatively and positively correlate, respectively, with increased gut permeability in response to AIN-93G/soy (Figure 7).

**Figure 8:**
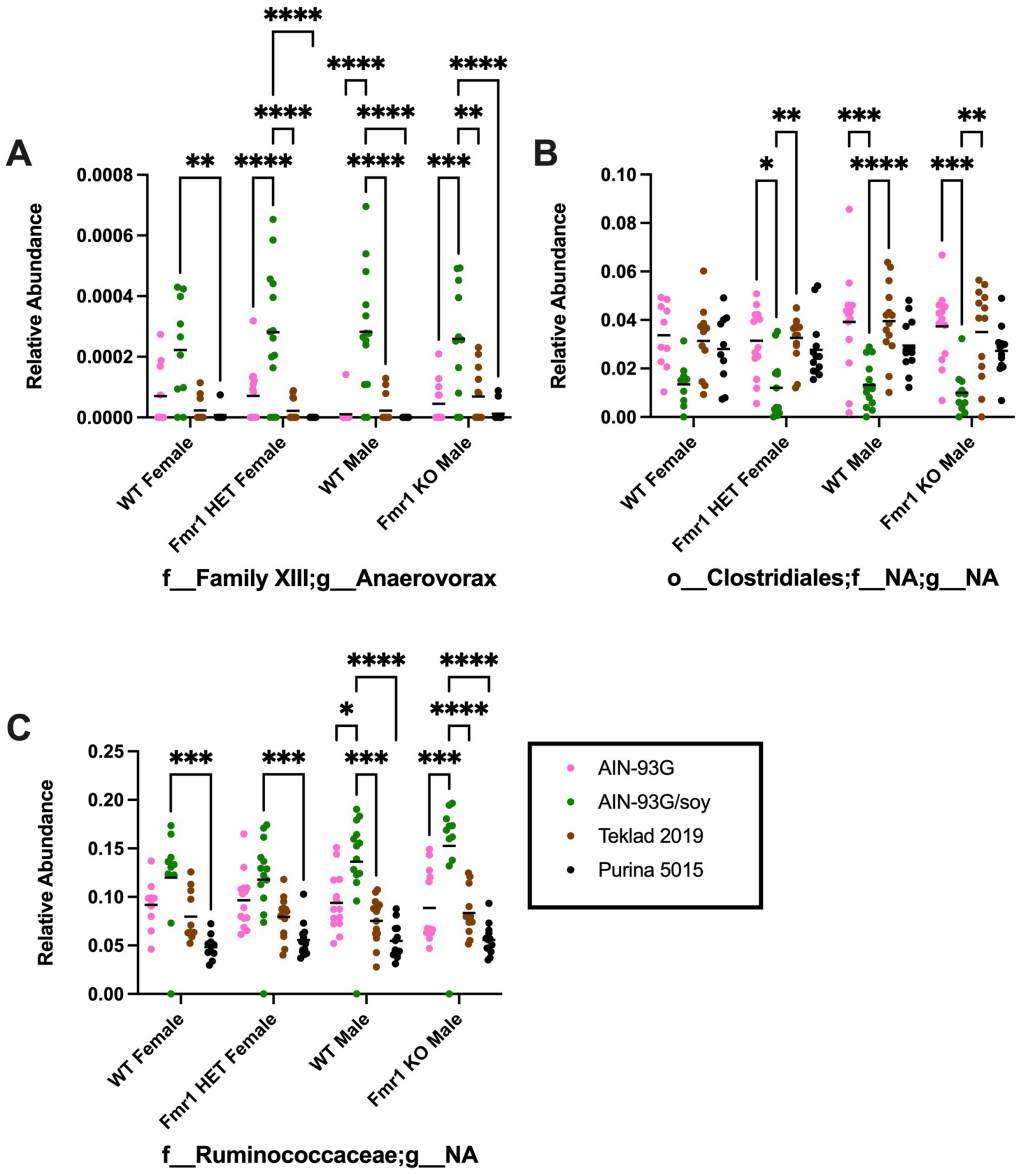
Microbiome relative abundance at the genus level as a function of genotype and diet. Relative abundance of positive reads out of the total number of reads after filtering (x-axis) is plotted versus genotype/diet for: **(A)** Anaerovorax, **(B)** Clostridiales;f*__*NA;g*__*NA, and **(C)** Clostridiales;f*__*Ruminococcaceae;g*__*NA. Diets are color coded AIN-93G (pink), AIN-93G/soy (green), Teklad 2019 (brown), and Purina 5015 (black). Statistical significance was determined by two-way ANOVA with GraphPad Prism 10, ^*^p < 0.05, ^**^p < 0.01, ^***^p < 0.001, ^****^p < 0.0001. Error bars comparing diets for each genotype are shown on the graphs. Abbreviations for titles on the x-axis: f, family; g, genus.

At the genus level, diet-specific differences were observed in *Bifidobacterium* (no significant individual comparisons), *Enterorhabdus* (increased with Teklad 2019 compared to the other three diets in WT and *Fmr1^KO^* males and in *Fmr1^HET^* females, increased with Teklad 2019 compared to both purified ingredient dies in WT females, increased with Purina 5015 versus both purified ingredient diets in WT and *Fmr1^HET^* females, and increased with Purina 5015 versus AIN-93G/soy in WT males), *Bacteroidales;f__NA;g__NA* (increased with Purina 5015 and the other three diets for all genotypes and increased with Teklad 2019 versus both purified ingredient diets in WT males), *Parabacteroides* (increased with AIN-93G versus the other three diets in all genotypes), *Alistipes* (increased with AIN-93G versus both chows in *Fmr1^HET^* females and WT and *Fmr1^KO^* males and versus Purina 5015 in WT females, and increased with AIN-93G/soy versus both chows in WT males), *Lactobacillus* (increased with Teklad 2019 versus AIN-93G in WT and *Fmr1^HET^* females), Christensenellaceae;*g*__*NA* (increased with AIN-93G versus both chows in all genotypes, increased with AIN-93G/soy versus both chows in WT and *Fmr1*^*HE*T^ females, and increased with AIN-93G/soy versus Purina 5015 in WT and *Fmr1^KO^* males), *Clostridium* (increased with Purina 5015 versus AIN-93G/soy and Teklad 2019 in *Fmr1^KO^* male), *Anaerovorax* (increased with AIN-93G/soy versus all other diets in *Fmr1^HET^* females and WT and *Fmr1^KO^* males and versus Purina 5015 in WT females), *Family XIII;g__NA* (increased with Teklad 2019 versus AIN-93G/soy in WT males), *Acetatifactor* (increased with AIN-93G versus Teklad 2019 in *Fmr1^HET^* females and versus both chows in WT males), *Blautia* (increased with AIN-93G versus the other 3 diets in all genotypes and with AIN-93G/soy versus Purina 5015 in *Fmr1^HET^* females), *Lachnoclostridium* (increased with AIN-93G versus Teklad 2019 in WT males and versus both chows in *Fmr1^KO^* males), *Lachnospiraceae;g__NA* (increased with Teklad 2019 versus AIN-93G for all genotypes and versus AIN-93G/soy in WT and *Fmr1^HET^* females and WT males), *Robinsoniella* (increased with Purina 5015 versus one or more diets in *Fmr1^HET^* females and WT and *Fmr1^KO^* males), *Roseburia* (increased with AIN-93G/soy versus Teklad 2019 in *Fmr1^HET^* females), *Tyzzerella* (increased with both purified ingredients versus both chows as well as with AIN-93G versus AIN-93G/soy in WT and *Fmr1^KO^* males), *Clostridiales;f__NA;g__NA* (decreased with AIN-93G/soy versus AIN-93G and Teklad 2019 in *Fmr1^HET^* females and WT and *Fmr1^KO^* males), *Peptococcaceae;g__NA* (no significant individual comparisons), *Anaerotruncus* (increased with AIN-93G versus Purina 5015 in WT and *Fmr1^HET^* females and WT males), *Ruminococcaceae;g__NA* (increased with AIN-93G/soy versus all other diets in WT and *Fmr1^KO^*males and versus Purina 5015 in WT and *Fmr1^HET^* females), *Oscillibacter* (no significant individual comparisons), *Ruminiclostridium* (no significant individual comparisons), *Ruminococcus* (increased with AIN-93G/soy versus both chows for all genotypes and with AIN-93G versus Teklad 2019 in *Fmr1^KO^* males), *Erysipelotrichaceae;g__NA* (increased with Purina 5015 versus both purified ingredient diets in WT females), Turicibacter (increased with Purina 5015 versus two or three of the other diets for all genotypes), *Mollicutes;o__NA;f__NA;g__NA* (increased with Purina 5015 versus AIN-93G in WT females), and *Akkermansia* (increased with AIN-93G versus the other three diets in *Fmr1^KO^* males) (Table 5, Supplementary Figure S9). Of interest, altered levels of f__Family XIII;g__Anaerovorax and f__Ruminococcaceae;g__NA correlate positively and o__Clostridiales;f__NA;g__NA correlates negatively with increased gut permeability in response to AIN-93G/soy (Figure 8).

### 3.5 Species level differences as a function of genotype and diet

**Figure 9:**
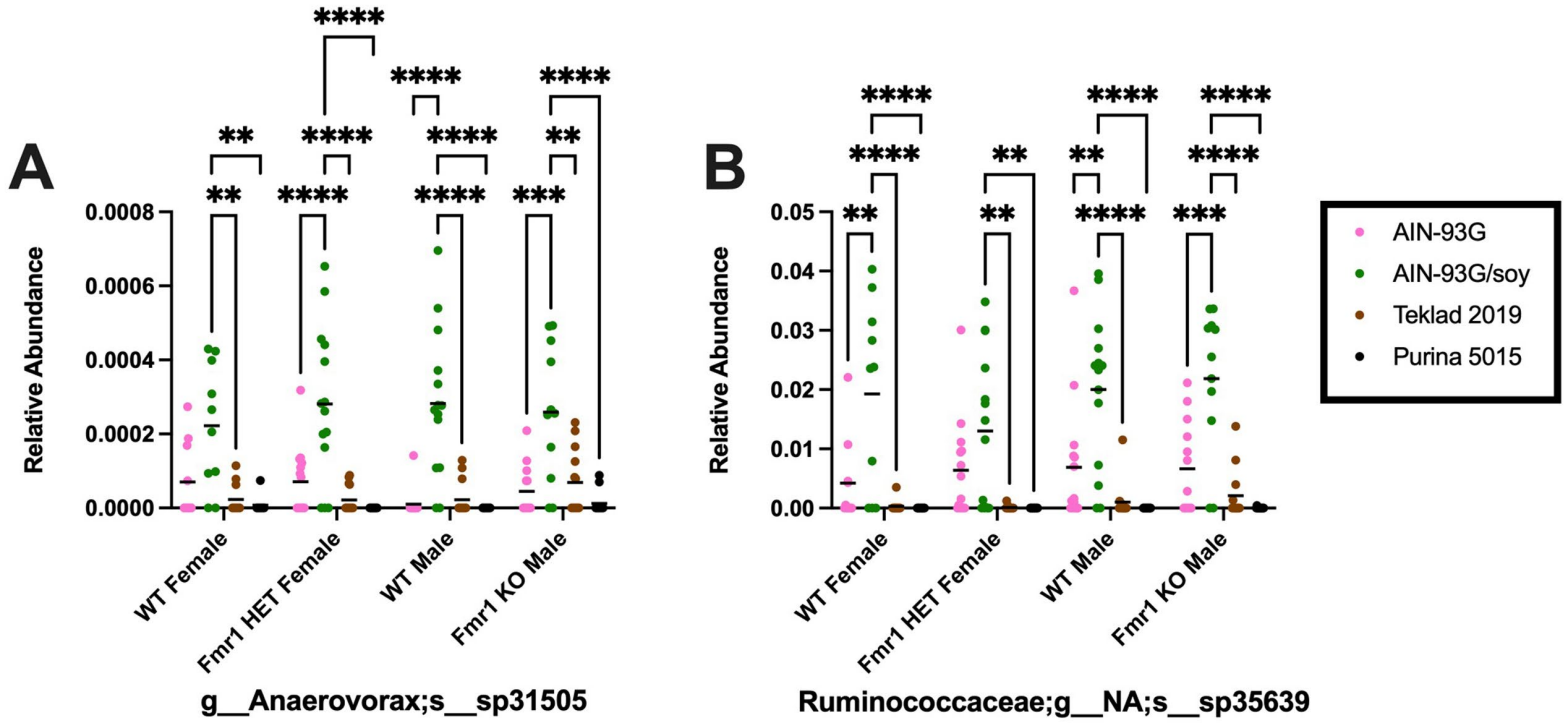
Microbiome relative abundance at the species level as a function of genotype and diet. Relative abundance of positive reads out of the total number of reads after filtering (x-axis) is plotted versus genotype/diet for: **(A)** Anaerovorax;s*__*sp31505, and **(B)** Ruminococcaceae;g*__*NA;s*__*sp35639. Diets are color coded AIN-93G (pink), AIN-93G/soy (green), Teklad 2019 (brown), and Purina 5015 (black). Statistical significance was determined by two-way ANOVA with GraphPad Prism 10, ^**^p < 0.01, ^***^p < 0.001, ^****^p < 0.0001. Error bars comparing diets for each genotype are shown on the graphs. Abbreviations for titles on the x-axis: g, genus; s, species.

At the species level, 681 unique bacteria were identified in male *Fmr1^KO^* cecum. Species members of the p__Firmicutes;c__Clostridia;o__Clostridiales;f*__Family XIII;g__Anaerovorax* (n = 1) and p__Firmicutes;c__Clostridia;o__Clostridiales;*f__Ruminococcaceae;g__NA* (n = 124) genera were analyzed by two-way ANOVA as their genera showed increased bacterial expression as a function of AIN-93G/soy compared to the other three diets, which correlates positively with the FITC-dextran gut permeability data. Two-way ANOVA at the species level indicated genotype-specific differences in *f__Ruminococcaceae;g__NA;s__sp34750* and *sp35799*, but no individual differences were statistically significant. Two-way ANOVA indicated diet-specific differences in numerous species. AIN-93G/soy was associated with elevated *Anaerovorax;s__sp31505* and *Ruminococcaceae;g__NA;s__sp35639* compared to the other three diets in both WT and *Fmr1^KO^* male mice and with two or three of the other diets in WT and *Fmr1^HET^* female mice (Figure 9). Additional species exhibited elevated abundance in response to AIN-93G/soy compared to one more of the other diets in one or more genotypes (Supplementary Figure S10).

AIN-93G was associated with decreased species abundance compared to the other 3 diets in *Ruminococcaceae;g__NA;s__sp34867* (Supplementary Figure S11), which is of interest because audiogenic-induced seizures are significantly attenuated in *Fmr1^KO^* mice in response to casein-based purified ingredient diet (Westmark et al., 2013). Teklad 2019 was associated with elevated species abundance in *Ruminococcaceae;g__NA;s__sp34771* and *sp35181* (Supplementary Figure S12). Purina 5015 was associated with decreased species abundance in *Ruminococcaceae;g__NA;s__sp34750* and increased in *sp35382* and *sp35393-sp35424* (Supplementary Figure S13). The main difference between Purina 5015 and the other diets is the inclusion of porcine animal fat preserved with butylated hydroxyanisole (BHA), butylated hydroxytoluene (BHT) and citric acid, as well as condensed whey and whey solubles. Purified ingredient diets were associated with both elevated and reduced species abundance compared to the chows (Supplementary Figure S14). *Ruminococcaceae;g__NA;s__sp34795-sp34820*, *sp34871*, *sp34878-sp34883*, *sp35736* and *sp35849* were elevated with the purified ingredient diets and *sp35494* was decreased compared to the chows. The main difference between the purified ingredient diets and the chows are the protein sources with casein in AIN-93G; soy in AIN-93G/soy; wheat, corn and yeast in Teklad 2019; and wheat, soy, corn, yeast and whey in Purina 5015.

**Figure 10:**
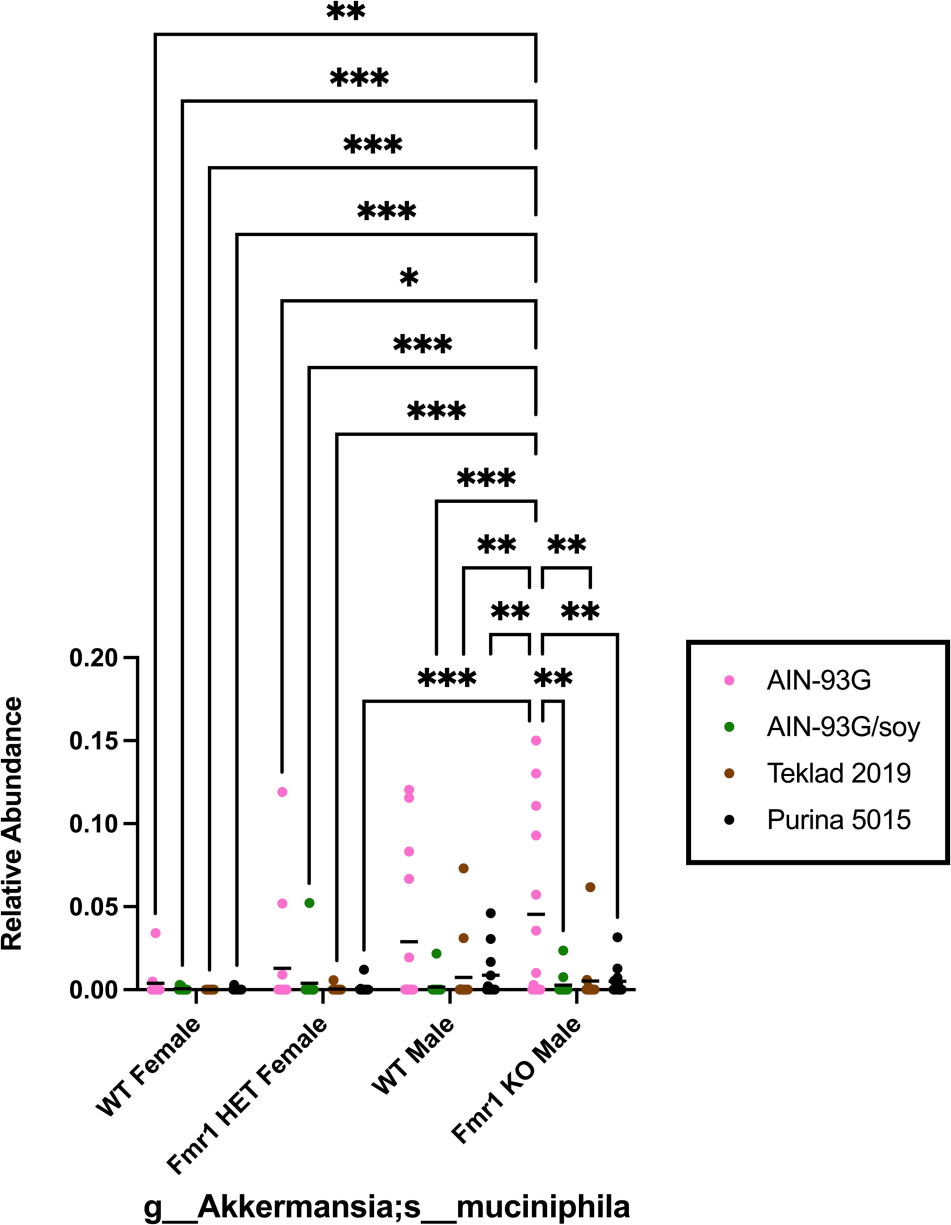
Microbiome relative abundance for *Akkermansia_mucinipila* as a function of genotype and diet. Relative abundance of positive reads out of the total number of reads after filtering (x-axis) is plotted versus genotype/diet for *Akkermansia_mucinipila*. Diets are color coded AIN-93G (pink), AIN-93G/soy (green), Teklad 2019 (brown), and Purina 5015 (black). Statistical significance was determined by two-way ANOVA with GraphPad Prism 10, ^*^p < 0.05, ^**^p < 0.01, ^***^p < 0.001. Abbreviations for titles on the x-axis: g, genus; s, species.

Species within p__Verrucomicrobia;c__Verrucomicrobiae;o__Verrucomicrobiales;*f__ Verrucomicrobiaceae;g__Akkermansia* were analyzed because Verrucomicrobia was the only phylum in this study associated with genotype-specific differences and because prior literature identified this genera and/or species as a biomarker in FXS model mice or in autism (Wang et al., 2011; Goo et al., 2020; Altimiras et al., 2021). *Fmr1^KO^* mice had elevated Verrcuomicrobia compared to female *Fmr1^HET^* and *Fmr1^KO^* mice maintained on AIN-93G. The species *Akkermansia;s__muciniphila* exhibited these genotype significant differences from the phylum down to the species level (Figure 10). In addition, there was elevated abundance with AIN-93G versus the other 3 diets in *Fmr1^KO^* male mice.

## 4 Discussion

### 4.1 Soy protein isolate increases gut permeability

There are few published studies examining gut permeability and/or the microbiome in *Fmr1* models (Supplementary Table S8). We assessed these phenotypes in *Fmr1^KO^* mice in response to four diets (AIN-93G, AIN-93G/soy, Teklad 2019 and Purina 5015). The two standard chows (Teklad 2019 and Purina 5015) were chosen because they are commonly used in our vivarium for routine maintenance of mice. AIN-93G, a purified ingredient diet, was tested because it is routinely employed for diet formulations testing drugs in mice. And AIN-93G/soy, which is matched to AIN-93G with the substitution of SPI for casein protein was included because prior research from our laboratory indicates that soy protein significantly affects *Fmr1^KO^* outcomes.

A significant increase in gut permeability is observed in *Fmr1^HET^* female and WT male mice in response to AIN-93G/soy diet. The soy antigenic proteins *β*-conglycinin and soy glycinin constitute 70–80% of the total protein content of soybeans and induce intestinal barrier damage, impaired goblet cell function, intestinal endoplasmic reticulum stress, autophagic flux blockage and microbiota imbalance in piglets (Wang et al., 2023). To our knowledge Price and colleagues are the only other laboratory that has tested the effect of soy protein isolate on FITC-dextran permeability in mice. Specifically, they tested the effect of soy protein isolate (SPI) on intestinal permeability in C57BL/6 J mice fed a purified ingredient diet containing moderate fat (Price et al., 2021). Permeability was assessed 4 h post dosing and SPI was incorporated into a purified ingredient diet containing 20% of calories from protein, 30% from fat, and 50% from carbohydrate. Intestinal permeability was 0.19 ± 0.02 μg/mL compared to 18.9 ± 5.8 μg/mL in their control mice fed Teklad 8604 chow (contains dehulled soybean meal as major ingredient, 32% calories from protein, 14% calories from fat, 54% from carbohydrate). The findings suggest that the fat content of the diet in conjunction with SPI affects gut permeability where increased fat is protective and increased SPI causes leakage. The methods differ from this study in several respects including mice for the Price study were bred at a different facility (Jackson Laboratories) and transported to the test site, underwent a diet change after weaning but prior to testing, test animals were fed a purified ingredient diet with higher fat than AIN-93G/soy, control animals were fed a chow with more protein than Teklad 2019 or Purina 5015, and blood was collected from mice at four versus 1 h post-FITC treatment. These differences make it difficult to compare absolute permeability rates; however, in both studies, a diet with soy as the only or major protein and 20% or less fat significantly increased intestinal permeability in mice.

Increased gut permeability in response to SPI is also observed in humans. Healthy full-term infants fed soy-based infant formula from birth have high intestinal permeability to lactulose followed by a period of raised permeability to mannitol, although by 6 weeks of age there is no significant difference in intestinal permeability compared to cow milk or human milk (Weaver, 1989).

The effects of SPI on gut permeability may occur through tight junction, immune-mediated, and/or morphine-related mechanisms. It remains to be determined if SPI affects intestinal barrier function through a zonulin-based mechanism as observed in celiac disease. Zonulin levels can increase or decrease in relation to various gut bacteria (Veres-Székely et al., 2023). Soy glycinin and *β*-conglycinin reduce the expression of mucin 2 and tight junction proteins (zonula occludens-1, claudin-1, and occludin) (Wang et al., 2023). Soybean agglutinin increases intestinal permeability and reduces expression of occludin and zonula occludens-1 in piglet mid-jejunum intestine (Zhao et al., 2011). Occludin mRNA is found in FMRP complexes (Lucá et al., 2013). SPI decreases intestinal secretory immunoglobulin A, Th2 cytokine, JAK1/STAT6, mucin expression and the number of goblet cells in C57BL/6 fed AIN-93G formulated with soy versus casein protein (Zeng et al., 2020). Thus, there is attenuation in mouse intestinal immunity in response to SPI. Both casein and soy proteins contain opioid peptides that can mimic the effect of morphine (Tyagi et al., 2020), and inhibit intestinal transit (Daniel et al., 1990; Kaneko et al., 2010).

*Fmr1* genotype-specific differences in FITC-dextran permeability were not observed here or in the literature (Guo et al., 2023). Of interest, metabotropic glutamate receptor 5 (mGluR_5_) acts upstream of FMRP and has been a major drug target for FXS for two decades (Bear et al., 2004). mGluR_5_ is expressed in the gastrointestinal tract and accessory digestive organs (Ferrigno et al., 2017). WT and *mGluR_5_^KO^* mice in a C57BL/6 background exhibit altered microbiome abundance but no difference in gut permeability (Gubert et al., 2020).

### 4.2 The gut microbiome and FXS models

Other studies have examined the gut microbiome in *Fmr1^KO^* mouse models. Altimiras et al. (2021) examined the gut microbiome in C57BL/6 J WT and *Fmr1^KO2^* mice. Differences between their and this study design included mouse strain background (C57BL/6 J versus modified FVB), nature of *Fmr1^KO^* mutation (deletion of the promoter and first exon of the *Fmr1* gene versus insertion of a neomycin resistance cassette into exon 5 of the *Fmr1* locus on the X chromosome), study location (Santiago, Chile versus Wisconsin, United States), caging (ventilated versus static). Similarities included *ad libitum* access to food and water, maintenance on a 12 h light/dark cycle, fasting for 4 h before sample collection from cecum, and identification of the bacterial genome by 16S rRNA sequencing. Their diet was Prolab® RMH 3000, which most closely matches Purina 5015 in this study. In terms of diversity, Altimiras and colleagues found significant differences between WT and *Fmr1^KO2^* littermate mice in 7 phyla (Actinobacteria, Bacteroidetes, Cyanobacteria, Firmicutes, Proteobacteria, Tenericutes and Verrucomicrobia) and 10 genera (*Allobaculum*, *Akkermansia*, *Bacteroides*, *Bifidobacterium*, *Desulfovibrio*, *Flexispira*, *Odoribacter*, *Oscillospira*, *Sutterella* and *Turicibacter*) out of a total of 202 identified bacterial genera. The current study identified bacteria from 6 phyla, which overlapped with the Altimiras study; however, the only statistically significant genotype-specific difference by 2-way ANOVA comparing 4 genotypes (male and female littermate and *Fmr1^KO^* mice) and 4 diets (AIN-93G, AIN-93G/soy, Teklad 2019 and Purina 5015) at the phylum level was Verrucomicrobia, which showed significantly elevated abundance in *Fmr1^KO^* male mice compared to female *Fmr1^HET^* and *Fmr1^KO^* maintained on the AIN-93G diet with no difference compared to WT males. These statistical differences were observed from the phylum down to the species *Akkermansia_muciniphila* level (Figure 10). Comparison of only WT and *Fmr1^KO^* male mice maintained on Purina 5015 indicates that 50% of WT (*n* = 6 of 12) and 46% of *Fmr1^KO^* (*n* = 6 of 13) cecal samples screened positive for *Akkermansia_muciniphila*, and abundance was not statistically different by Student T-Test analysis, *p* = 0.46. In terms of overlap in identified genera between the two studies, this study identified but did not find significant genotype differences in *Bifidobacterium* or *Turicibacter*. These differences highlight the significant effect diet and other extrinsic and intrinsic factors can exert on microbiome populations. Of note, both studies found that Bacteroidetes and Firmicutes were the most prevalent phyla; however, the ratios were reversed with 54% Bacteroidetes and 36% Firmicutes in the Altimiras study and 73% Firmicutes and 23% Bacteriodetes in this study. Neither study found significant differences in alpha diversity.

In contrast to our results with male FVB WT and *Fmr1^KO^* mice, Goo et al. (2020) observed significantly reduced abundance of *Akkermansia_muciniphila* in C57BL/6 J *Fmr1^KO^* mice (~90% reduction), which was rescued by fecal microbiota transplantation from WT mice. Cognitive function and social novelty preference were also rescued by fecal microbiota transplantation.

Guo et al. (2023) analyzed the effects of fish oils on altered gut microbiota and autism behaviors in C57BL/6 J *Fmr1^KO^* mice. The mice were tested at 8 weeks of age and fasted for 12 h prior to sample collection. Principal component analysis of Bray-Curtis distances showed different clusters in beta-diversity between WT and *Fmr1^KO^* mice. The Shannon Index was not statistically different in richness and diversity of microbial species. The most prevalent phyla were Firmicutes (50%) and Bacteroidota (40%) in WT mice and Firmicutes (50%) and Bacteroidota (45%) in *Fmr1^KO^* mice. The mRNA and protein levels for tight junction protein 3 (TJP3) are decreased in the colon of *Fmr1^KO^* mice compared to WT with no differences in other tight junction protein mRNAs or FITC-dextran intestinal permeability after a 4 h fast (~1.7 μg/mL) (Guo et al., 2023). Overall, the percentages of prevalent phyla differed between the Altimiras, Guo and our study while no one found differences in alpha diversity. The Altimiras and Guo studies found altered beta diversity as a function of *Fmr1* genotype, but we did not.

Rude et al. (2019) tested exposure to polychlorinated biphenyls (PCBs) starting 2 weeks before gestation and continuing through postnatal day 21 in double mutant mice expressing a human gain-of-function mutation in RyR1 (T4826I-RYR1) and a human CGG repeat expansion (170–200 CGG repeats) in the *FMR1* gene versus congenic WT (75% C57BL/6, 25% Sv129) mice. The mice were maintained on PicoLab® Mouse Diet 20 (5058), which has ingredients most similar to the Purina 5015 in this study. PCBs cause defects in the mucosal barrier in the ileum and colon with increased tight junction permeability and altered beta diversity. There is no genotype effect on gut permeability until after PCB exposure. Firmicutes (~55%) and Bacteriodetes (~38) were the most abundant phyla, which most closely matches the Guo study,

Varian et al. (2022) found that oral supplementation with the anti-inflammatory probiotic *Lactobacillus reuteri* in outbred conventional CD-1 Swiss stock offspring mice increases FMRP levels and inhibits FXS-like phenotypes. AlOlaby et al. (2022) found differentially methylated genes after *in utero* exposure to *Lactobacillus reuteri*. Nettleton et al. (2021) found that *Lactobacillus reuteri* improves gut barrier integrity, social behavior and repetitive behaviors in BTBR autism mice. Analysis of *Lactobacillus reuteri* as a function of diet was not possible in this study because only 8 mice (*n* = 1 of each genotype on Teklad 2019, *n* = 2 WT males fed AIN-93G/soy, and *n* = 2 WT males fed Purina 5015) screened positive for *Lactobacillus reuteri*.

Salmerón et al. (2024) identified distinct microbiome-derived metabolic profiles in *Fmr1^KO^* Sprague–Dawley rats as function of development. Betaine in the gut is decreased in *Fmr1^KO^* rats at P7. The amino acids tyrosine, isoleucine, alanine, and phenylalanine as well as ethanolamine, lactic acid, and glycerol are elevated at P40. Sterols are reduced, particularly cholesterol. Gut metabolites in response to diet and *Fmr1* genotypes in mice remain to be determined. Plasma-based amino acids are altered in response to diet and genotype (Westmark et al., 2022a, 2024).

Luhur et al. (2017) demonstrated that FMRP is enriched in intestinal progenitor cells and limits the symmetric division and resulting expansion of the stem cell population during adaptive intestinal growth in *Drosophila*. Adult *Fmr1* null mutants have an increased number of progenitor cells and enlarged intestines. Lee et al. (2023) identified *Fmr1* as an essential gene for intestinal stem cell proliferation during gut damage using knockdown RNA interference in *Drosophila*; *Fmr1* transcript expression is upregulated in response to drugs that induce gut damage. It remains to be determined how diet-drug-*Fmr1* gene interactions affect intestinal morphology in mice.

Overall, microbiome findings have important implications for FXS. Ear infections are common in FXS with 63% of boys having six or more otitis infections in the first 5 years of life (Hagerman et al., 1987). Otitis infections are treated with antibiotics, which can affect the diversity of the gut microbiome and lead to digestive issues.

### 4.3 Casein protein increases *Akkermansia muciniphila* in *Fmr1^KO^* mice

The human microbiome is dominated by ~75% Firmicutes and ~ 20% Bacteroidetes, and children with autism have a significant decrease in the ratio of Bacteriodetes/Firmicutes as well as low relative abundance of *Akkermansia_muciniphila* (Wang et al., 2011; Wasilewska and Klukowski, 2015). Decreased abundance of *Akkermansia_muciniphila* is also associated with obesity (Wang et al., 2011; Karlsson et al., 2012). *Akkermansia_muciniphila* is one of two species within the Verrucomibrobia phylum found in the human gastrointestinal tract (Rajilić-Stojanović and de Vos, 2014), and is elevated in *Fmr1^KO^* males compared to WT and *Fmr1^HET^* females fed AIN-93G. *Akkermansia* is a Gram-negative, anaerobic bacteria that can grow on intestinal mucus (Derrien et al., 2004). Casein hydrolysate induces mucin secretion in rat jejunum (Claustre et al., 2002), whereas SPI reduces mucin secretion in mouse intestine (Zeng et al., 2020). Single source casein-based diets are associated with reduced seizures, hyperactivity and body weight in mice (C57BL/6 J background) (Westmark et al., 2013, 2022a,b, 2024), and with reduced seizures, autism, gastrointestinal problems and allergies in humans (Westmark, 2013, 2014a,b, 2016, 2021, 2022; Westmark et al., 2020). Herein, we find a significant decrease in *Akkermansia_muciniphila* abundance in FVB *Fmr1^KO^* mice fed AIN-93G/soy, Teklad 2019 or Purina 5015 compared to AIN-93G. It remains to be determined if diet affects behavioral phenotypes in *Fmr1^KO^* mice in the FVB background. Of note, 69% of *Fmr1^KO^* male mice fed AIN-93G had detectable levels of *Akkermansia_muciniphila*, but only 20% of WT females, 29% of *Fmr1^HET^* females and 50% of WT males.

### 4.4 Sex-specific differences in the microbiome

Of interest, there were numerous sex-specific differences in bacterial abundance. With AIN-93G, *Akkermansia* was increased in *Fmr1^KO^* male mice compared to both WT and *Fmr1^HET^* females. With Teklad 2019, *o__Bacteroidales;f__NA;g__NA* was decreased in WT females versus WT males and *Enterorhabdus* was increased in *Fmr1^HET^* females versus *Fmr1^KO^* males. With Purina 5015, *Enterorhabdus* was increased in WT females versus *Fmr1^KO^* males and *Clostridium* was increased in *Fmr1^KO^* males versus WT and *Fmr1^HET^* females. *Akkermansia*, *Enterorhabus* and *Clastridium* have known roles in intestinal integrity and/or permeability. *Akkermansia* increases intestinal mucus layer thickness and reduces intestinal permeability (Zhang et al., 2024). Species of *Akkermansia*, *Enterorhabdus* and *Clostridium* degrade mucus using mucin as a primary carbon source (Derrien et al., 2004; Derrien et al., 2010). *Clostridium* is found in mucin biofilm and in patients with small intestinal bacterial overgrowth (SIBO) (Macfarlane et al., 2005; Barlow et al., 2021). Thus, altered levels or ratios of these bacteria in *Fmr1^KO^* male mice need further evaluation as potential sex-specific biomarkers and therapeutic targets. We were unable to compare levels in *Fmr1^KO^* female versus *Fmr1^KO^* male mice due to the X-linked nature of the mutation and breeding that predominantly produced litters from WT males. Within sexes on the same diet, there were no statistically significant WT versus *Fmr1*-specific differences down to the genus level.

### 4.5 Confounding issues and future directions

A potentially confounding issue with testing gut permeability with FITC-dextran and dosing based on body weight is that obese animals get dosed with increased levels of FITC-dextran. Voetmann et al. (2023) found that FITC-dextran should be dosed by lean body mass versus total body weight in obese mice to avoid increased permeability due to dosage. Prior studies show that AIN-93G/soy diet significantly increases body weight in *Fmr1^KO^* male mice in the C57BL/6 J background (Westmark et al., 2024). Here, total body weight was not affected by casein versus soy-based AIN-93G in mice in the FVB background. Likewise, the C57BL/6 J mice maintained on their respective diets for multiple generations did not exhibit significant differences in body weight as a function of diet. Increased body weight in males compared to females was not associated with increased gut permeability.

Multiple ingredients vary between the chows and purified ingredient diets. This study focused on the protein source as the major difference between the chows and AIN-93G purified ingredient diet. AIN-93G/soy was synthesized as a matched diet with the sole variable of SPI versus casein. A potential confounding issue in testing the effects of SPI in comparison to casein is that while casein protein-based purified ingredient diets are associated with reduced seizures and body weight in mice, there are also negative health effects including liver steatosis (Dudásová and Grancicová, 1992; Westmark et al., 2024). In this study, several animals with excessively high plasma FITC-dextran levels were outliers and excluded from the analysis. It remains to be determined if casein is causing gastrointestinal perforation. Future work needs to examine the effects of other macronutrients on study outcomes as well as casein and soy in the context of altered fat and carbohydrate content. The bioactive component(s) in or associated with soy that cause increased gut permeability need to be identified. The effect(s) of ingredients such as corn starch, maltodextrin, sucrose and food dyes, which are used in purified ingredient diets need to be examined. Factors affecting variability and outliers need to be identified as well as strain differences in outcome measures. The effects of SPI and standard chows need to be replicated in other animal models.

The microbiota constitutes bacteria, archaea, fungi, microbial eukaryotes and viruses/phages (Allaband et al., 2019). This microbiome analysis only included sequencing of bacterial genes. The cecal content of other microbiota as a function of diet remains to be determined. In addition, different microbiome data analysis methods can give different results even when using the same raw data because algorithms for assigning DNA sequences to particular genomes are approximate and most approaches rely on incomplete reference databases. The choice of PCR probes can also contribute to different results. Despite these potentially confounding issues, multiple studies find significant differences in *Akkermansia_muciniphila* in *Fmr1* mouse models. These findings require follow-up study including assessment of seizure and behavior outcomes in response to treatment with *Akkermansia_muciniphila*.

## 5 Conclusion

In conclusion, diet but not *Fmr1*genotype altered gut permeability and the beta-diversity of the cecal microbiome in mice. Specifically, soy protein in the context of a purified ingredient diet increased FITC-dextran permeability compared to casein-based AIN-93G and Teklad 2019 chow in *Fmr1^HET^* female and WT male FVB mice. Each diet exhibited a distinct cluster on PCoA plots. AIN-93G/soy significantly increased intestinal permeability in C57BL/6 J *Fmr1^KO^* mice compared to AIN-93G and Teklad 2019 in mice maintained on their respective diets for multiple generations. The large effect of diet on the microbiome, and the lack of reporting diet details in publications, could potentially have a large effect on inter-laboratory replicability of studies.

## Data Availability

The original contributions presented in the study are included in the article/Supplementary material, further inquiries can be directed to the corresponding author.

## Footnotes

^1^ https://uwmadison.box.com/s/yajpgaq8r9umqi0j4xy22sjx6boe7wc5
