# Supplementary material for: Soy-based purified ingredient diet affects mouse gut permeability and the microbiome in fragile X mice
Source: Front Mol Neurosci. 2025 Mar 21;18:1520211. doi: 10.3389/fnmol.2025.1520211 (PMC11968763; doi:10.3389/fnmol.2025.1520211)
Supplement: Supplementary file 1 [file Data_Sheet_1.PDF]

Teklad Custom Diet

TD.180374

0.2% Na Control Diet (93G, R)

Formula

Formula	g/Kg
Casein	200.0
L-Cystine	3.0
Corn Starch	394.886
Maltodextrin	132.0
Sucrose	100.0
Soybean Oil	70.0
Cellulose	50.0
Mineral Mix, AIN-93G-MX (94046)	35.0
Sodium Chloride	2.5
Vitamin Mix, AIN-93-VX (94047)	10.0
Choline Bitartrate	2.5
TBHQ, antioxidant	0.014
Red Food Color	0.1

Footnote

Modification of AIN-93G (TD.94045) to increase sodium to 2 g/kg diet (0.2%). Red food dye added for visual differentiation.

Selected Nutrient Information[1]

	% by weight	% kcal from
**Protein**	17.7	18.9
**Carbohydrate**	59.8	63.8
**Fat**	7.2	17.3

Kcal/g **3.7**

[1] Values are calculated from ingredient analysis or manufacturer data

Speak With A Nutritionist

+ **(800) 483·5523**
+ **askanutritionist@envigo.com**

Teklad diets are designed & manufactured for research purposes only.

Key Features

+ **Purified Diet**
+ **AIN-93G Modification**
+ **Sodium Chloride**
+ **Color Coded Red**

Key Planning Information

+ *Products are made fresh to order*
+ Store product at 4°C or lower
+ Use within 6 months (applicable to most diets)
+ Box labeled with product name, manufacturing date, and lot number
+ Replace diet at minimum once per week

  More frequent replacement may be advised
+ Lead time:
  · 2 weeks non-irradiated
  · 4 weeks irradiated

Product Specific Information

+ 1/2" Pellet or Powder (free flowing)
+ Minimum order 3 Kg
+ Irradiation not advised
  · Contact a nutritionist for recommendations

Options (fees will apply)

+ Rush order (pending availability)
+ Irradiation (see Product Specific Information)
+ Vacuum packaging (1 and 2 Kg)

Contact Us

Obtain pricing · Check order status

+ **teklad@envigo.com**
+ **(800) 483·5523**

International Inquiry *(outside USA or Canada)*

+ **askanutritionist@envigo.com**

Place Your Order (USA & Canada)

Please Choose One

+ **www.envigo.com/teklad-orders**
+ **tekladorders@envigo.com**
+ **(800) 483·5523**
+ **(608) 277·2066** *facsimile*

JBF

© 2015 Envigo

05/17/18

Teklad Custom Diet

TD.180375

Soy Protein Diet (93G, G)

Formula

Formula	g/Kg
Isolated Soy Protein	200.0
L-Cystine	1.3
L-Methionine	2.4
Corn Starch	405.726
Maltodextrin	132.0
Sucrose	100.0
Cellulose	50.0
Soybean Oil	70.0
Trace Mineral Mix, AIN-93G (06095)	5.0
Calcium Phosphate, dibasic	9.5
Calcium Carbonate	4.5
Potassium Chloride	6.3
Magnesium Oxide	0.66
Vitamin Mix, AIN-93-VX (94047)	10.0
Choline Bitartrate	2.5
TBHQ, antioxidant	0.014
Green Food Color	0.1

Footnote

Modified from AIN-93G to replace casein with soy protein isolate and to match macrominerals to control diet TD.180374 including: 0.5% Ca, 0.3% Avail. P, 0.2% Na, 0.36% K, 0.3% Cl and 0.05% Mg. Green food dye added for visual differentiation.

Selected Nutrient Information[1]

	% by weight	% kcal from
**Protein**	17.8	18.8
**Carbohydrate**	60.7	64.1
**Fat**	7.2	17.1

Kcal/g 3.8

[1] Values are calculated from ingredient analysis or manufacturer data

Speak With A Nutritionist

+ **(800) 483·5523**
+ **askanutritionist@envigo.com**

Teklad diets are designed & manufactured for research purposes only.

Key Features

+ **Purified Diet**
+ **AIN-93G Modification (Reproduction)**
+ **Soy Protein Isolate**
+ **Matched to Casein Diets**

Key Planning Information

+ *Products are made fresh to order*
+ Store product at 4°C or lower
+ Use within 6 months (applicable to most diets)
+ Box labeled with product name, manufacturing date, and lot number
+ Replace diet at minimum once per week
  More frequent replacement may be advised
+ Lead time:
  · 2 weeks non-irradiated
  · 4 weeks irradiated

Product Specific Information

+ 1/2" Pellet or Powder (free flowing)
+ Minimum order 3 Kg
+ Irradiation not advised
  · Contact a nutritionist for recommendations

Options (fees will apply)

+ Rush order (pending availability)
+ Irradiation (see Product Specific Information)
+ Vacuum packaging (1 and 2 Kg)

Contact Us

Obtain pricing · Check order status

+ **teklad@envigo.com**
+ **(800) 483·5523**

International Inquiry *(outside USA or Canada)*

+ **askanutritionist@envigo.com**

Place Your Order (USA & Canada)

Please Choose One

+ **www.envigo.com/teklad-orders**
+ **tekladorders@envigo.com**
+ **(800) 483·5523**
+ **(608) 277·2066** *facsimile*

JBF

© 2015 Envigo

05/17/18

2019

Teklad Global 19% Protein Extruded Rodent Diet

Product Description- 2019 is a fixed formula, non-autoclavable diet manufactured with high quality ingredients and designed to support gestation, lactation, and growth of rodents. It is particularly useful for poorly breeding strains of rodents and genetically engineered stocks and strains. 2019 does not contain alfalfa or soybean meal, minimizing the occurrence of natural phytoestrogens, which can be beneficial for reproductive toxicology. Typical isoflavone concentrations (daidzein + genistein aglycone equivalents) range from non-detectable to 20 mg/kg. Exclusion of alfalfa reduces chlorophyll, improving optical imaging clarity. Absence of animal protein and fish meal minimizes the presence of nitrosamines. The extruded form ensures that despite its high fat content, it remains firm with minimal wastage. **Also available irradiated (2919). For autoclavable diet, refer to 2019S (Sterilizable).**

**Macronutrients**		
Crude Protein	%	19.0
Fat (acid hydrolysis) [a]	%	9.0
Carbohydrate (available) [b]	%	44.9
Crude Fiber	%	2.6
Neutral Detergent Fiber [c]	%	12.1
Ash	%	5.0
Energy Density [d]	kcal/g (kJ/g)	3.3 (13.8)
Calories from Protein	%	23
Calories from Fat	%	22
Calories from Carbohydrate	%	55
**Minerals**		
Calcium	%	0.9
Phosphorus	%	0.7
Non-Phytate Phosphorus	%	0.4
Sodium	%	0.1
Potassium	%	0.4
Chloride	%	0.4
Magnesium	%	0.2
Zinc	mg/kg	60
Manganese	mg/kg	80
Copper	mg/kg	15
Iodine	mg/kg	6
Iron	mg/kg	200
Selenium	mg/kg	0.23
**Amino Acids**		
Aspartic Acid	%	1.1
Glutamic Acid	%	3.5
Alanine	%	1.2
Glycine	%	0.7
Threonine	%	0.6
Proline	%	1.8
Serine	%	0.9
Leucine	%	2.3
Isoleucine	%	0.8
Valine	%	0.9
Phenylalanine	%	1.1
Tyrosine	%	0.5
Methionine	%	0.5
Cystine	%	0.3
Lysine	%	0.9
Histidine	%	0.4
Arginine	%	0.8
Tryptophan	%	0.2

Teklad Diets are designed and manufactured for research purposes only.

Ingredients (in descending order of inclusion)- Ground wheat, ground corn, corn gluten meal, wheat middlings, soybean oil, calcium carbonate, dicalcium phosphate, brewers dried yeast, L-lysine, iodized salt, magnesium oxide, choline chloride, DL-methionine, calcium propionate, L-tryptophan, vitamin E acetate, menadione sodium bisulfite complex (source of vitamin K activity), manganous oxide, ferrous sulfate, zinc oxide, niacin, calcium pantothenate, copper sulfate, pyridoxine hydrochloride, riboflavin, thiamin mononitrate, vitamin A acetate, calcium iodate, vitamin B_{12} supplement, folic acid, biotin, vitamin D_3 supplement, cobalt carbonate.

Standard Product Form: ***Extruded***

**Vitamins**		
Vitamin A [e, f]	IU/g	15.0
Vitamin D_3 [e, g]	IU/g	1.5
Vitamin E	IU/kg	110
Vitamin K_3 (menadione)	mg/kg	50
Vitamin B_1 (thiamin)	mg/kg	17
Vitamin B_2 (riboflavin)	mg/kg	15
Niacin (nicotinic acid)	mg/kg	75
Vitamin B_6 (pyridoxine)	mg/kg	18
Pantothenic Acid	mg/kg	33
Vitamin B_{12} (cyanocobalamin)	mg/kg	0.08
Biotin	mg/kg	0.40
Folate	mg/kg	4
Choline	mg/kg	1200
**Fatty Acids**		
C16:0 Palmitic	%	0.9
C18:0 Stearic	%	0.2
C18:1ω9 Oleic	%	1.7
C18:2ω6 Linoleic	%	3.9
C18:3ω3 Linolenic	%	0.4
Total Saturated	%	1.2
Total Monounsaturated	%	1.7
Total Polyunsaturated	%	4.4
**Other**		
Cholesterol	mg/kg	--

[a] Ether extract is used to measure fat in pelleted diets, while an acid hydrolysis method is required to recover fat in extruded diets. Compared to ether extract, the fat value for acid hydrolysis will be approximately 1% point higher.

[b] Carbohydrate (available) is calculated by subtracting neutral detergent fiber from total carbohydrates.

[c] Neutral detergent fiber is an estimate of insoluble fiber, including cellulose, hemicellulose, and lignin. Crude fiber methodology underestimates total fiber.

[d] Energy density is a calculated estimate of *metabolizable energy* based on the Atwater factors assigning 4 kcal/g to protein, 9 kcal/g to fat, and 4 kcal/g to available carbohydrate.

[e] Indicates added amount but does not account for contribution from other ingredients.

[f] 1 IU vitamin A = 0.3 μg retinol

[g] 1 IU vitamin D = 25 ng cholecalciferol

For nutrients not listed, insufficient data is available to quantify.

Nutrient data represent the best information available, calculated from published values and direct analytical testing of raw materials and finished product. Nutrient values may vary due to the natural variations in the ingredients, analysis, and effects of processing.

© 2015 Envigo

Mouse Diet 5015*

DESCRIPTION

Mouse Diet is specifically designed to support reproduction, growth and maintenance of mice. This diet is a complete life cycle diet formulated using managed formulation, delivering Constant Nutrition®. This is paired with the selection of highest quality ingredients to assure minimal inherent biological variation in long-term studies. It contains 11% fat to fulfill the metabolic needs of certain mouse strains. Mouse Diet is beneficial in maintaining maximum reproduction for postpartum matings where females are under simultaneous stress of lactation and gestation.

Features and Benefits

- Managed Formulation delivers Constant Nutrition®
- A high-energy diet formulated specifically for all mouse colonies
- Helps maintain maximum reproduction for postpartum matings
- Recommended for mice with low feed intake to improve performance

Product Forms Available	Catalog #
• Oval pellet, 3/8" x 5/8" x 1" length	0001328
• Meal (ground pellets), special order	

Other Versions Available

• 5LJ5 PicoLab® High Energy Mouse Diet	0045341

GUARANTEED ANALYSIS

Crude protein not less than	17.0%
Crude fat not less than	11.0%
Crude fiber not more than	3.0%
Ash not more than	6.5%
Moisture not more than	12.0%

INGREDIENTS

Whole wheat, dehulled soybean meal, ground corn, wheat germ, brewers dried yeast, porcine animal fat preserved with BHA and BHT, condensed whey, porcine animal fat preserved with BHA and citric acid, condensed whey solubles, calcium carbonate, salt, dried whey protein concentrate, soybean oil, mono and diglycerides of edible fats, DL-methionine, dicalcium phosphate, menadione dimethylpyrimidinol bisulfite (source of vitamin K), choline chloride, pyridoxine hydrochloride, cholecalciferol, vitamin A acetate, biotin, dl-alpha tocopheryl acetate (form of vitamin E), folic acid, vitamin B_{12} supplement, thiamine mononitrate, ferrous sulfate, calcium pantothenate, nicotinic acid, riboflavin supplement, manganous oxide, zinc oxide, ferrous carbonate, copper sulfate, zinc sulfate, calcium iodate, cobalt carbonate, sodium selenite.

FEEDING DIRECTIONS

Mouse Diet should be fed to breeders and lactating females on a free-choice basis. Plenty of fresh, clean water should be available to the animals at all times.

Mice-Adult mice will eat up to 5 grams of pelleted ration daily. Some of the larger strains may eat as much as 8 grams per day per animal. Feed should be available on a free choice basis in wire feeders above the floor of the cage.

For information regarding shelf life please visit www.labdiet.com.

CHEMICAL COMPOSITION[1]

Nutrients[2]

**Protein, %**	**18.9**
Arginine, %	1.15
Cystine, %	0.36
Glycine, %	0.79
Histidine, %	0.46
Isoleucine, %	0.83
Leucine, %	1.39
Lysine, %	1.05
Methionine, %	0.62
Phenylalanine, %	0.84
Tyrosine, %	0.53
Threonine, %	0.71
Tryptophan, %	0.24
Valine, %	0.88
Serine, %	1.01
Aspartic Acid, %	2.06
Glutamic Acid, %	4.20
Alanine, %	1.00
Proline, %	1.30
Taurine, %	<0.01
**Fat (ether extract), %**	**11.1**
**Fat (acid hydrolysis), %**	**12.0**
Cholesterol, ppm	32
Linoleic Acid, %	1.96
Linolenic Acid, %	0.15
Arachidonic Acid, %	0.03
Omega-3 Fatty Acids, %	0.21
Total Saturated Fatty Acids, %	3.72
Total Monounsaturated Fatty Acids, %	3.96
**Fiber (Crude), %**	**2.4**
Neutral Detergent Fiber[3], %	10.1
Acid Detergent Fiber[4], %	2.9
**Nitrogen-Free Extract (by difference), %**	**51.8**
Starch, %	32.6
Glucose, %	0.11
Fructose, %	0.11
Sucrose, %	0.90
Lactose, %	2.48
**Total Digestible Nutrients,%**	**85.4**
**Gross Energy, kcal/gm**	**4.74**
**Physiological Fuel Value[5], kcal/gm**	**3.83**
**Metabolizable Energy, kcal/gm**	**3.59**

Minerals

**Ash, %**	**5.7**
Calcium, %	0.80
Phosphorus, %	0.50
Phosphorus (non-phytate), %	0.23
Potassium, %	0.83
Magnesium, %	0.16
Sulfur, %	0.27
Sodium, %	0.43
Chloride, %	0.70
Fluorine, ppm	6.5
Iron, ppm	160
Zinc, ppm	120
Manganese, ppm	120
Copper, ppm	18
Cobalt, ppm	0.63
Iodine, ppm	1.4
Chromium (added), ppm	0.02
Selenium, ppm	0.30

Vitamins

Carotene, ppm	0.20
Vitamin K,ppm	3.0
Thiamin Hydrochloride, ppm	13
Riboflavin, ppm	5.6
Niacin, ppm	74
Pantothenic Acid, ppm	20
Choline Chloride, ppm	2000
Folic Acid, ppm	2.9
Pyridoxine, ppm	9.6
Biotin, ppm	0.30
B_{12}, mcg/kg	51
Vitamin A, IU/gm	18
Vitamin D_3 (added), IU/gm	3.3
Vitamin E, IU/kg	66
Ascorbic Acid, mg/gm	—

Calories provided by:

Protein, %	19.752
Fat (ether extract), %	26.101
Carbohydrates, %	54.148

*Product Code

1. Formulation based on calculated values from the latest ingredient analysis information. Since nutrient composition of natural ingredients varies and some nutrient loss will occur due to manufacturing processes, analysis will differ accordingly.
2. Nutrients expressed as percent of ration except where otherwise indicated. Moisture content is assumed to be 10.0% for the purpose of calculations.
3. NDF = approximately cellulose, hemi-cellulose and lignin.
4. ADF = approximately cellulose and lignin.
5. Physiological Fuel Value (kcal/gm) = Sum of decimal fractions of protein, fat and carbohydrate (use Nitrogen Free Extract) x 4,9,4 kcal/gm respectively.

05/21/15
