# Supplementary material for: Soy-based purified ingredient diet affects mouse gut permeability and the microbiome in fragile X mice
Source: Front Mol Neurosci. 2025 Mar 21;18:1520211. doi: 10.3389/fnmol.2025.1520211 (PMC11968763; doi:10.3389/fnmol.2025.1520211)
Supplement: Supplementary file 2 [file Data_Sheet_2.PDF]

Supplementary Material

1 Supplementary Files

Supplementary File 1: This PDF file contains the formulas for the four diets used in this paper: AIN-93G, AIN-93G/soy, Teklad 2019 and Purina 5015.

Supplementary File 2: This Zip files contains the complete Zymo Research Microbiome Sequencing Service Report containing composition visualization, alpha-diversity, and beta-diversity analyses performed with Qiime v.1.9.1 software.

2 Supplementary Figures and Tables

Supplementary Figure 1. FITC dextran gut permeability in FVB mice. The mice from Figure 1 of the same sex and fed the same diet, regardless of genotype, were combined into a single cohort to increase power to discern differences based on diet. Blood plasma levels of FITC (x-axis) are plotted against sex/diet (y-axis). The AIN-93G cohort (pink) contained n=30 female and n=23 male mice. The AIN-93G/soy cohort (green) contained n=26 female and n=25 male mice. The Teklad 2019 cohort (brown) contained n=35 female and n=27 male mice. The Purina 5015 cohort (black) contained n=25 female and n=24 male mice. Statistical significance was determined by two-way ANOVA with GraphPad Prism 10, *$p<0.05$, **$p<0.01$, ***$p<0.001$, ****$p<0.0001$.

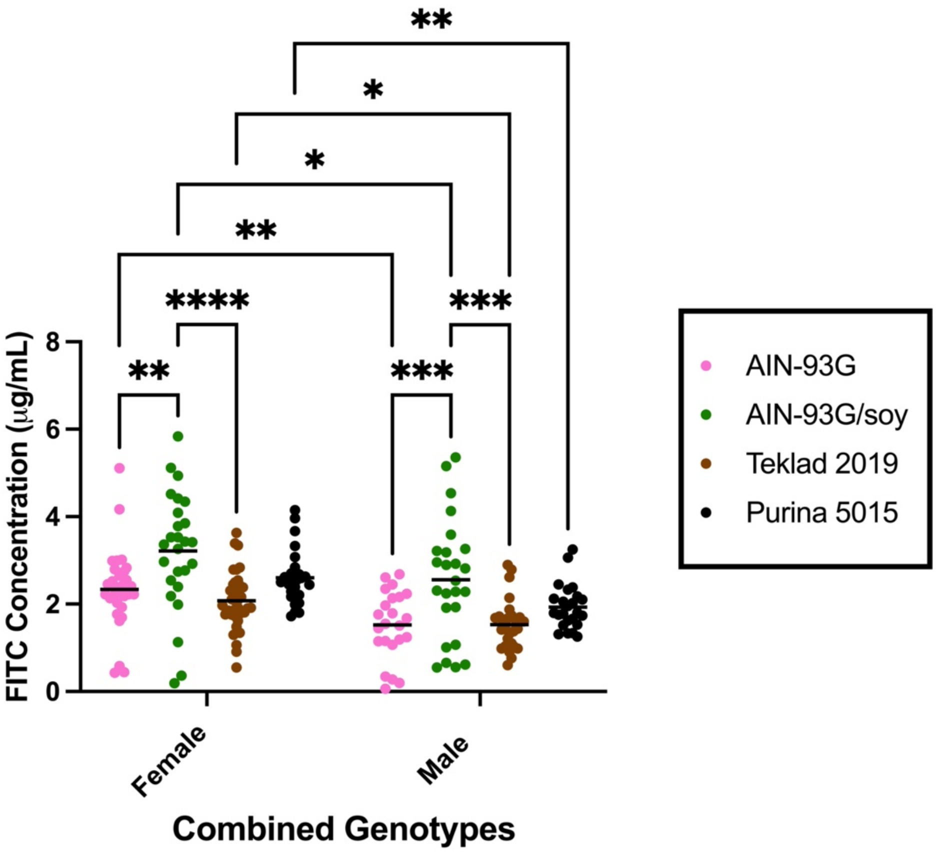

Supplementary Figure 2. Body weight as a function of genotype and diet in mice. **(A)** Female WT, *Fmr1*^{HET}, *Fmr1*^{KO} and male WT and *Fmr1*^{KO} mice in a modified FVB background were maintained on AIN-93G, AIN-93G/soy, Teklad 2019 or Purina 5015 diets and weighed at age P70 prior to fasting. Some mice were oral gavaged with FITC-D4000 (data provided in Figure 1) and others used to collect control plasma for the preparation of standard curves for the FITC assay. Body weight in grams (x-axis) for all mice is plotted here against genotype/diet (y-axis). The AIN-93G cohort (pink) contained WT female (n=18), *Fmr1*^{HET} female (n=36), *Fmr1*^{KO} female (n=9), WT male (n=21) and *Fmr1*^{KO} male (n=23). The AIN-93G/soy cohort (green) contained WT female (n=22), *Fmr1*^{HET} female (n=37), *Fmr1*^{KO} female (n=4), WT male (n=25) and *Fmr1*^{KO} male (n=16). The Teklad 2019 cohort (brown) contained WT female (n=26), *Fmr1*^{HET} female (n=51), *Fmr1*^{KO} female (n=14), WT male (n=36) and *Fmr1*^{KO} male (n=32). The Purina 5015 cohort (black) contained WT female (n=25), *Fmr1*^{HET} female (n=32), *Fmr1*^{KO} female (n=6), WT male (n=17) and *Fmr1*^{KO} male (n=22). **(B)** Female and male *Fmr1*^{KO} mice in the C57BL/6J background were maintained on AIN-93G, AIN-93G/soy or Teklad 2019 for over 10 generations. Mice age P70 were weighed prior to fasting. Some mice were oral gavaged with FITC-D4000 (data provided in Figure 2) and others used to collect control plasma for the preparation of standard curves for the FITC assay. Body weight in grams (x-axis) for all mice is plotted here against sex/diet (y-axis). The AIN-93G cohort (pink) contained *Fmr1*^{KO} female (n=11) and *Fmr1*^{KO} male (n=9). The AIN-93G/soy cohort (green) contained *Fmr1*^{KO} female (n=24) and *Fmr1*^{KO} male (n=17). The Teklad 2019 cohort (brown) contained *Fmr1*^{KO} female (n=20) and *Fmr1*^{KO} male (n=23). Statistical significance was determined by two-way ANOVA with GraphPad Prism 10, *p<0.05, **p<0.01, ***p<0.001, ****p<0.0001.

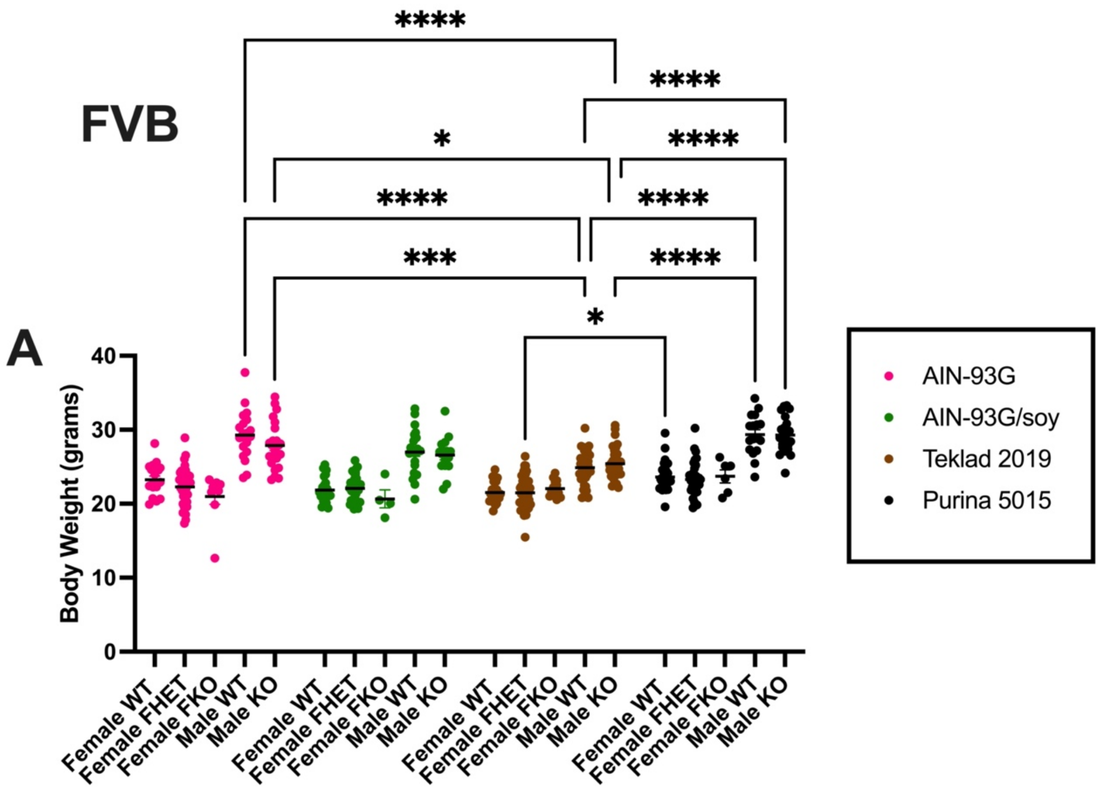
FVB
A
Body Weight (grams)
0
10
20
30
40
Female WT
Female FHET
Female FKO
Male WT
Male KO
AIN-93G
AIN-93G/soy
Teklad 2019
Purina 5015
****
*
***

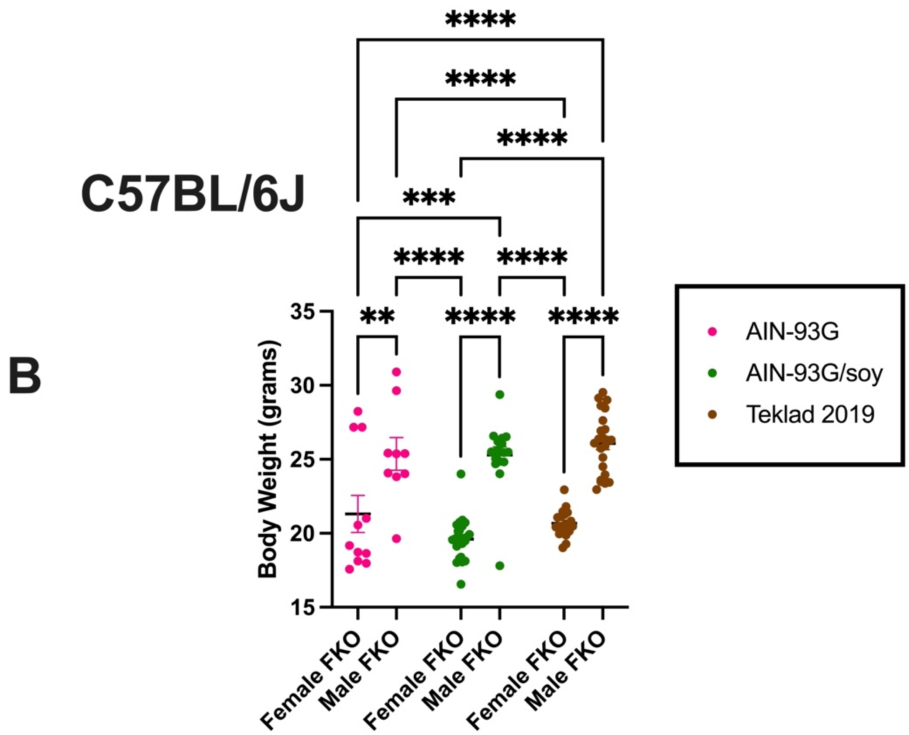
C57BL/6J
B
Body Weight (grams)
15
20
25
30
35
Female FKO
Male FKO
AIN-93G
AIN-93G/soy
Teklad 2019
****
***
**

Supplementary Figure 3. Comparison of body weight in C57BL/6J *Fmr1*^{KO} mice between studies. In Westmark, Lyon et al. 2024, mouse body weight was assessed as a function of age (P3-224), diet and *Fmr1* genotype. Here, the two data points closest to P70 (P67 and P74) are compared with the data from Supplementary Figure 2 for *Fmr1*^{KO} male mice maintained on AIN-93G versus AIN-93G/soy. For the Westmark, Lyon et al. 2024 study, dams were transferred to test diets prior to breeding versus this study where the mice used for breeding were maintained on their respective diets for at least 10 generations. Body weight in grams (x-axis) for all mice is plotted against study/diet (y-axis). The Westmark, Lyon et al 2024 cohort at P67 and P74 contained *Fmr1*^{KO} male on AIN-93G (n=24) and AIN-93G/soy (n=30). The C57BL/6J multigeneration cohort in this study contained *Fmr1*^{KO} male on AIN-93G (n=9) and AIN-93G/soy (n=17). Statistical significance was determined by two-way ANOVA with GraphPad Prism 10, *$p<0.05$, ***$p<0.001$, ****$p<0.0001$.

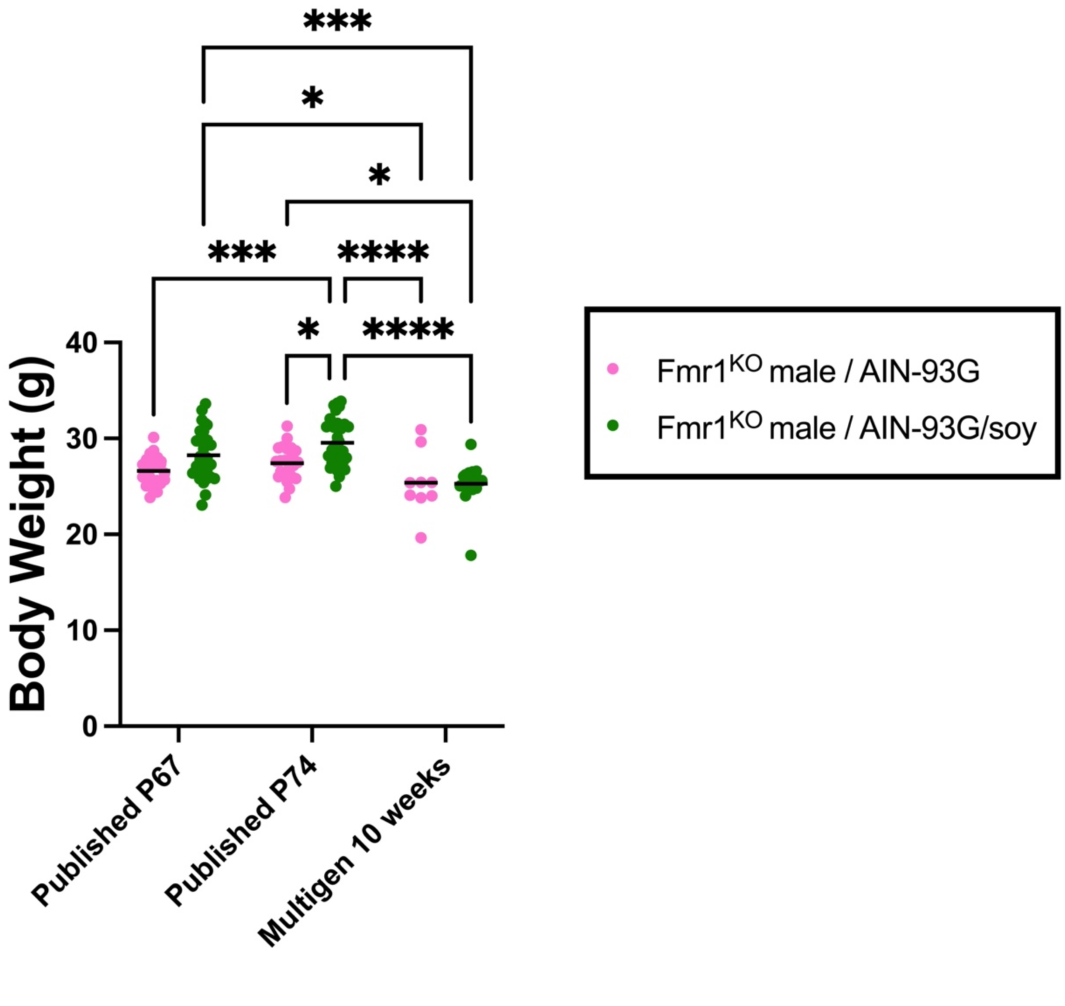

Supplementary Figure 4. LEfSe analysis of WT females as a function of diet. Taxonomies with statistically significant differences ($p < 0.05$) and significant (effect size > 2) differences are plotted as a function of diet. Diets are color coded: Teklad 2019 (brown), AIN-93G/soy (green), Purina 5015 (black), and AIN-93G (pink).

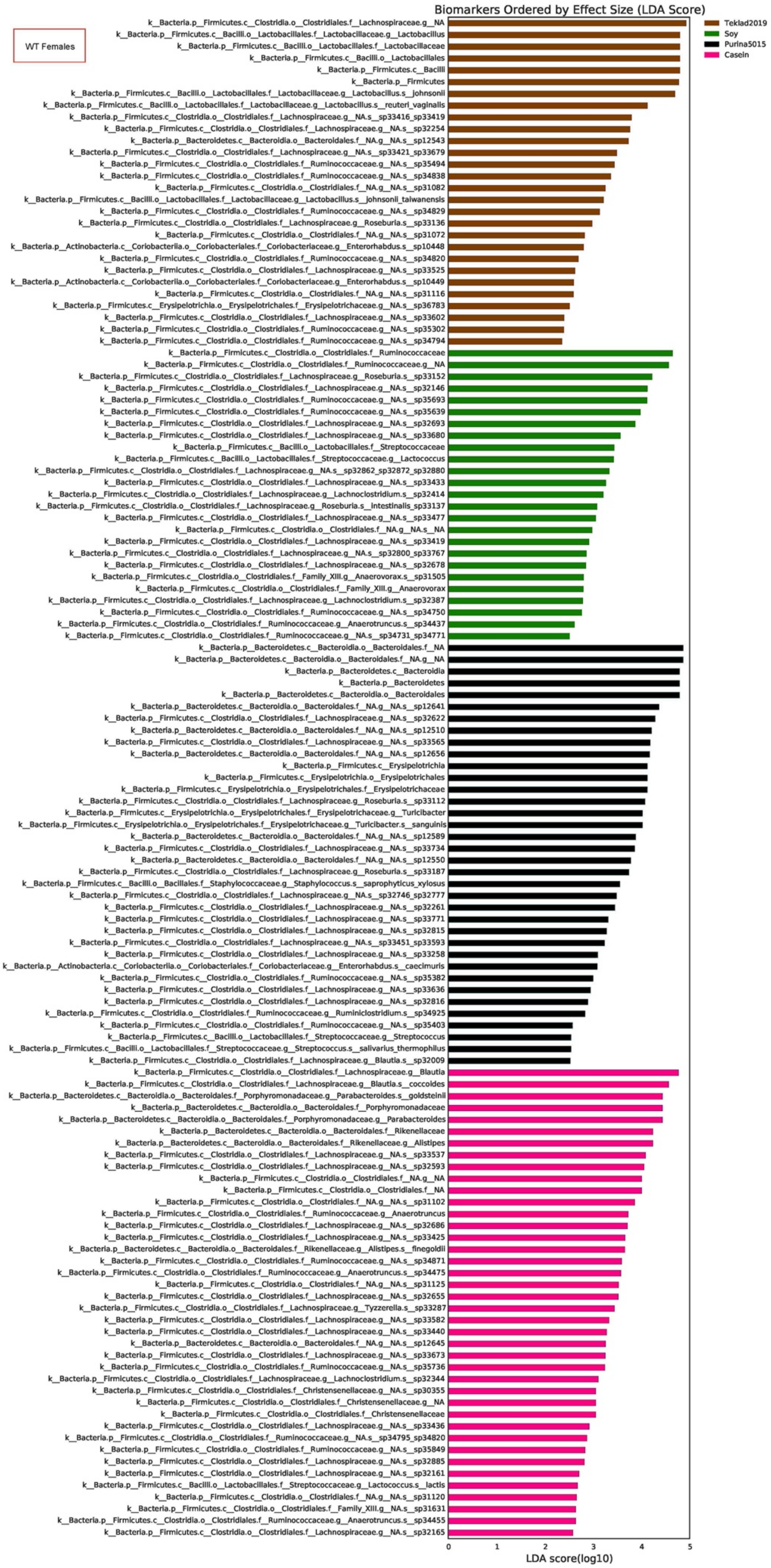
Biomarkers Ordered by Effect Size (LDA Score)
WT Females
Teklad2019
Soy
Purina5015
Casein
k__Bacteria.p__Firmicutes.c__Clostridia.o__Clostridiales.f__Lachnospiraceae.g__NA
k__Bacteria.p__Firmicutes.c__Bacilli.o__Lactobacillales.f__Lactobacillaceae.g__Lactobacillus
k__Bacteria.p__Firmicutes.c__Bacilli.o__Lactobacillales.f__Lactobacillaceae
k__Bacteria.p__Firmicutes.c__Bacilli.o__Lactobacillales
k__Bacteria.p__Firmicutes.c__Bacilli
k__Bacteria.p__Firmicutes
k__Bacteria.p__Firmicutes.c__Bacilli.o__Lactobacillales.f__Lactobacillaceae.g__Lactobacillus.s__johnsonii
k__Bacteria.p__Firmicutes.c__Bacilli.o__Lactobacillales.f__Lactobacillaceae.g__Lactobacillus.s__reuteri_vaginalis
k__Bacteria.p__Firmicutes.c__Clostridia.o__Clostridiales.f__Lachnospiraceae.g__NA.s__sp33416_sp33419
k__Bacteria.p__Firmicutes.c__Clostridia.o__Clostridiales.f__Lachnospiraceae.g__NA.s__sp32254
k__Bacteria.p__Bacteroidetes.c__Bacteroidia.o__Bacteroidales.f__NA.g__NA.s__sp12543
k__Bacteria.p__Firmicutes.c__Clostridia.o__Clostridiales.f__Lachnospiraceae.g__NA.s__sp33421_sp33679
k__Bacteria.p__Firmicutes.c__Clostridia.o__Clostridiales.f__Ruminococcaceae.g__NA.s__sp35494
k__Bacteria.p__Firmicutes.c__Clostridia.o__Clostridiales.f__Ruminococcaceae.g__NA.s__sp34838
k__Bacteria.p__Firmicutes.c__Clostridia.o__Clostridiales.f__NA.g__NA.s__sp31082
k__Bacteria.p__Firmicutes.c__Bacilli.o__Lactobacillales.f__Lactobacillaceae.g__Lactobacillus.s__johnsonii_taiwanensis
k__Bacteria.p__Firmicutes.c__Clostridia.o__Clostridiales.f__Ruminococcaceae.g__NA.s__sp34829
k__Bacteria.p__Firmicutes.c__Clostridia.o__Clostridiales.f__Lachnospiraceae.g__Roseburia.s__sp33136
k__Bacteria.p__Firmicutes.c__Clostridia.o__Clostridiales.f__NA.g__NA.s__sp31072
k__Bacteria.p__Actinobacteria.c__Coriobacteriia.o__Coriobacteriales.f__Coriobacteriaceae.g__Enterorhabdus.s__sp10448
k__Bacteria.p__Firmicutes.c__Clostridia.o__Clostridiales.f__Ruminococcaceae.g__NA.s__sp34820
k__Bacteria.p__Firmicutes.c__Clostridia.o__Clostridiales.f__Lachnospiraceae.g__NA.s__sp33525
k__Bacteria.p__Actinobacteria.c__Coriobacteriia.o__Coriobacteriales.f__Coriobacteriaceae.g__Enterorhabdus.s__sp10449
k__Bacteria.p__Firmicutes.c__Clostridia.o__Clostridiales.f__NA.g__NA.s__sp31116
k__Bacteria.p__Firmicutes.c__Erysipelotrichia.o__Erysipelotrichales.f__Erysipelotrichaceae.g__NA.s__sp36783
k__Bacteria.p__Firmicutes.c__Clostridia.o__Clostridiales.f__Lachnospiraceae.g__NA.s__sp33602
k__Bacteria.p__Firmicutes.c__Clostridia.o__Clostridiales.f__Ruminococcaceae.g__NA.s__sp35302
k__Bacteria.p__Firmicutes.c__Clostridia.o__Clostridiales.f__Ruminococcaceae.g__NA.s__sp34794
k__Bacteria.p__Firmicutes.c__Clostridia.o__Clostridiales.f__Ruminococcaceae
k__Bacteria.p__Firmicutes.c__Clostridia.o__Clostridiales.f__Ruminococcaceae.g__NA
k__Bacteria.p__Firmicutes.c__Clostridia.o__Clostridiales.f__Lachnospiraceae.g__Roseburia.s__sp33152
k__Bacteria.p__Firmicutes.c__Clostridia.o__Clostridiales.f__Lachnospiraceae.g__NA.s__sp32146
k__Bacteria.p__Firmicutes.c__Clostridia.o__Clostridiales.f__Ruminococcaceae.g__NA.s__sp35693
k__Bacteria.p__Firmicutes.c__Clostridia.o__Clostridiales.f__Ruminococcaceae.g__NA.s__sp35639
k__Bacteria.p__Firmicutes.c__Clostridia.o__Clostridiales.f__Lachnospiraceae.g__NA.s__sp32693
k__Bacteria.p__Firmicutes.c__Clostridia.o__Clostridiales.f__Lachnospiraceae.g__NA.s__sp33680
k__Bacteria.p__Firmicutes.c__Bacilli.o__Lactobacillales.f__Streptococcaceae
k__Bacteria.p__Firmicutes.c__Bacilli.o__Lactobacillales.f__Streptococcaceae.g__Lactococcus
k__Bacteria.p__Firmicutes.c__Clostridia.o__Clostridiales.f__Lachnospiraceae.g__NA.s__sp32862_sp32872_sp32880
k__Bacteria.p__Firmicutes.c__Clostridia.o__Clostridiales.f__Lachnospiraceae.g__NA.s__sp33433
k__Bacteria.p__Firmicutes.c__Clostridia.o__Clostridiales.f__Lachnospiraceae.g__Lachnoclostridium.s__sp32414
k__Bacteria.p__Firmicutes.c__Clostridia.o__Clostridiales.f__Lachnospiraceae.g__Roseburia.s__intestinalis_sp33137
k__Bacteria.p__Firmicutes.c__Clostridia.o__Clostridiales.f__Lachnospiraceae.g__NA.s__sp33477
k__Bacteria.p__Firmicutes.c__Clostridia.o__Clostridiales.f__NA.g__NA.s__NA
k__Bacteria.p__Firmicutes.c__Clostridia.o__Clostridiales.f__Lachnospiraceae.g__NA.s__sp33419
k__Bacteria.p__Firmicutes.c__Clostridia.o__Clostridiales.f__Lachnospiraceae.g__NA.s__sp32800_sp33767
k__Bacteria.p__Firmicutes.c__Clostridia.o__Clostridiales.f__Lachnospiraceae.g__NA.s__sp32678
k__Bacteria.p__Firmicutes.c__Clostridia.o__Clostridiales.f__Family_XIII.g__Anaerovorax.s__sp31505
k__Bacteria.p__Firmicutes.c__Clostridia.o__Clostridiales.f__Family_XIII.g__Anaerovorax
k__Bacteria.p__Firmicutes.c__Clostridia.o__Clostridiales.f__Lachnospiraceae.g__Lachnoclostridium.s__sp32387
k__Bacteria.p__Firmicutes.c__Clostridia.o__Clostridiales.f__Ruminococcaceae.g__NA.s__sp34750
k__Bacteria.p__Firmicutes.c__Clostridia.o__Clostridiales.f__Ruminococcaceae.g__Anaerotruncus.s__sp34437
k__Bacteria.p__Firmicutes.c__Clostridia.o__Clostridiales.f__Ruminococcaceae.g__NA.s__sp34731_sp34771
k__Bacteria.p__Bacteroidetes.c__Bacteroidia.o__Bacteroidales.f__NA
k__Bacteria.p__Bacteroidetes.c__Bacteroidia.o__Bacteroidales.f__NA.g__NA
k__Bacteria.p__Bacteroidetes.c__Bacteroidia
k__Bacteria.p__Bacteroidetes
k__Bacteria.p__Bacteroidetes.c__Bacteroidia.o__Bacteroidales
k__Bacteria.p__Bacteroidetes.c__Bacteroidia.o__Bacteroidales.f__NA.g__NA.s__sp12641
k__Bacteria.p__Firmicutes.c__Clostridia.o__Clostridiales.f__Lachnospiraceae.g__NA.s__sp32622
k__Bacteria.p__Bacteroidetes.c__Bacteroidia.o__Bacteroidales.f__NA.g__NA.s__sp12510
k__Bacteria.p__Firmicutes.c__Clostridia.o__Clostridiales.f__Lachnospiraceae.g__NA.s__sp33565
k__Bacteria.p__Bacteroidetes.c__Bacteroidia.o__Bacteroidales.f__NA.g__NA.s__sp12656
k__Bacteria.p__Firmicutes.c__Erysipelotrichia
k__Bacteria.p__Firmicutes.c__Erysipelotrichia.o__Erysipelotrichales
k__Bacteria.p__Firmicutes.c__Erysipelotrichia.o__Erysipelotrichales.f__Erysipelotrichaceae
k__Bacteria.p__Firmicutes.c__Clostridia.o__Clostridiales.f__Lachnospiraceae.g__Roseburia.s__sp33112
k__Bacteria.p__Firmicutes.c__Erysipelotrichia.o__Erysipelotrichales.f__Erysipelotrichaceae.g__Turicibacter
k__Bacteria.p__Firmicutes.c__Erysipelotrichia.o__Erysipelotrichales.f__Erysipelotrichaceae.g__Turicibacter.s__sanguinis
k__Bacteria.p__Bacteroidetes.c__Bacteroidia.o__Bacteroidales.f__NA.g__NA.s__sp12589
k__Bacteria.p__Firmicutes.c__Clostridia.o__Clostridiales.f__Lachnospiraceae.g__NA.s__sp33734
k__Bacteria.p__Bacteroidetes.c__Bacteroidia.o__Bacteroidales.f__NA.g__NA.s__sp12550
k__Bacteria.p__Firmicutes.c__Clostridia.o__Clostridiales.f__Lachnospiraceae.g__Roseburia.s__sp33187
k__Bacteria.p__Firmicutes.c__Bacilli.o__Bacillales.f__Staphylococcaceae.g__Staphylococcus.s__saprophyticus_xylosus
k__Bacteria.p__Firmicutes.c__Clostridia.o__Clostridiales.f__Lachnospiraceae.g__NA.s__sp32746_sp32777
k__Bacteria.p__Firmicutes.c__Clostridia.o__Clostridiales.f__Lachnospiraceae.g__NA.s__sp32261
k__Bacteria.p__Firmicutes.c__Clostridia.o__Clostridiales.f__Lachnospiraceae.g__NA.s__sp33771
k__Bacteria.p__Firmicutes.c__Clostridia.o__Clostridiales.f__Lachnospiraceae.g__NA.s__sp32815
k__Bacteria.p__Firmicutes.c__Clostridia.o__Clostridiales.f__Lachnospiraceae.g__NA.s__sp33451_sp33593
k__Bacteria.p__Firmicutes.c__Clostridia.o__Clostridiales.f__Lachnospiraceae.g__NA.s__sp33258
k__Bacteria.p__Actinobacteria.c__Coriobacteriia.o__Coriobacteriales.f__Coriobacteriaceae.g__Enterorhabdus.s__caecimuris
k__Bacteria.p__Firmicutes.c__Clostridia.o__Clostridiales.f__Ruminococcaceae.g__NA.s__sp35382
k__Bacteria.p__Firmicutes.c__Clostridia.o__Clostridiales.f__Lachnospiraceae.g__NA.s__sp33636
k__Bacteria.p__Firmicutes.c__Clostridia.o__Clostridiales.f__Lachnospiraceae.g__NA.s__sp32816
k__Bacteria.p__Firmicutes.c__Clostridia.o__Clostridiales.f__Ruminococcaceae.g__Ruminiclostridium.s__sp34925
k__Bacteria.p__Firmicutes.c__Clostridia.o__Clostridiales.f__Ruminococcaceae.g__NA.s__sp35403
k__Bacteria.p__Firmicutes.c__Bacilli.o__Lactobacillales.f__Streptococcaceae.g__Streptococcus
k__Bacteria.p__Firmicutes.c__Bacilli.o__Lactobacillales.f__Streptococcaceae.g__Streptococcus.s__salivarius_thermophilus
k__Bacteria.p__Firmicutes.c__Clostridia.o__Clostridiales.f__Lachnospiraceae.g__Blautia.s__sp32009
k__Bacteria.p__Firmicutes.c__Clostridia.o__Clostridiales.f__Lachnospiraceae.g__Blautia
k__Bacteria.p__Firmicutes.c__Clostridia.o__Clostridiales.f__Lachnospiraceae.g__Blautia.s__coccoides
k__Bacteria.p__Bacteroidetes.c__Bacteroidia.o__Bacteroidales.f__Porphyromonadaceae.g__Parabacteroides.s__goldsteinii
k__Bacteria.p__Bacteroidetes.c__Bacteroidia.o__Bacteroidales.f__Porphyromonadaceae
k__Bacteria.p__Bacteroidetes.c__Bacteroidia.o__Bacteroidales.f__Porphyromonadaceae.g__Parabacteroides
k__Bacteria.p__Bacteroidetes.c__Bacteroidia.o__Bacteroidales.f__Rikenellaceae
k__Bacteria.p__Bacteroidetes.c__Bacteroidia.o__Bacteroidales.f__Rikenellaceae.g__Alistipes
k__Bacteria.p__Firmicutes.c__Clostridia.o__Clostridiales.f__Lachnospiraceae.g__NA.s__sp33537
k__Bacteria.p__Firmicutes.c__Clostridia.o__Clostridiales.f__Lachnospiraceae.g__NA.s__sp32593
k__Bacteria.p__Firmicutes.c__Clostridia.o__Clostridiales.f__NA.g__NA
k__Bacteria.p__Firmicutes.c__Clostridia.o__Clostridiales.f__NA
k__Bacteria.p__Firmicutes.c__Clostridia.o__Clostridiales.f__NA.g__NA.s__sp31102
k__Bacteria.p__Firmicutes.c__Clostridia.o__Clostridiales.f__Ruminococcaceae.g__Anaerotruncus
k__Bacteria.p__Firmicutes.c__Clostridia.o__Clostridiales.f__Lachnospiraceae.g__NA.s__sp32686
k__Bacteria.p__Firmicutes.c__Clostridia.o__Clostridiales.f__Lachnospiraceae.g__NA.s__sp33425
k__Bacteria.p__Bacteroidetes.c__Bacteroidia.o__Bacteroidales.f__Rikenellaceae.g__Alistipes.s__finegoldii
k__Bacteria.p__Firmicutes.c__Clostridia.o__Clostridiales.f__Ruminococcaceae.g__NA.s__sp34871
k__Bacteria.p__Firmicutes.c__Clostridia.o__Clostridiales.f__Ruminococcaceae.g__Anaerotruncus.s__sp34475
k__Bacteria.p__Firmicutes.c__Clostridia.o__Clostridiales.f__NA.g__NA.s__sp31125
k__Bacteria.p__Firmicutes.c__Clostridia.o__Clostridiales.f__Lachnospiraceae.g__NA.s__sp32655
k__Bacteria.p__Firmicutes.c__Clostridia.o__Clostridiales.f__Lachnospiraceae.g__Tyzzerella.s__sp33287
k__Bacteria.p__Firmicutes.c__Clostridia.o__Clostridiales.f__Lachnospiraceae.g__NA.s__sp33582
k__Bacteria.p__Firmicutes.c__Clostridia.o__Clostridiales.f__Lachnospiraceae.g__NA.s__sp33440
k__Bacteria.p__Bacteroidetes.c__Bacteroidia.o__Bacteroidales.f__NA.g__NA.s__sp12645
k__Bacteria.p__Firmicutes.c__Clostridia.o__Clostridiales.f__Lachnospiraceae.g__NA.s__sp33673
k__Bacteria.p__Firmicutes.c__Clostridia.o__Clostridiales.f__Ruminococcaceae.g__NA.s__sp35736
k__Bacteria.p__Firmicutes.c__Clostridia.o__Clostridiales.f__Lachnospiraceae.g__Lachnoclostridium.s__sp32344
k__Bacteria.p__Firmicutes.c__Clostridia.o__Clostridiales.f__Christensenellaceae.g__NA.s__sp30355
k__Bacteria.p__Firmicutes.c__Clostridia.o__Clostridiales.f__Christensenellaceae.g__NA
k__Bacteria.p__Firmicutes.c__Clostridia.o__Clostridiales.f__Christensenellaceae
k__Bacteria.p__Firmicutes.c__Clostridia.o__Clostridiales.f__Lachnospiraceae.g__NA.s__sp33436
k__Bacteria.p__Firmicutes.c__Clostridia.o__Clostridiales.f__Ruminococcaceae.g__NA.s__sp34795_sp34820
k__Bacteria.p__Firmicutes.c__Clostridia.o__Clostridiales.f__Ruminococcaceae.g__NA.s__sp35849
k__Bacteria.p__Firmicutes.c__Clostridia.o__Clostridiales.f__Lachnospiraceae.g__NA.s__sp32885
k__Bacteria.p__Firmicutes.c__Clostridia.o__Clostridiales.f__Lachnospiraceae.g__NA.s__sp32161
k__Bacteria.p__Firmicutes.c__Bacilli.o__Lactobacillales.f__Streptococcaceae.g__Lactococcus.s__lactis
k__Bacteria.p__Firmicutes.c__Clostridia.o__Clostridiales.f__NA.g__NA.s__sp31120
k__Bacteria.p__Firmicutes.c__Clostridia.o__Clostridiales.f__Family_XIII.g__NA.s__sp31631
k__Bacteria.p__Firmicutes.c__Clostridia.o__Clostridiales.f__Ruminococcaceae.g__Anaerotruncus.s__sp34455
k__Bacteria.p__Firmicutes.c__Clostridia.o__Clostridiales.f__Lachnospiraceae.g__NA.s__sp32165
0
1
2
3
4
5
LDA score(log10)

Supplementary Figure 5. LEfSe analysis of *Fmr1*^{HET} females as a function of diet. Taxonomies with statistically significant differences ($p < 0.05$) and significant (effect size > 2) differences are plotted as a function of diet. Diets are color coded: Teklad 2019 (brown), AIN-93G/soy (green), Purina 5015 (black), and AIN-93G (pink).

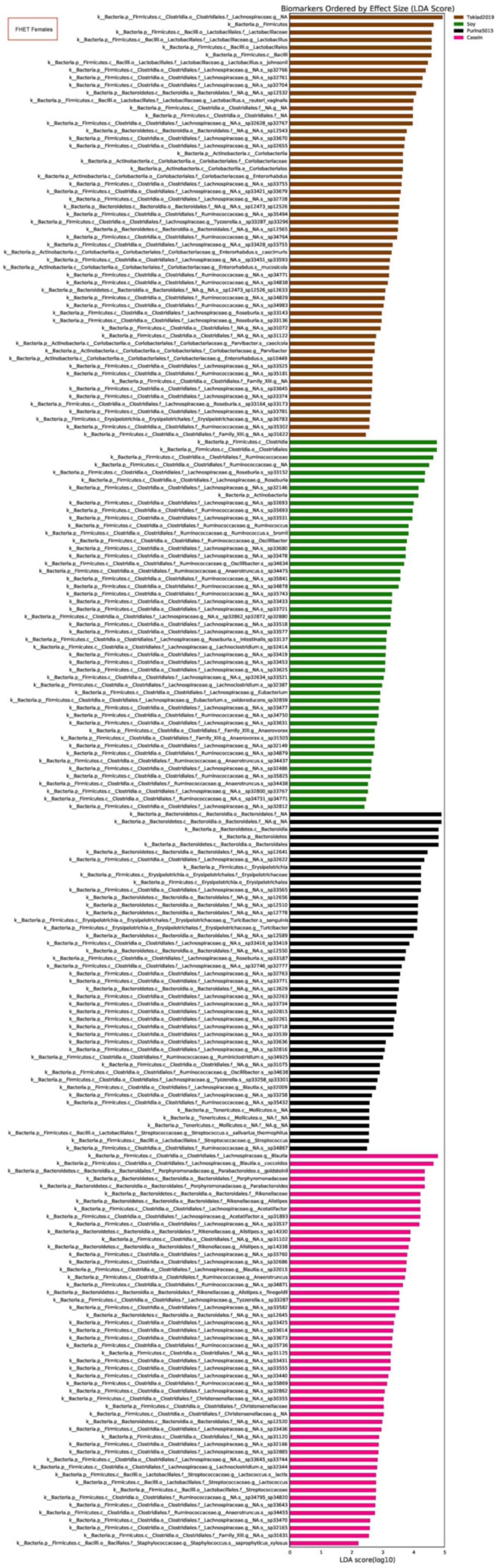
Biomarkers Ordered by Effect Size (LDA Score)
FHET Females
Teklad2019
Soy
Purina5015
Casein
0
1
2
3
4
5
LDA score(log10)

Supplementary Figure 6. LEfSe analysis of WT males as a function of diet. Taxonomies with statistically significant differences ($p < 0.05$) and significant (effect size > 2) differences are plotted as a function of diet. Diets are color coded: Teklad 2019 (brown), AIN-93G/soy (green), Purina 5015 (black), and AIN-93G (pink).

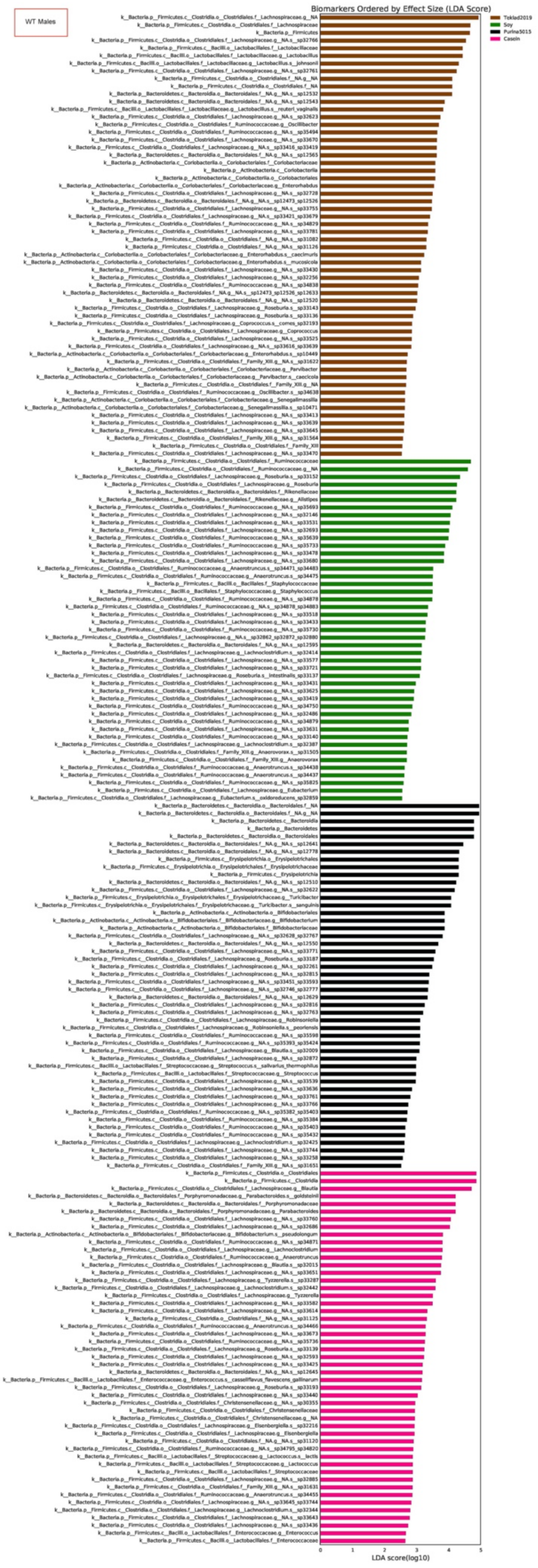
WT Males
Biomarkers Ordered by Effect Size (LDA Score)
Teklad2019
Soy
Purina5015
Casein
LDA score(log10)

Supplementary Figure 7. LEfSe analysis of *Fmr1*^{KO} males as a function of diet. Taxonomies with statistically significant differences ($p < 0.05$) and significant (effect size > 2) differences are plotted as a function of diet. Diets are color coded: Teklad 2019 (brown), AIN-93G/soy (green), Purina 5015 (black), and AIN-93G (pink).

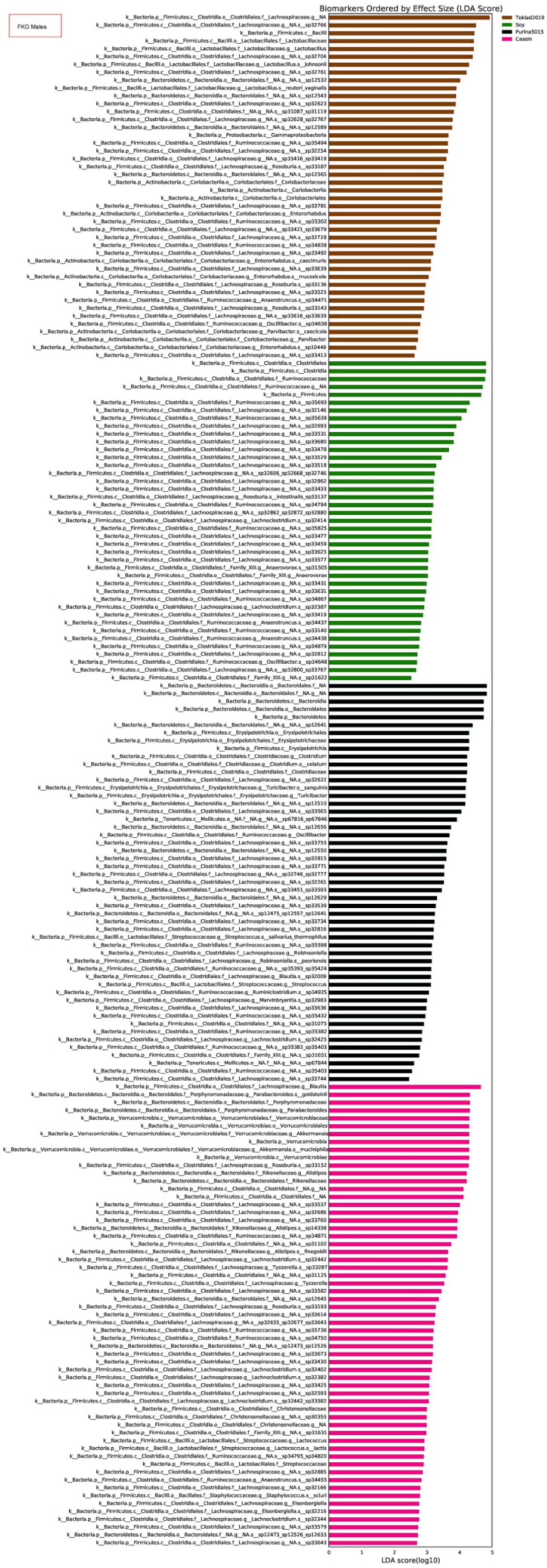
Biomarkers Ordered by Effect Size (LDA Score)
FKO Males
Teklad2019
Soy
Purina5015
Casein
0
1
2
3
4
5
LDA score(log10)

Supplementary Figure 8. Microbiome relative abundance at the family level as a function of genotype and diet. Relative abundance of positive reads out of the total number of reads after filtering (x-axis) is plotted versus genotype/diet for: **(A)** Bifidobacteriaceae, **(B)** Coriobacteriaceae, **(C)** Bacteriodales;f__NA, **(D)** Porphyromonadaceae, **(E)** Rikenellaceae, **(F)** Staphylococcaceae, **(G)** Lactobacillaceae, **(H)** Streptococcaceae, **(I)** Christensenellaceae, **(J)** Clostridiaceae, **(K)** Family XIII, **(L)** Lachnospiraceae, **(M)** Clostridiales;f__NA, **(N)** Peptococcaceae, **(O)** Ruminococcaceae, **(P)** Erysipelotrichaceae, **(Q)** Anaeroplasmataceae, **(R)** c__Mollicutes;o__NA;f__NA, and **(S)** Verrucomicrobiaceae. Diets are color coded AIN-93G (pink), AIN-93G/soy (green), Teklad 2019 (brown), and Purina 5015 (black). Statistical significance was determined by two-way ANOVA with GraphPad Prism 10, *$p<0.05$, **$p<0.01$, ***$p<0.001$, ****$p<0.0001$. Error bars comparing diets for each genotype are shown on the graphs. Abbreviations for titles on the x-axis: o=order, f=family.

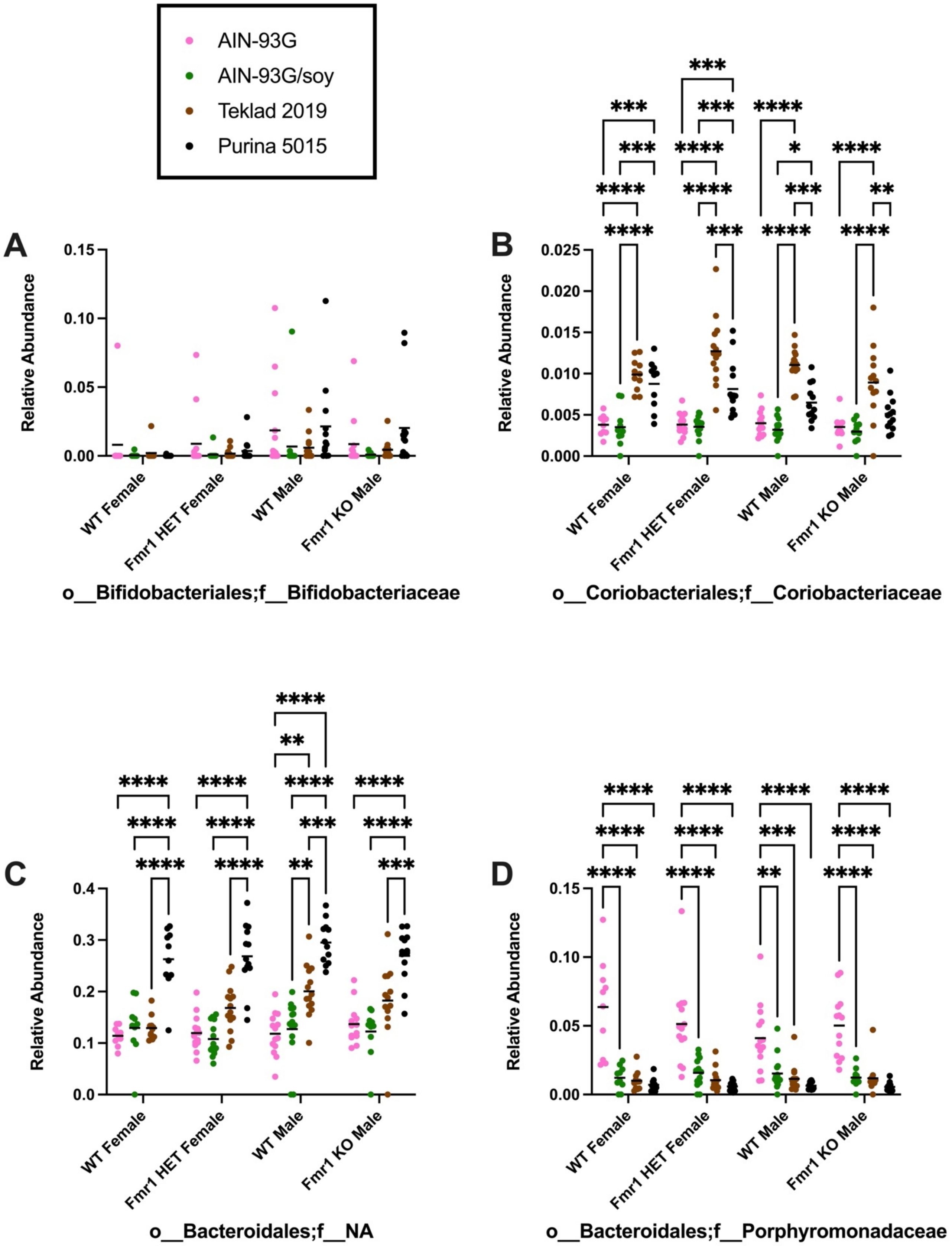
AIN-93G
AIN-93G/soy
Teklad 2019
Purina 5015
A
Relative Abundance
0.15
0.10
0.05
0.00
WT Female
Fmr1 HET Female
WT Male
Fmr1 KO Male
o__Bifidobacteriales;f__Bifidobacteriaceae
B
0.025
0.020
0.015
0.010
0.005
0.000
o__Coriobacteriales;f__Coriobacteriaceae
C
0.4
0.3
0.2
0.1
0.0
o__Bacteroidales;f__NA
D
o__Bacteroidales;f__Porphyromonadaceae

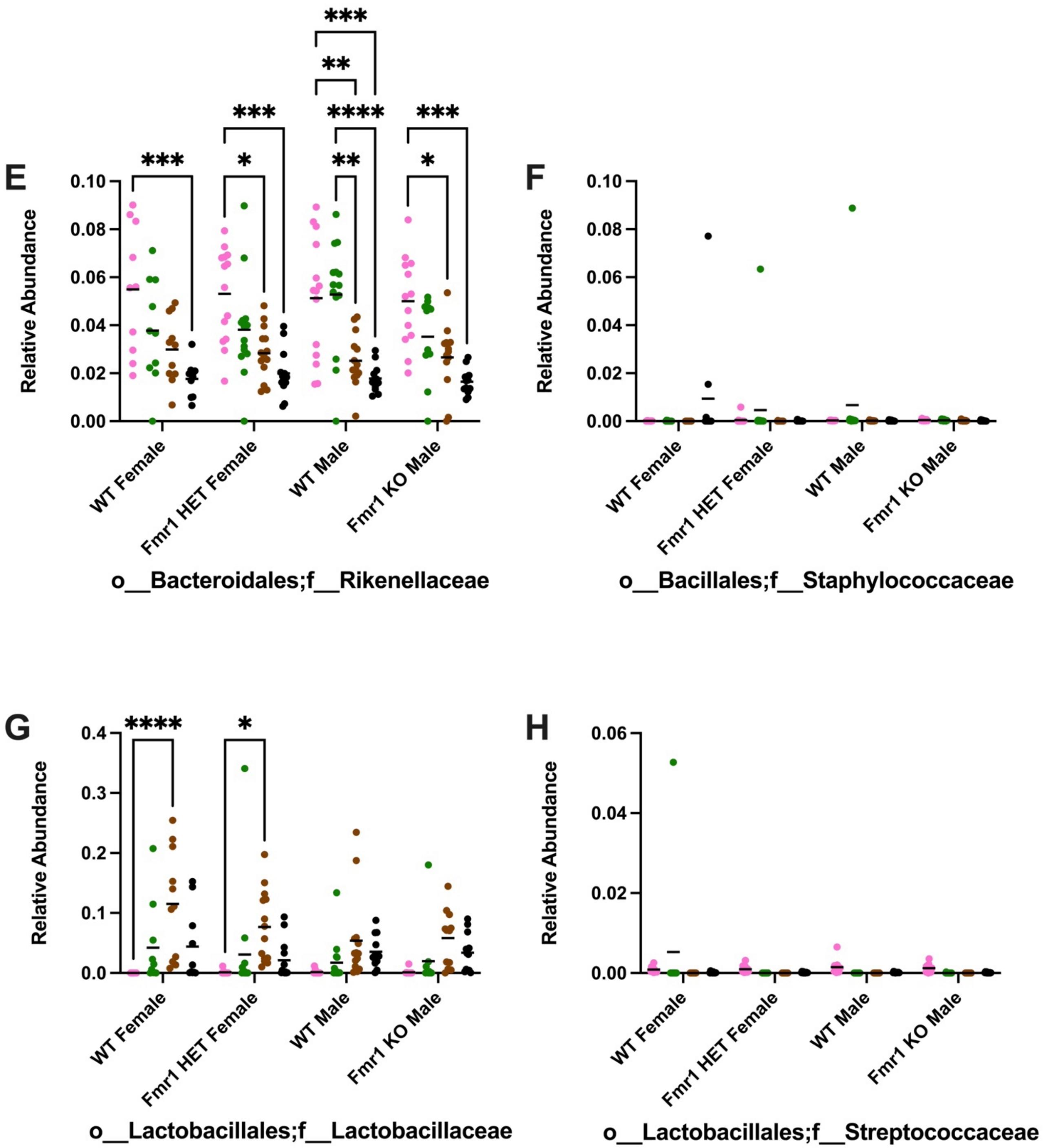
E
F
G
H
Relative Abundance
0.10
0.08
0.06
0.04
0.02
0.00
0.4
0.3
0.2
0.1
0.0
WT Female
Fmr1 HET Female
WT Male
Fmr1 KO Male
***
*
****
**
o__Bacteroidales;f__Rikenellaceae
o__Bacillales;f__Staphylococcaceae
o__Lactobacillales;f__Lactobacillaceae
o__Lactobacillales;f__Streptococcaceae

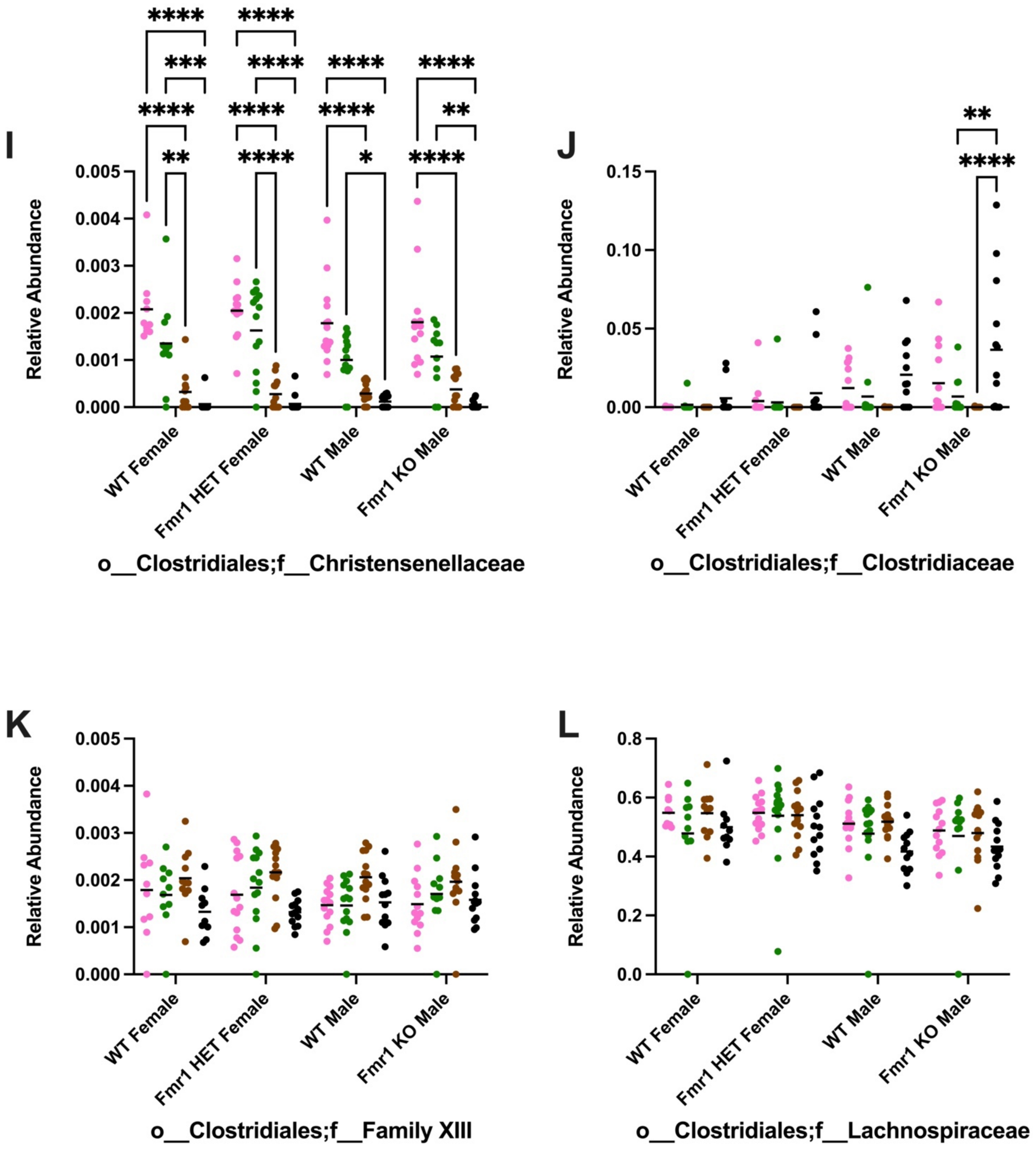
I
Relative Abundance
0.005
0.004
0.003
0.002
0.001
0.000
****
***
****
**
****
****
****
****
****
*
****
****
**
****
WT Female
Fmr1 HET Female
WT Male
Fmr1 KO Male
o__Clostridiales;f__Christensenellaceae
J
Relative Abundance
0.15
0.10
0.05
0.00
**
****
WT Female
Fmr1 HET Female
WT Male
Fmr1 KO Male
o__Clostridiales;f__Clostridiaceae
K
Relative Abundance
0.005
0.004
0.003
0.002
0.001
0.000
WT Female
Fmr1 HET Female
WT Male
Fmr1 KO Male
o__Clostridiales;f__Family XIII
L
Relative Abundance
0.8
0.6
0.4
0.2
0.0
WT Female
Fmr1 HET Female
WT Male
Fmr1 KO Male
o__Clostridiales;f__Lachnospiraceae

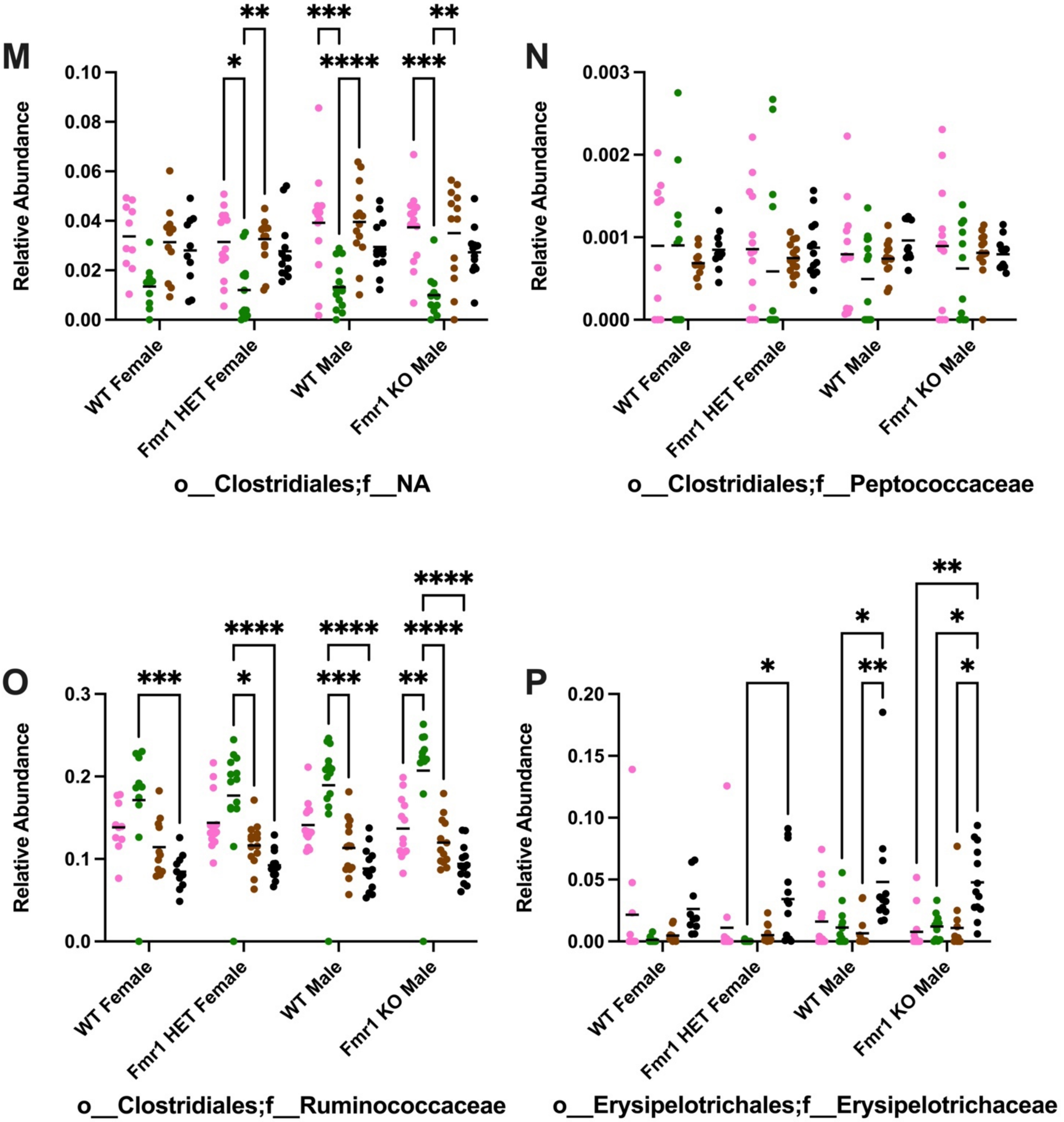
M
Relative Abundance
0.10
0.08
0.06
0.04
0.02
0.00
WT Female
Fmr1 HET Female
WT Male
Fmr1 KO Male
*
**
***
****
***
**
o__Clostridiales;f__NA
N
Relative Abundance
0.003
0.002
0.001
0.000
WT Female
Fmr1 HET Female
WT Male
Fmr1 KO Male
o__Clostridiales;f__Peptococcaceae
O
Relative Abundance
0.3
0.2
0.1
0.0
WT Female
Fmr1 HET Female
WT Male
Fmr1 KO Male
***
*
****
***
****
**
****
****
o__Clostridiales;f__Ruminococcaceae
P
Relative Abundance
0.20
0.15
0.10
0.05
0.00
WT Female
Fmr1 HET Female
WT Male
Fmr1 KO Male
*
*
**
**
*
*
o__Erysipelotrichales;f__Erysipelotrichaceae

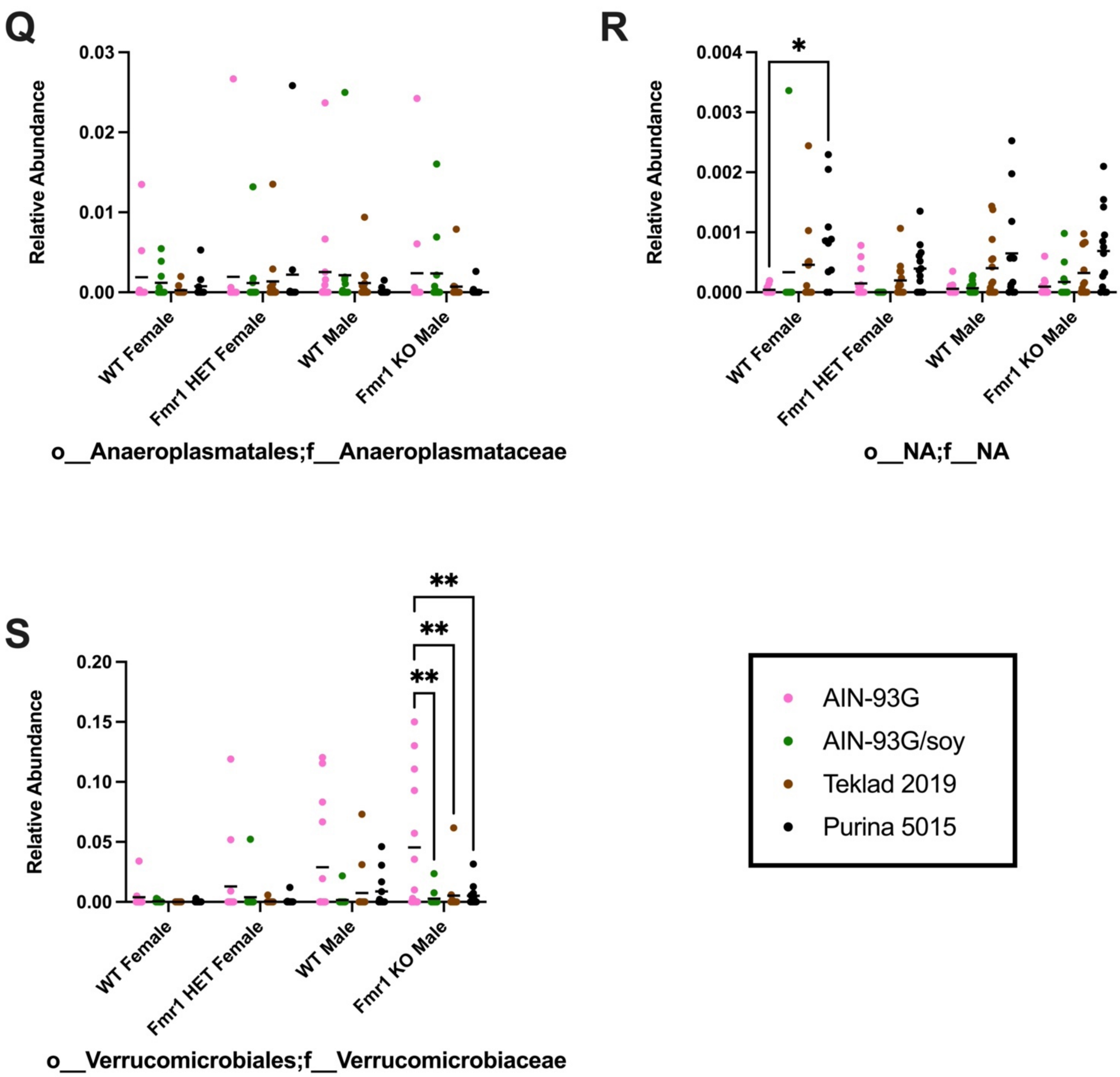
Q
0.03
0.02
0.01
0.00
Relative Abundance
WT Female
Fmr1 HET Female
WT Male
Fmr1 KO Male
o__Anaeroplasmatales;f__Anaeroplasmataceae
R
*
0.004
0.003
0.002
0.001
0.000
Relative Abundance
WT Female
Fmr1 HET Female
WT Male
Fmr1 KO Male
o__NA;f__NA
S
**
**
**
0.20
0.15
0.10
0.05
0.00
Relative Abundance
WT Female
Fmr1 HET Female
WT Male
Fmr1 KO Male
o__Verrucomicrobiales;f__Verrucomicrobiaceae
AIN-93G
AIN-93G/soy
Teklad 2019
Purina 5015

Supplementary Figure 9. Microbiome relative abundance at the genus level as a function of genotype and diet. Relative abundance of positive reads out of the total number of reads after filtering (x-axis) is plotted versus genotype/diet for: **(A)** *Bifidobacterium*, **(B)** *Enterorhabdus*, **(C)** Bacteriodales;f__NA;*g__NA*, **(D)** *Parabacteroides*, **(E)** *Alistipes*, **(F)** *Staphylococcus*, **(G)** *Lactobacillus*, **(H)** *Lactococcus*, **(I)** Christensenellaceae;*g__NA,* **(J)** *Clostridium*, **(K)** *Anaerovorax*, **(L)** Family XIII;*g__NA*, **(M)** *Acetatifactor*, **(N)** *Blautia*, **(O)** *Lachnoclostridium*, **(P)** Lachnospiraceae;*g__NA*, **(Q)** *Robinsoniella*, **(R)** *Roseburia*, **(S)** *Tyzzerella*, **(T)** Clostridiales;f__NA;*g__NA*, **(U)** Peptococcaceae;*g__NA*, **(V)** *Anaerotruncus*, **(W)** Ruminococcaceae;*g__NA*, **(X)** *Oscillibacter*, **(Y)** *Ruminoclostridium*, **(Z)** *Ruminococcus*, **(A2)** Erysipelotrichaceae;*g__NA*, **(B2)** *Turicibacter*, **(C2)** *Anaeroplasma*, **(D2)** Mollicutes;o__NA;f__NA;*g__NA*, and **(E2)** *Akkermansia*. Diets are color coded AIN-93G (pink), AIN-93G/soy (green), Teklad 2019 (brown), and Purina 5015 (black). Statistical significance was determined by two-way ANOVA with GraphPad Prism 10, *p<0.05, **p<0.01, ***p<0.001, ****p<0.0001. Error bars comparing diets for each genotype are shown on the graphs. Abbreviations for titles on the x-axis: c=class, o=order, f=family, g=genus.

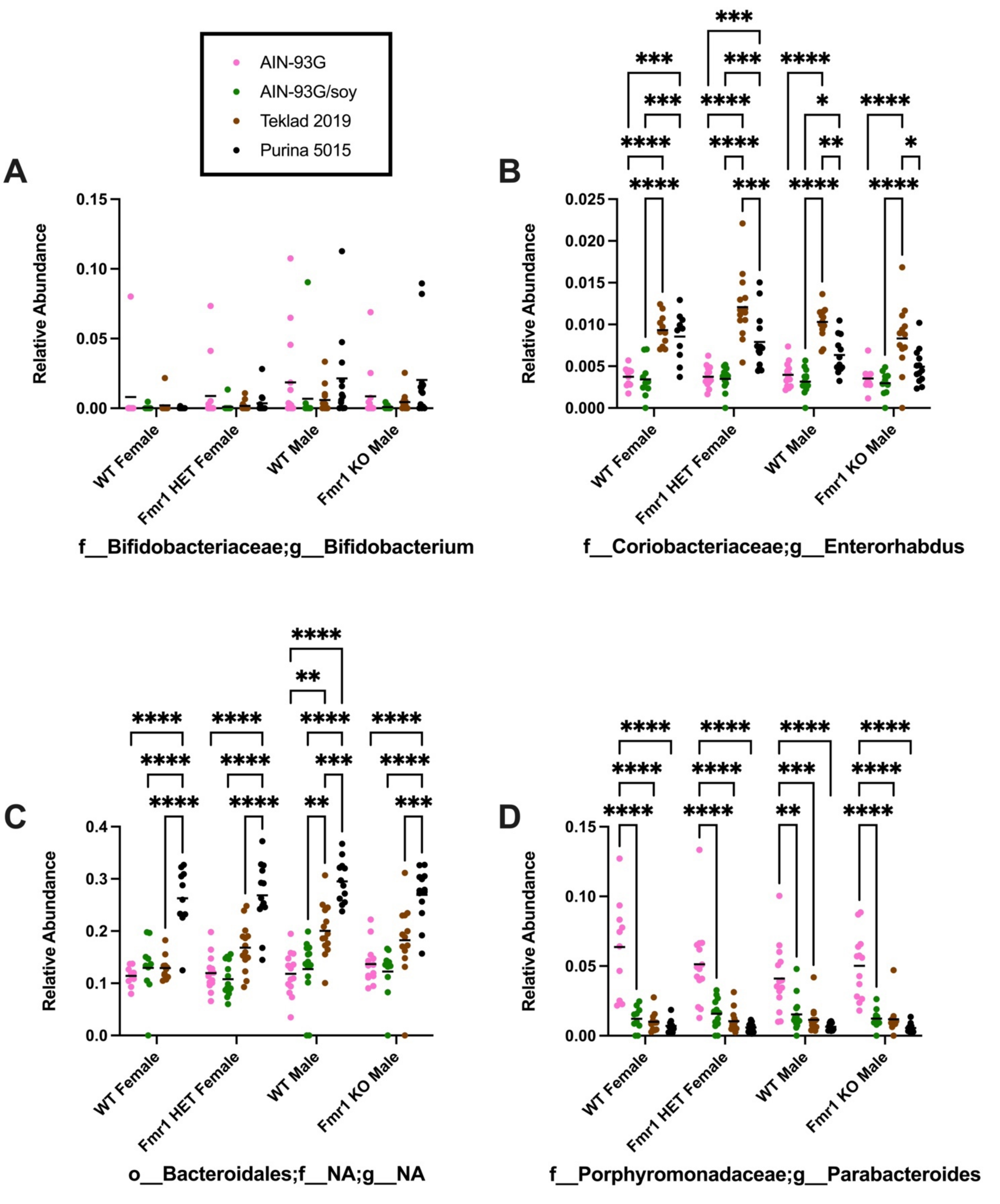

AIN-93G
AIN-93G/soy
Teklad 2019
Purina 5015
A
B
C
D
Relative Abundance
WT Female
Fmr1 HET Female
WT Male
Fmr1 KO Male
f__Bifidobacteriaceae;g__Bifidobacterium
f__Coriobacteriaceae;g__Enterorhabdus
o__Bacteroidales;f__NA;g__NA
f__Porphyromonadaceae;g__Parabacteroides

E

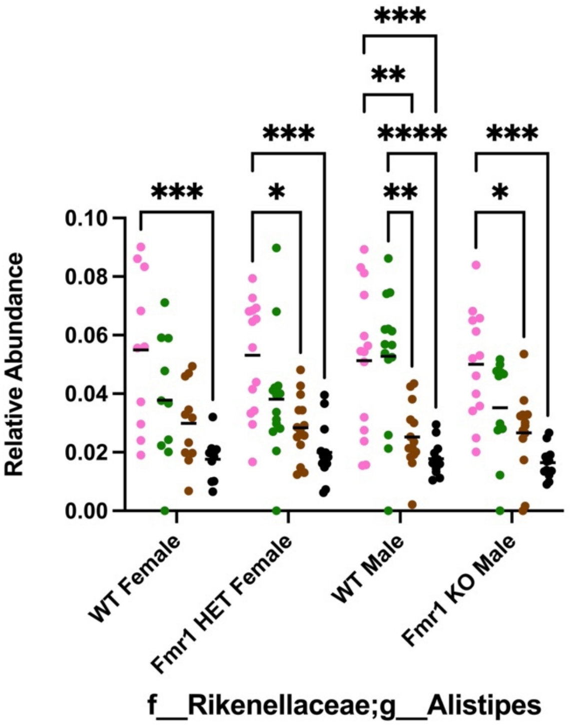
***
**
***
****
***
***
*
**
*
0.10
0.08
0.06
0.04
0.02
0.00
Relative Abundance
WT Female
Fmr1 HET Female
WT Male
Fmr1 KO Male
f__Rikenellaceae;g__Alistipes

F

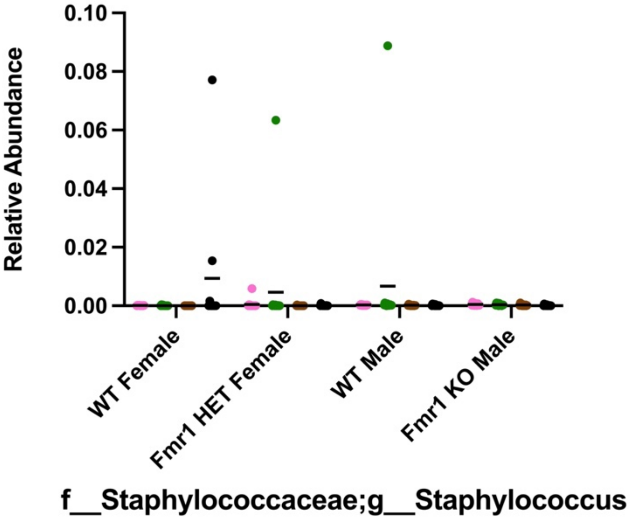
0.10
0.08
0.06
0.04
0.02
0.00
Relative Abundance
WT Female
Fmr1 HET Female
WT Male
Fmr1 KO Male
f__Staphylococcaceae;g__Staphylococcus

G

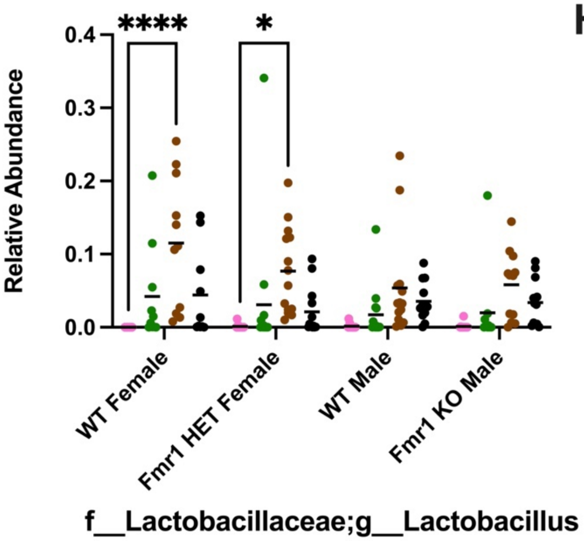
****
*
0.4
0.3
0.2
0.1
0.0
Relative Abundance
WT Female
Fmr1 HET Female
WT Male
Fmr1 KO Male
f__Lactobacillaceae;g__Lactobacillus

H

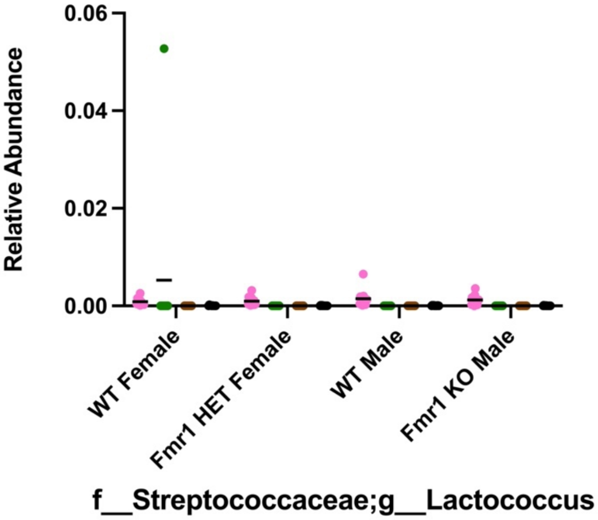
0.06
0.04
0.02
0.00
Relative Abundance
WT Female
Fmr1 HET Female
WT Male
Fmr1 KO Male
f__Streptococcaceae;g__Lactococcus

I

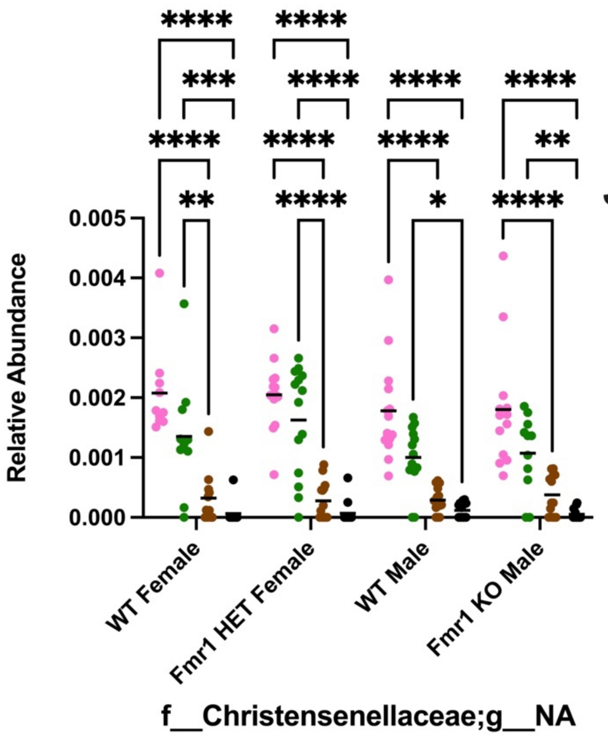
****
****
***
****
****
****
****
****
****
**
**
****
*
****
0.005
0.004
0.003
0.002
0.001
0.000
Relative Abundance
WT Female
Fmr1 HET Female
WT Male
Fmr1 KO Male
f__Christensenellaceae;g__NA

J

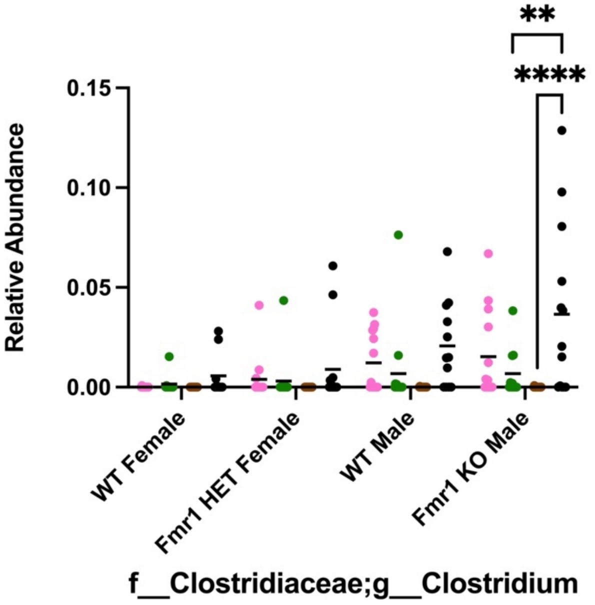
**
****
0.15
0.10
0.05
0.00
Relative Abundance
WT Female
Fmr1 HET Female
WT Male
Fmr1 KO Male
f__Clostridiaceae;g__Clostridium

K

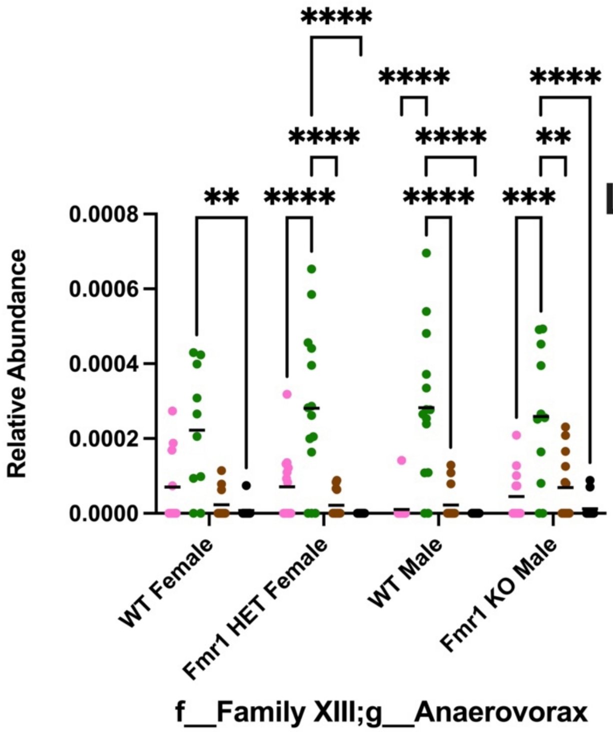
****
****
****
****
****
**
**
****
****
***
0.0008
0.0006
0.0004
0.0002
0.0000
Relative Abundance
WT Female
Fmr1 HET Female
WT Male
Fmr1 KO Male
f__Family XIII;g__Anaerovorax

L

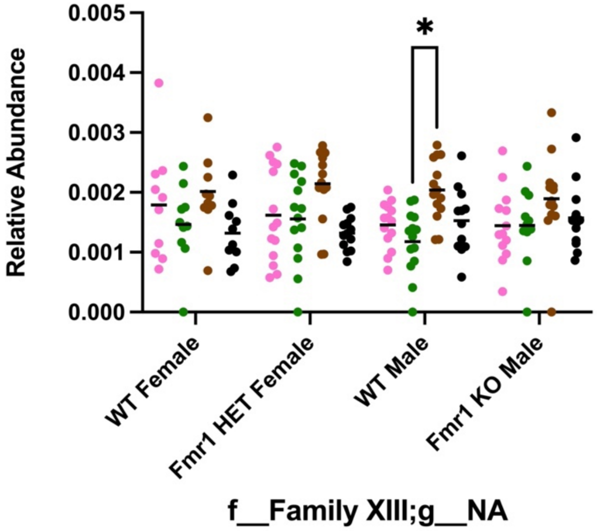
*
0.005
0.004
0.003
0.002
0.001
0.000
Relative Abundance
WT Female
Fmr1 HET Female
WT Male
Fmr1 KO Male
f__Family XIII;g__NA

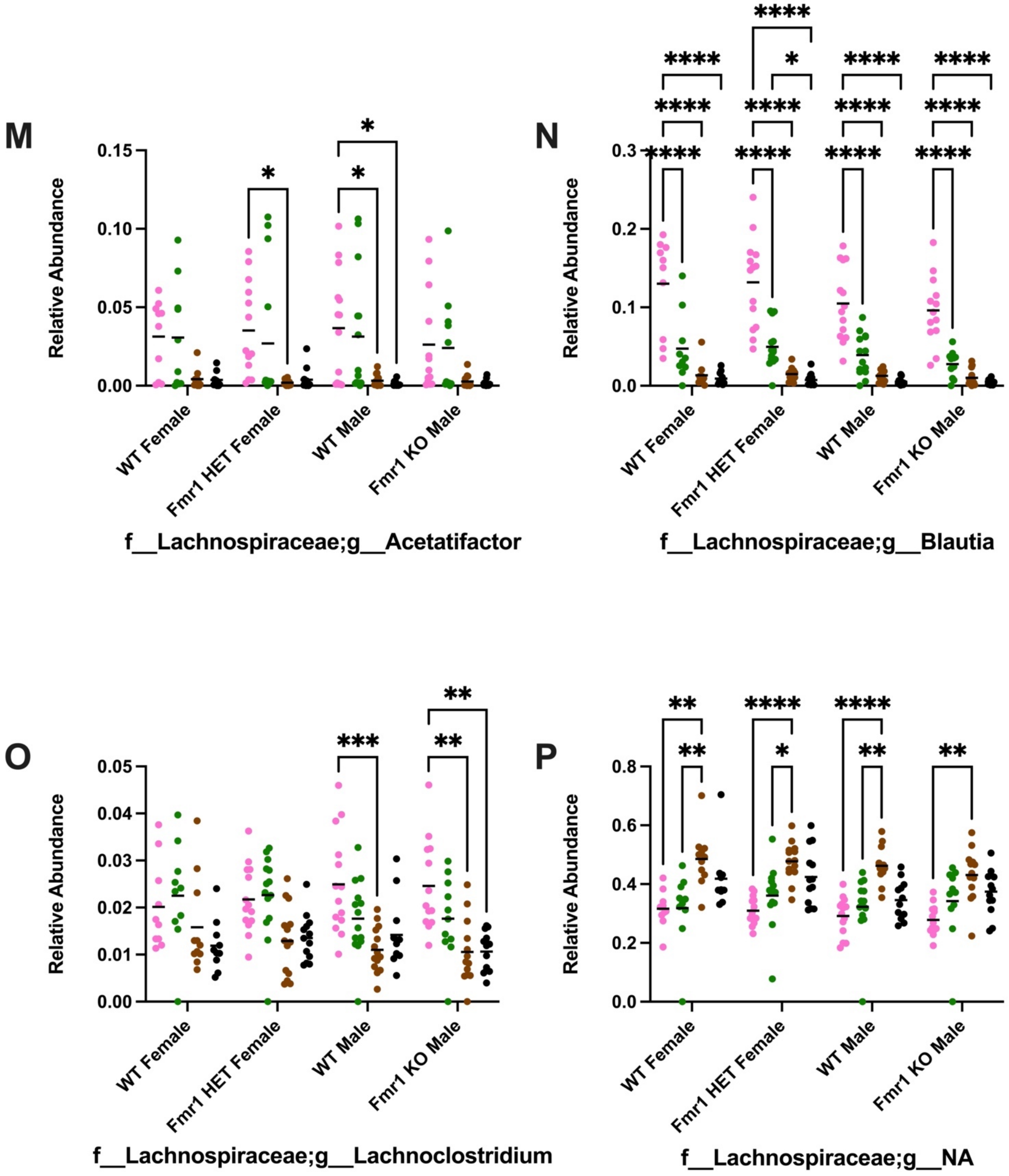
M
Relative Abundance
0.15
0.10
0.05
0.00
WT Female
Fmr1 HET Female
WT Male
Fmr1 KO Male
f__Lachnospiraceae;g__Acetatifactor
N
Relative Abundance
0.3
0.2
0.1
0.0
WT Female
Fmr1 HET Female
WT Male
Fmr1 KO Male
f__Lachnospiraceae;g__Blautia
O
Relative Abundance
0.05
0.04
0.03
0.02
0.01
0.00
WT Female
Fmr1 HET Female
WT Male
Fmr1 KO Male
f__Lachnospiraceae;g__Lachnoclostridium
P
Relative Abundance
0.8
0.6
0.4
0.2
0.0
WT Female
Fmr1 HET Female
WT Male
Fmr1 KO Male
f__Lachnospiraceae;g__NA

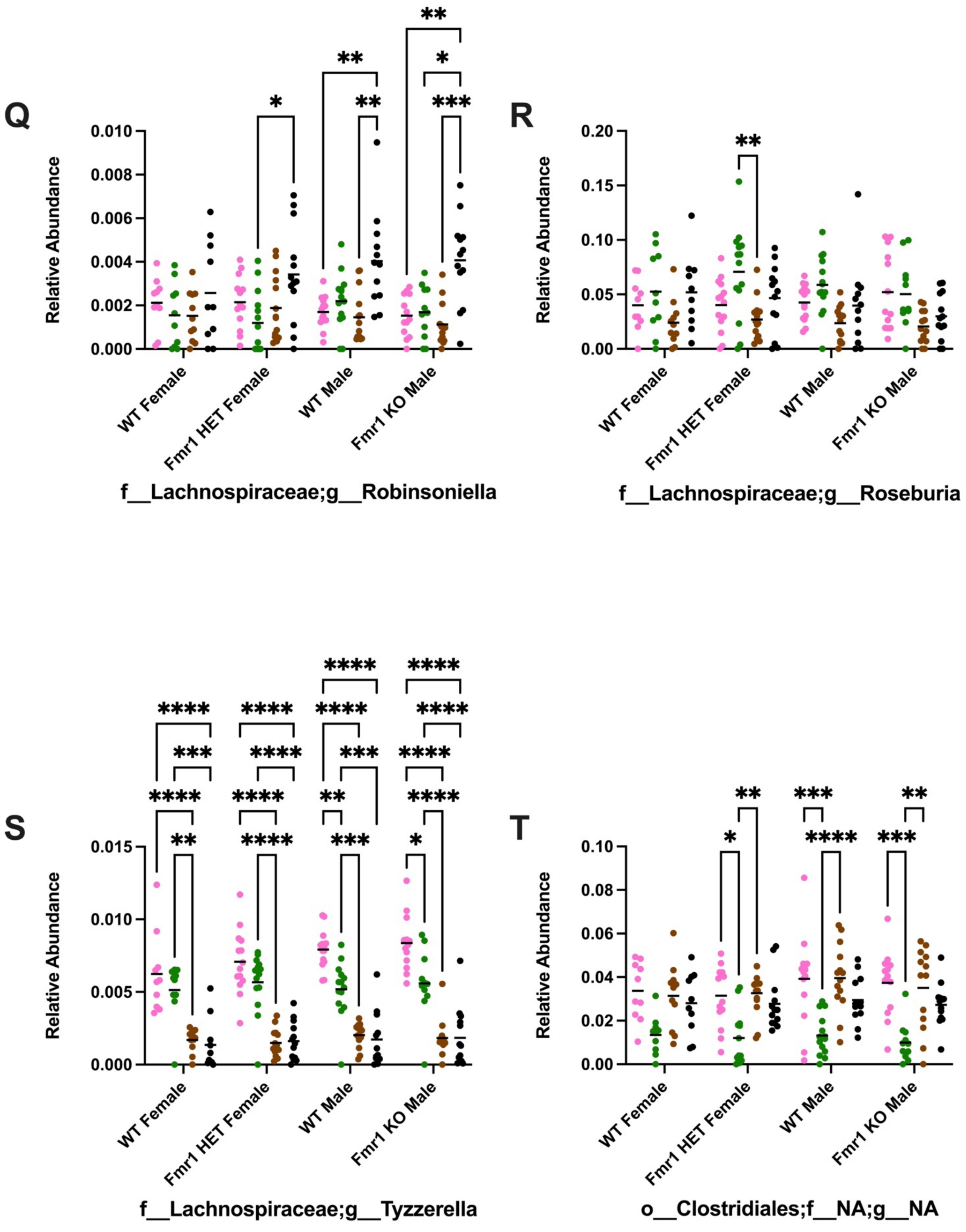
Q
Relative Abundance
0.010
0.008
0.006
0.004
0.002
0.000
WT Female
Fmr1 HET Female
WT Male
Fmr1 KO Male
f__Lachnospiraceae;g__Robinsoniella
R
Relative Abundance
0.20
0.15
0.10
0.05
0.00
WT Female
Fmr1 HET Female
WT Male
Fmr1 KO Male
f__Lachnospiraceae;g__Roseburia
S
Relative Abundance
0.015
0.010
0.005
0.000
WT Female
Fmr1 HET Female
WT Male
Fmr1 KO Male
f__Lachnospiraceae;g__Tyzzerella
T
Relative Abundance
0.10
0.08
0.06
0.04
0.02
0.00
WT Female
Fmr1 HET Female
WT Male
Fmr1 KO Male
o__Clostridiales;f__NA;g__NA

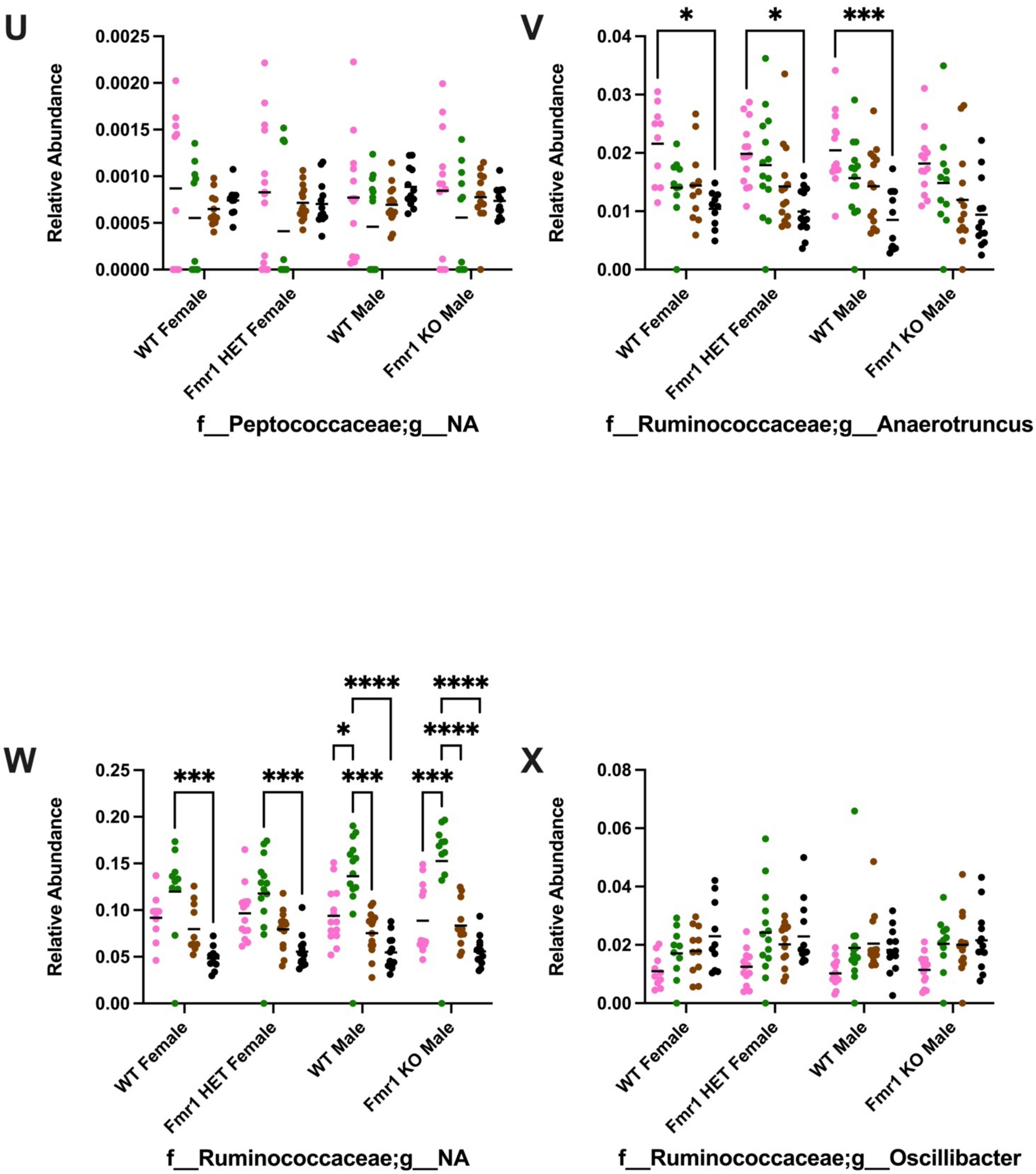
U
Relative Abundance
0.0025
0.0020
0.0015
0.0010
0.0005
0.0000
WT Female
Fmr1 HET Female
WT Male
Fmr1 KO Male
f__Peptococcaceae;g__NA
V
*
*
***
Relative Abundance
0.04
0.03
0.02
0.01
0.00
WT Female
Fmr1 HET Female
WT Male
Fmr1 KO Male
f__Ruminococcaceae;g__Anaerotruncus
W
****
****
*
****
***
***
***
***
Relative Abundance
0.25
0.20
0.15
0.10
0.05
0.00
WT Female
Fmr1 HET Female
WT Male
Fmr1 KO Male
f__Ruminococcaceae;g__NA
X
Relative Abundance
0.08
0.06
0.04
0.02
0.00
WT Female
Fmr1 HET Female
WT Male
Fmr1 KO Male
f__Ruminococcaceae;g__Oscillibacter

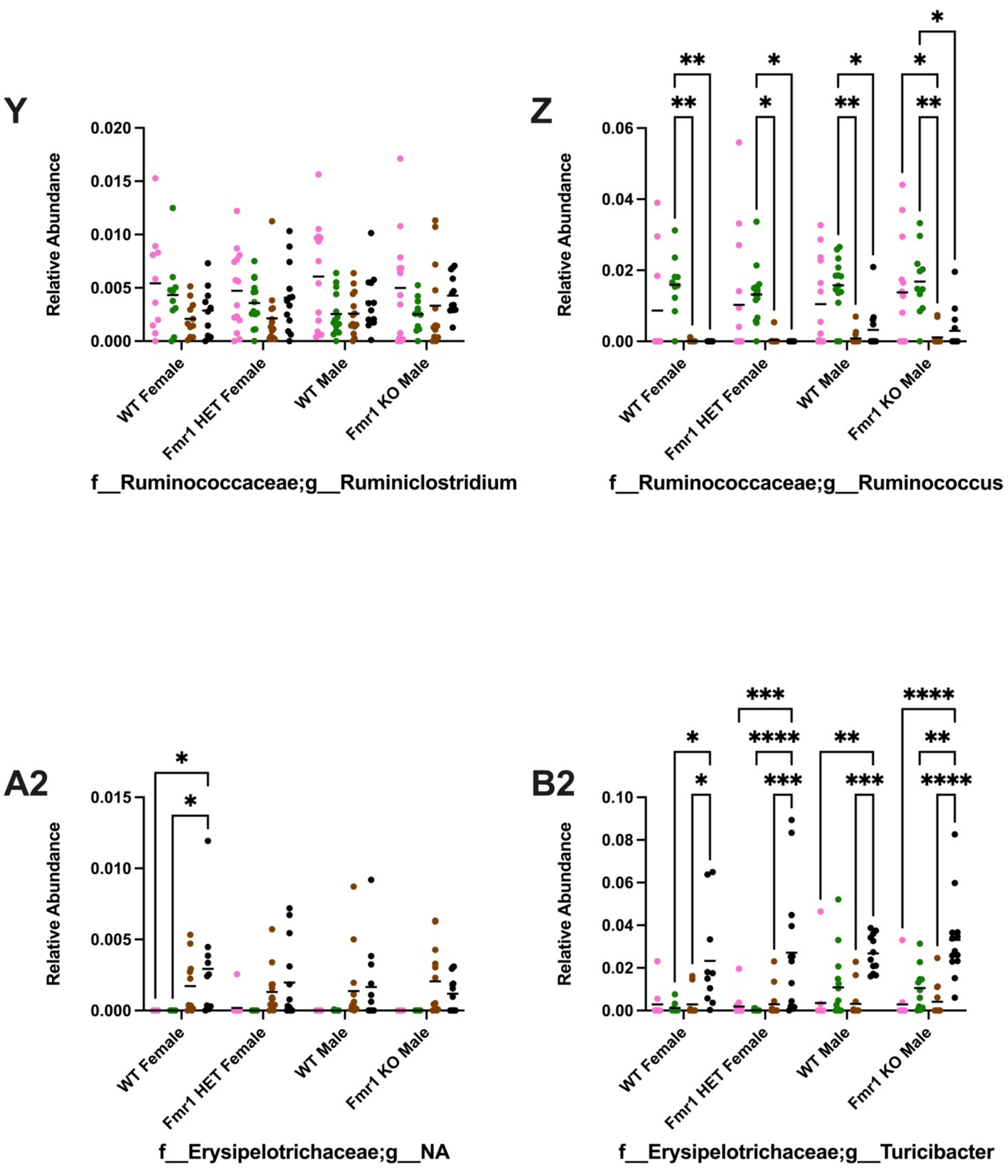
Y
0.020
0.015
0.010
0.005
0.000
Relative Abundance
WT Female
Fmr1 HET Female
WT Male
Fmr1 KO Male
f__Ruminococcaceae;g__Ruminiclostridium
Z
0.06
0.04
0.02
0.00
Relative Abundance
WT Female
Fmr1 HET Female
WT Male
Fmr1 KO Male
f__Ruminococcaceae;g__Ruminococcus
A2
0.015
0.010
0.005
0.000
Relative Abundance
WT Female
Fmr1 HET Female
WT Male
Fmr1 KO Male
f__Erysipelotrichaceae;g__NA
B2
0.10
0.08
0.06
0.04
0.02
0.00
Relative Abundance
WT Female
Fmr1 HET Female
WT Male
Fmr1 KO Male
f__Erysipelotrichaceae;g__Turicibacter

C2

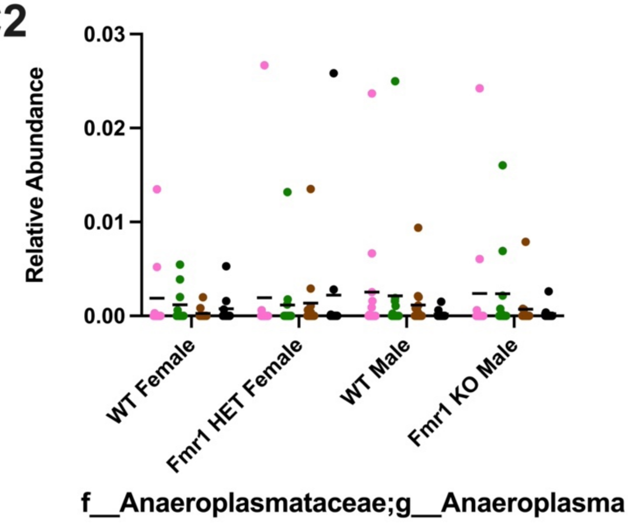
0.03
0.02
0.01
0.00
Relative Abundance
WT Female
Fmr1 HET Female
WT Male
Fmr1 KO Male
f__Anaeroplasmataceae;g__Anaeroplasma

D2

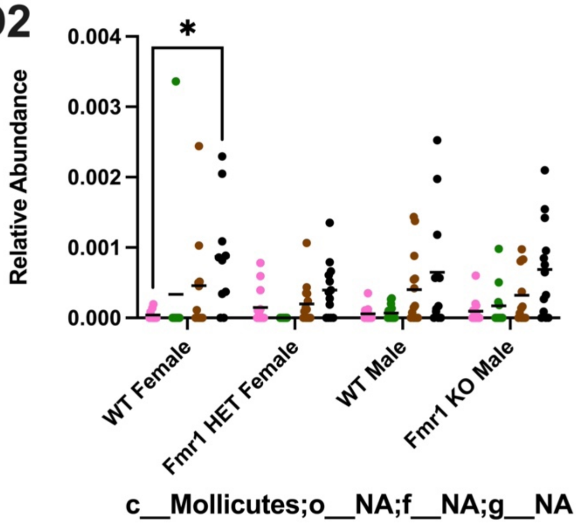
0.004
0.003
0.002
0.001
0.000
Relative Abundance
*
WT Female
Fmr1 HET Female
WT Male
Fmr1 KO Male
c__Mollicutes;o__NA;f__NA;g__NA

E2

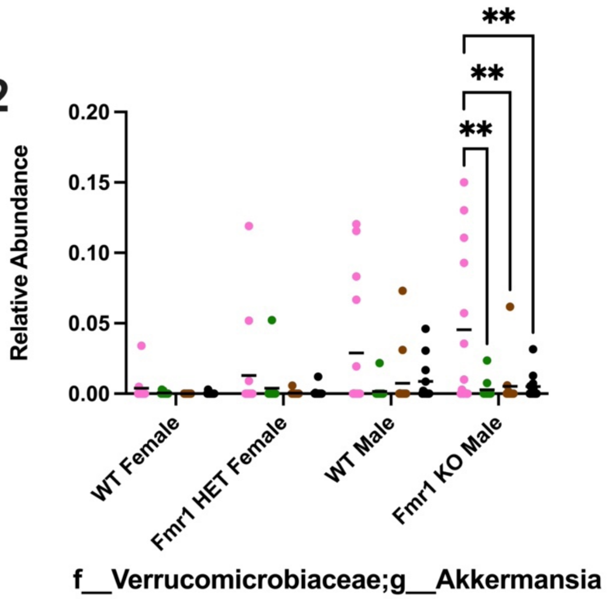
0.20
0.15
0.10
0.05
0.00
Relative Abundance
**
**
**
WT Female
Fmr1 HET Female
WT Male
Fmr1 KO Male
f__Verrucomicrobiaceae;g__Akkermansia

Supplementary Figure 10. Microbiome relative abundance at the species level as a function of genotype and diet for species that differ as a function of AIN-93G/soy diet. Relative abundance of positive reads out of the total number of reads after filtering (x-axis) is plotted versus genotype/diet for: **(A)** Ruminococcaceae;*g__NA;s__sp33140*, **(B)** Ruminococcaceae;*g__NA;s__sp34878*, **(C)** Ruminococcaceae;*g__NA;s__sp34879*, **(D)** Ruminococcaceae; *g__NA;s__sp34733*, and **(E)** Ruminococcaceae; *g__NA;s__sp35825*. Diets are color coded AIN-93G (pink), AIN-93G/soy (green), Teklad 2019 (brown), and Purina 5015 (black). Statistical significance was determined by two-way ANOVA with GraphPad Prism 10, ***p*<0.01, ****p*<0.001, *****p*<0.0001. Error bars comparing diets for each genotype are shown on the graphs. Abbreviations for titles on the x-axis: g=genus, s=species.

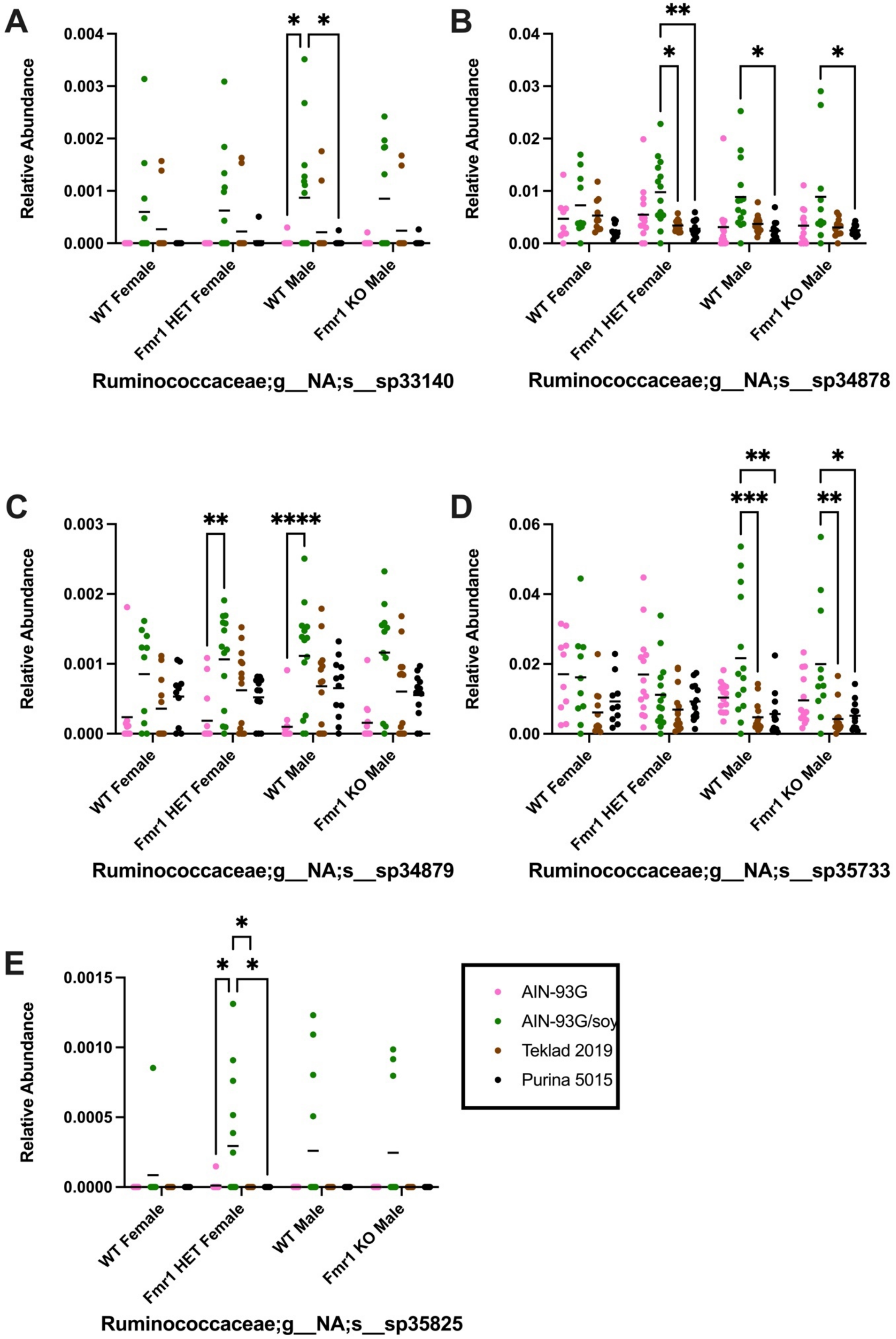
A
B
C
D
E
Relative Abundance
WT Female
Fmr1 HET Female
WT Male
Fmr1 KO Male
Ruminococcaceae;g__NA;s__sp33140
Ruminococcaceae;g__NA;s__sp34878
Ruminococcaceae;g__NA;s__sp34879
Ruminococcaceae;g__NA;s__sp35733
Ruminococcaceae;g__NA;s__sp35825
AIN-93G
AIN-93G/soy
Teklad 2019
Purina 5015

Supplementary Figure 11. Microbiome relative abundance at the species level as a function of genotype and diet for species that differ as a function of AIN-93G diet. Relative abundance of positive reads out of the total number of reads after filtering (x-axis) is plotted versus genotype/diet for Ruminococcaceae;*g__NA;s__sp34867*. Diets are color coded AIN-93G (pink), AIN-93G/soy (green), Teklad 2019 (brown), and Purina 5015 (black). Statistical significance was determined by two-way ANOVA with GraphPad Prism 10, ***p<0.001. Error bars comparing diets for each genotype are shown on the graphs. Abbreviations for titles on the x-axis: g=genus, s=species.

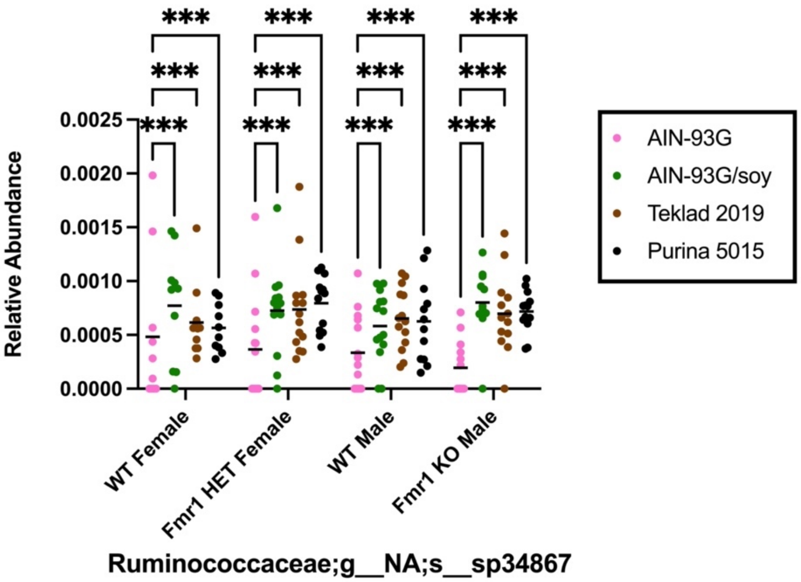

Supplementary Figure 12. Microbiome relative abundance at the species level as a function of genotype and diet for species that differ as a function of Teklad 2019 diet. Relative abundance of positive reads out of the total number of reads after filtering (x-axis) is plotted versus genotype/diet for: **(A)** Ruminococcaceae;*g__NA;s__sp34771*, and **(B)** Ruminococcaceae;*g__NA;s__sp35181*. Diets are color coded AIN-93G (pink), AIN-93G/soy (green), Teklad 2019 (brown), and Purina 5015 (black). Statistical significance was determined by two-way ANOVA with GraphPad Prism 10, **p*<0.05, ***p*<0.01, ****p*<0.001, *****p*<0.0001. Error bars comparing diets for each genotype are shown on the graphs. Abbreviations for titles on the x-axis: g=genus, s=species.

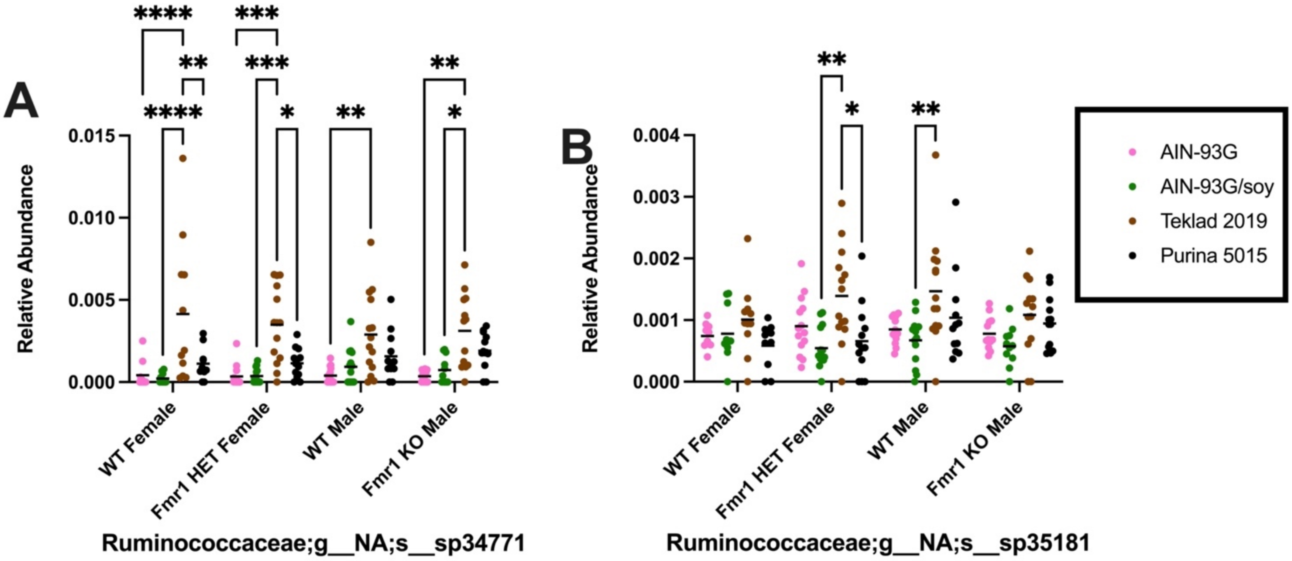

Supplementary Figure 13. Microbiome relative abundance at the species level as a function of genotype and diet for species that differ as a function of Purina 5015 diet. Relative abundance of positive reads out of the total number of reads after filtering (x-axis) is plotted versus genotype/diet for: **(A)** Ruminococcaceae;*g__NA;s__sp34750*, **(B)** Ruminococcaceae;*g__NA;s__sp35382*, and **(C)** Ruminococcaceae;*g__NA;s__sp35393-sp35424*. Diets are color coded AIN-93G (pink), AIN-93G/soy (green), Teklad 2019 (brown), and Purina 5015 (black). Statistical significance was determined by two-way ANOVA with GraphPad Prism 10, $^{**}p<0.01$. Error bars comparing diets for each genotype are shown on the graphs. Abbreviations for titles on the x-axis: g=genus, s=species.

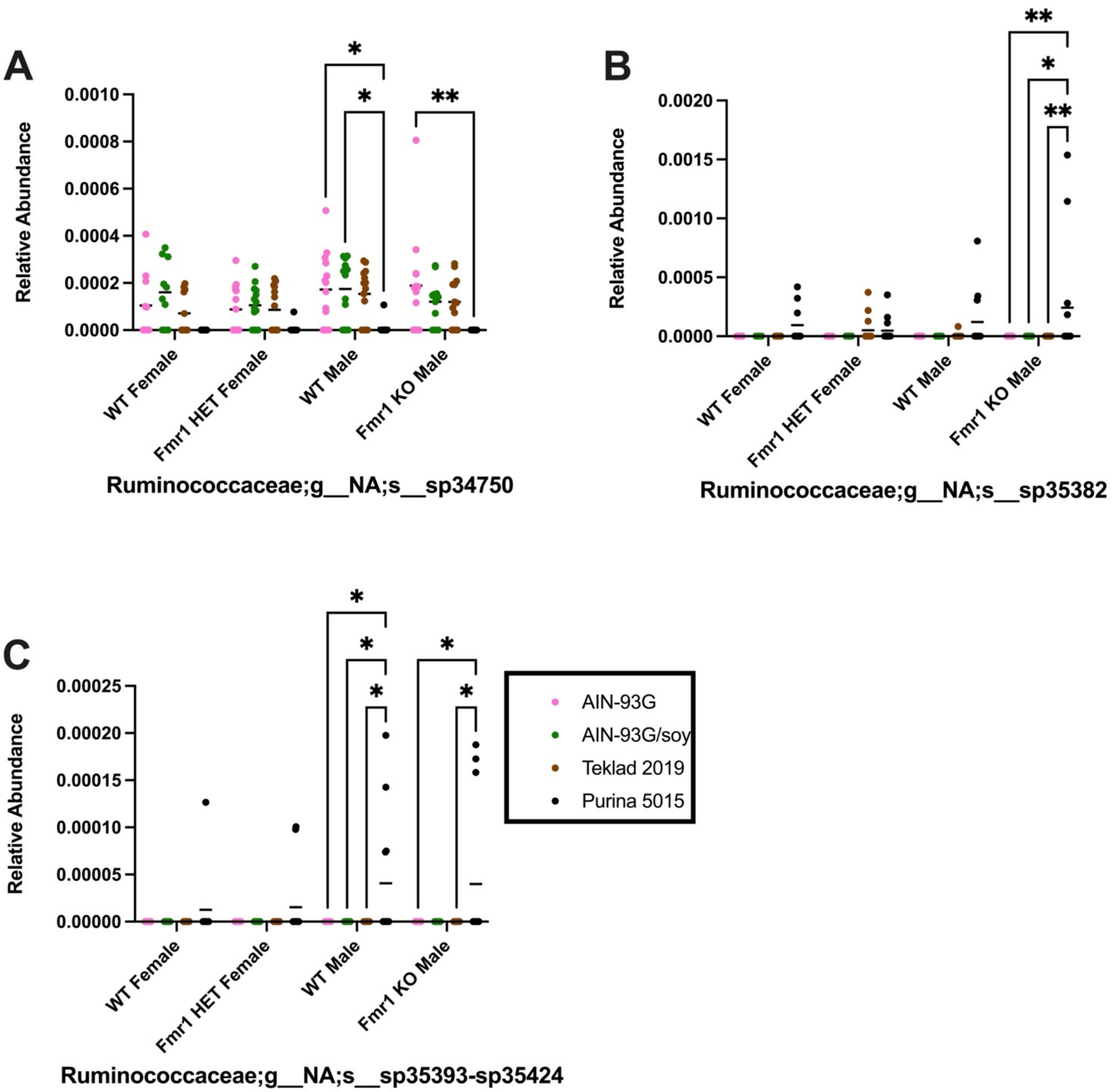

Supplementary Figure 14. Microbiome relative abundance at the species level as a function of genotype and diet for species that differ as a function of purified ingredient versus chow diet. Relative abundance of positive reads out of the total number of reads after filtering (x-axis) is plotted versus genotype/diet for: **(A)** Ruminococcaceae;*g__NA;s__sp34795-sp34820*, **(B)** Ruminococcaceae;*g__NA;s__sp34871*, **(C)** Ruminococcaceae;*g__NA;s__sp34878-sp34883*, **(D)** Ruminococcaceae; *g__NA;s__sp35494*, **(E)** Ruminococcaceae; *g__NA;s__sp35736*, and **(F)** Ruminococcaceae; *g__NA;s__sp35849*. Diets are color coded AIN-93G (pink), AIN-93G/soy (green), Teklad 2019 (brown), and Purina 5015 (black). Statistical significance was determined by two-way ANOVA with GraphPad Prism 10, *p<0.05, **p<0.01, ***p<0.001, ****p<0.0001. Error bars comparing diets for each genotype are shown on the graphs. Abbreviations for titles on the x-axis: g=genus, s=species.

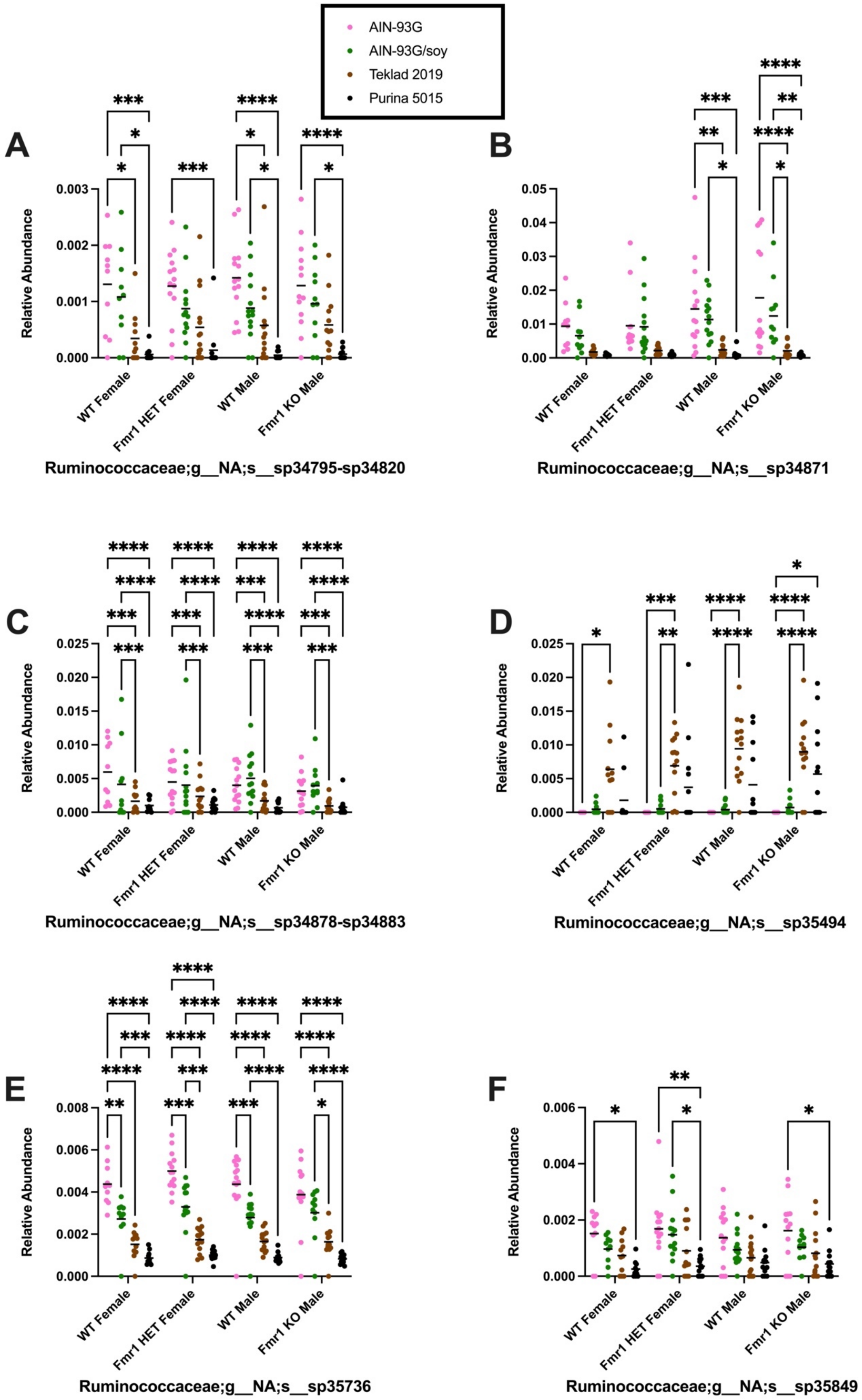
AIN-93G
AIN-93G/soy
Teklad 2019
Purina 5015
A
B
C
D
E
F
Relative Abundance
WT Female
Fmr1 HET Female
WT Male
Fmr1 KO Male
Ruminococcaceae;g__NA;s__sp34795-sp34820
Ruminococcaceae;g__NA;s__sp34871
Ruminococcaceae;g__NA;s__sp34878-sp34883
Ruminococcaceae;g__NA;s__sp35494
Ruminococcaceae;g__NA;s__sp35736
Ruminococcaceae;g__NA;s__sp35849

Supplementary Table 1: Average Plasma FITC-Dextran Levels as Function of Diet (µg/mL)				
**Genotype/Diet**	**AIN-93G**	**AIN-93G/soy**	**Teklad 2019**	**Purina 5015**
FVB Female WT	2.18 (n=9)	2.94 (n=10)	2.09 (n=11)	2.65 (n=10)
FVB Female *Fmr1*^{HET}	2.41 (n=16)	3.33 (n=14)	2.04 (n=17)	2.51 (n=13)
FVB Female *Fmr1*^{KO}	2.37 (n=5)	3.72 (n=2)	2.13 (n=7)	2.96 (n=2)
FVB Male WT	1.50 (n=10)	2.81 (n=14)	1.59 (n=14)	1.89 (n=11)
FVB Male *Fmr1*^{KO}	1.54 (n=13)	2.25 (n=11)	1.47 (n=13)	1.97 (n=13)

Supplementary Table 2: Average Body Weight as Function of Diet (g) (FITC mice)				
**Genotype/Diet**	**AIN-93G**	**AIN-93G/soy**	**Teklad 2019**	**Purina 5015**
FVB Female WT	22.99 (n=10)	22.43 (n=10)	21.56 (n=11)	23.68 (n=10)
FVB Female *Fmr1*^{HET}	21.74 (n=16)	22.51 (n=15)	22.25 (n=17)	22.37 (n=13)
FVB Female *Fmr1*^{KO}	21.82 (n=5)	19.31 (n=2)	22.06 (n=7)	25.11 (n=2)
FVB Male WT	25.83 (n=14)	26.65 (n=15)	25.40 (n=15)	29.21 (n=11)
Male *Fmr1*^{KO}	28.25 (n=13)	26.75 (n=11)	25.52 (n=13)	28.94 (n=13)

Supplementary Table 3: Average Body Weight as Function of Diet (g) (all mice)				
**Genotype/Diet**	**AIN-93G**	**AIN-93G/soy**	**Teklad 2019**	**Purina 5015**
FVB Female WT	23.25 (n=18)	21.84 (n=22)	21.52 (n=26)	23.61 (n=25)
FVB Female *Fmr1*^{HET}	22.29 (n=36)	22.07 (n=37)	21.47 (n=51)	23.28 (n=32)
FVB Female *Fmr1*^{KO}	20.99 (n=9)	20.67 (n=4)	22.07 (n=14)	23.70 (n=6)
FVB Male WT	29.29 (n=21)	26.99 (n=25)	24.87 (n=36)	29.34 (n=17)
FVB Male *Fmr1*^{KO}	27.88 (n=23)	29.57 (n=16)	25.43 (n=32)	29.30 (n=22)

Supplementary Table 4: Average Plasma FITC-Dextran Levels as Function of Diet (µg/mL)			
**Genotype/Diet**	**AIN-93G**	**AIN-93G/soy**	**Teklad 2019**
C57BL/6J Female *Fmr1*^{KO}	1.56 (n=9)	9.43 (n=12)	3.17 (n=13)
C57BL/6J Male *Fmr1*^{KO}	1.53 (n=7)	9.68 (n=12)	3.41 (n=13)

Supplementary Table 5: Average Body Weight as Function of Diet (g) (FITC mice)			
**Genotype/Diet**	**AIN-93G**	**AIN-93G/soy**	**Teklad 2019**
C57BL/6J Female *Fmr1*^{KO}	20.94 (n=9)	19.79 (n=12)	20.69 (n=13)
C57BL/6J Male *Fmr1*^{KO}	26.19 (n=7)	24.89 (n=12)	26.43 (n=13)

Supplementary Table 6: Average Body Weight as Function of Diet (g) (all mice)			
**Genotype/Diet**	**AIN-93G**	**AIN-93G/soy**	**Teklad 2019**
C57BL/6J Female *Fmr1*^{KO}	21.31 (n=11)	19.62 (n=24)	20.67 (n=20)
C57BL/6J Male *Fmr1*^{KO}	25.37 (n=9)	25.29 (n=17)	26.08 (n=23)

Supplementary Table 7: LEfSe Summary Results	
**AIN-93G**	
WT females	No significant differences
Fmr1^{HET} females	No significant differences
WT males	No significant differences
Fmr1^{KO} males	No significant differences
**AIN-93G/soy**	
WT females	No significant differences
Fmr1^{HET} females	No significant differences
WT males	No significant differences
Fmr1^{KO} males	No significant differences
**Teklad 2019**	
WT females	k__Bacteria.p__Firmicutes
Fmr1^{HET} females	k__Bacteria.p__Bacteroidetes.c__Bacteroidia.o__Bacteroidales.f__NA.g__NA.s__sp12473_sp12526_sp12633
	k__Bacteria.p__Bacteroidetes.c__Bacteroidia.o__Bacteroidales.f__NA.g__NA.s__sp12473_sp12526
WT males	k__Bacteria.p__Bacteroidetes
	k__Bacteria.p__Bacteroidetes.c__Bacteroidia
	k__Bacteria.p__Bacteroidetes.c__Bacteroidia.o__Bacteroidales
	k__Bacteria.p__Bacteroidetes.c__Bacteroidia.o__Bacteroidales.f__NA
	k__Bacteria.p__Bacteroidetes.c__Bacteroidia.o__Bacteroidales.f__NA.g__NA
	k__Bacteria.p__Bacteroidetes.c__Bacteroidia.o__Bacteroidales.f__NA.g__NA.s__sp12656
Fmr1^{KO} males	k__Bacteria.p__Proteobacteria.c__Gammaproteobacteria
**Purina 5015**	
WT females	k__Bacteria.p__Actinobacteria.c__Coriobacteriia.o__Coriobacteriales.f__Coriobacteriaceae.g__Enterorhabdus.s__caecimuris
	k__Bacteria.p__Firmicutes.c__Clostridia.o__Clostridiales.f__Lachnospiraceae.g__Roseburia.s__sp33193
Fmr1^{HET} females	k__Bacteria.p__Firmicutes.c__Clostridia.o__Clostridiales.f__Ruminococcaceae.g__Oscillibacter.s__sp34638
WT males	k__Bacteria.p__Firmicutes.c__Clostridia.o__Clostridiales.f__Lachnospiraceae.g__Roseburia.s__intestinalis_sp33137
Fmr1^{KO} males	No significant differences

Supplementary Table 8: Comparison of Gut Permeability and Microbiome Data in Other Studies Using *Fmr1*^{KO} Models		
**Study**	**Model**	**Findings**
Altimiras, 2021	C57BL/6J *Fmr1*^{KO2} mice	The most prevalent phyla identified were Bacteroidetes (54%) and Firmicutes (36%).<br><br>Found no difference in alpha diversity.<br><br>Found significant differences in 7 phyla (Actinobacteria, Bacteroidetes, Cyanobacteria, Firmicutes, Proteobacteria, Tenericutes and Verrucomicrobia) and 10 genera (*Allobaculum*, *Akkermansia*, *Bacteroides*, *Bifidobacterium*, *Desulfovibrio*, *Flexispira*, *Odoribacter*, *Oscillospira*, *Sutterella* and *Turicibacter*) including increased *Akkermansia*.
Goo, 2020	C57BL/6J *Fmr1*^{KO} mice	Found significantly reduced abundance of *Akkermansia_muciniphila* (~90%), which was rescued by fecal microbiota transplantation from WT mice.
Guo, 2023	C57BL/6J *Fmr1*^{KO} mice	The most prevalent phyla were Firmicutes (50%) and Bacteroidota (40%) in WT mice and Firmicutes (50%) and Bacteroidota (45%) in *Fmr1*^{KO} mice.<br><br>The Shannon Index was not statistically different.<br><br>Principal component analysis showed different clusters in beta-diversity.<br><br>The mRNA and protein levels for tight junction protein 3 (TJP3) are decreased in the colon of *Fmr1*^{KO} mice compared to WT with no differences in other tight junction protein mRNAs.<br><br>No difference in FITC-dextran intestinal permeability after a four hour fast (~1.7 µg/mL).
Rude, 2019	*FMR1* 170-200 CGG repeats mice	Found genotype effect on gut permeability after PCB exposure.<br><br>The most abundant phyla were Firmicutes (~55%) and Bacteriodetes (~38).
Salmerón, 2024	*Fmr1*^{KO} Sprague-Dawley rats	Found altered betaine and amino acid levels.
Luhur, 2017	*dfmr1* null Drosophila	FMRP is enriched in intestinal progenitor cells and limits the symmetric division and resulting expansion of the stem cell population during adaptive intestinal growth. Adult *Fmr1* null mutants have an increased number of progenitor cells and enlarged intestines.
Lee, 2023	*Drosophila*	Identified *dfmr1* as an essential gene for intestinal stem cell proliferation during gut damage using knockdown RNA interference. *Fmr1* transcript expression is upregulated in response to drugs that induce gut damage.
